# The *Plasmodiophora brassicae* genome reveals insights in its life cycle and ancestry of chitin synthases

**DOI:** 10.1038/srep11153

**Published:** 2015-06-18

**Authors:** Arne Schwelm, Johan Fogelqvist, Andrea Knaust, Sabine Jülke, Tua Lilja, German Bonilla-Rosso, Magnus Karlsson, Andrej Shevchenko, Vignesh Dhandapani, Su Ryun Choi, Hong Gi Kim, Ju Young Park, Yong Pyo Lim, Jutta Ludwig-Müller, Christina Dixelius

**Affiliations:** 1 Swedish University of Agricultural Sciences, Department of Plant Biology, Uppsala BioCenter, Linnean Centre for Plant Biology, P.O. Box 7080, SE-75007 Uppsala, Sweden; 2 Institute of Botany, Technische Universität Dresden, 01062 Dresden, Germany; 3 Max Planck Institute of Molecular Cell Biology and Genetics, Pfotenhauerstrasse 108, 01307 Dresden, Germany; 4 Swedish University of Agricultural Sciences, Department of Microbiology, Uppsala BioCenter, P.O. Box 7025, SE-75007 Uppsala, Sweden; 5 Swedish University of Agricultural Sciences, Department of Forest Mycology and Plant Pathology, Uppsala BioCenter, P.O. Box 7026, SE-75007 Uppsala, Sweden; 6 Department of Horticulture, College of Agriculture and Life Sciences, Chungnam National University, Gungdong 220, Yuseong-gu, Daejeon, 305–764, Republic of Korea; 7 Department of Applied Biology, College of Agriculture and Life Sciences, Chungnam National University, Gungdong 220, Yuseong-gu, Daejeon, 305–764, Republic of Korea

## Abstract

*Plasmodiophora brassicae* causes clubroot, a major disease of *Brassica* oil and vegetable crops worldwide. *P. brassicae* is a Plasmodiophorid, obligate biotrophic protist in the eukaryotic kingdom of Rhizaria. Here we present the 25.5 Mb genome draft of *P. brassicae,* developmental stage-specific transcriptomes and a transcriptome of *Spongospora subterranea,* the Plasmodiophorid causing powdery scab on potato. Like other biotrophic pathogens both Plasmodiophorids are reduced in metabolic pathways. Phytohormones contribute to the gall phenotypes of infected roots. We report a protein (PbGH3) that can modify auxin and jasmonic acid. Plasmodiophorids contain chitin in cell walls of the resilient resting spores. If recognized, chitin can trigger defense responses in plants. Interestingly, chitin-related enzymes of Plasmodiophorids built specific families and the carbohydrate/chitin binding (CBM18) domain is enriched in the Plasmodiophorid secretome. Plasmodiophorids chitin synthases belong to two families, which were present before the split of the eukaryotic Stramenopiles/Alveolates/Rhizaria/Plantae and Metazoa/Fungi/Amoebozoa megagroups, suggesting chitin synthesis to be an ancient feature of eukaryotes. This exemplifies the importance of genomic data from unexplored eukaryotic groups, such as the Plasmodiophorids, to decipher evolutionary relationships and gene diversification of early eukaryotes.

Clubroot caused by *Plasmodiophora brassicae*, is a spreading soil-borne disease with high economical impact on *Brassica* oil- and vegetable crops and production of other valuable species within the family *Brassicaceae* worldwide^1^. *P. brassicae* is distinct from other plant pathogens, such as fungi or oomycetes, as it is an obligate biotroph protist in the Plasmodiophorids within the eukaryote supergroup Rhizaria. Rhizaria share a common ancestor with Stramenopiles and Alveolata forming the S-A-R supergroup, which together with Plantae build the SARP megagroup^2^,^3^. Plasmodiophorids are parasites of plants and oomycetes, and a sister group to Phagomyxids, pathogens of sea grass, diatoms, and brown algae^4^. Other agriculturally important Plasmodiophorid pathogens are *Spongospora subterranea,* the causal agent of potato powdery scab and root galls (Supplementary Fig. S1) and vector for the Potato Mop Top Virus, and the virus transmitting *Polymyxa betae* and *P. graminis* infecting sugar beets and cereals.

Plasmodiophorids have a complex, not completely understood, life cycle^5^ consisting of different zoosporic stages, formation of plasmodia inside host cells, and resting spore formation (Fig. 1). The haploid resting spore releases a zoospore, which infect the root hairs and form multinucleate plasmodia. Those develop several single-nucleate secondary zoospores, which are released into the soil. A fusion of those secondary zoospores is assumed to occur occasionally. Zoospores invade the root cortex, in which secondary multinucleate plasmodia develop. How and when karyogamy is taking place is not exactly known, but meiosis appears to occur in the plasmodia before resting spore formation.

Plasmodia provoke abnormal cell enlargement (hypertrophy) and uncontrolled cell division (hyperplasia) leading to the development of galls (Supplementary Fig. S1) that obstruct nutrient and water transport. In infected root tissue, different developmental stages of the Plasmodiophorids occur simultaneously. For example, host cells containing plasmodia neighbor cells filled with resting spores. The only host-free stages are the short-lived bi-flagellated zoospores and resting spores. Therefore molecular and genomic data for Plasmodiophorids are rare as they only grow inside living host cells and remain uncultivable on their own.

Furthermore, the Rhizaria is the least studied group of eukaryotes^6^,^7^ with only the genomes of the chlorarachniophyte *Bigelowiella natans*^8^ and the foraminifer *Reticulomyxa filosa*^9^ available until now. To fill this gap, we sequenced, assembled and analyzed the genome and transcriptomes of *P. brassicae*, to create the first genome draft of a pathogenic Rhizaria. The single-spore isolate e3^10^, for which no sexual recombination has been observed, was used to avoid complication due to potential polyploidy and chromosome polymorphism. We emphasized on generating transcriptomes of life-stage specific materials from *P. brassicae* which enabled analyses of developmental stage specific gene expression. The genome information of *P. brassicae* was further used to identify and analyze transcript data from the related potato pathogen *S. subterranea*.

Resting spores of *P. brassicae* are extremely resilient to harsh environmental conditions, and contaminate arable land for decades. This feature makes it impossible to eradicate the organism via any known chemical or alternative soil treatment. The resting spores which are formed at the end of the life cycle inside the root contain chitin in their cell walls^11^. Besides being an important component of the Plasmodiophorid survival structure, chitin and other glycans are known as important signatures for plant immune activation or for the establishment of beneficial symbioses^12^. We analyzed the chitin-related proteins detected in the Plasmodiophorids in more detail and also the secretome for effector candidates that could interfere with microbe-triggered host responses.

## Results and Discussion

### Genome sequencing and Rhizaria genome comparisons

We sequenced the *P. brassicae* genome from genomic DNA of resting spores from the single spore isolate e3 (Supplementary Table S1). The 24 Mb genome was *de novo* assembled into 165 scaffolds with an N50 size of 473 kb, and an average 500-fold coverage (Supplementary Fig. S2; Supplementary Table S2). The details of the sequencing analysis are provided in the supplementary material (Supplementary Note). The GC-content (58.5%) is higher than for *B. natans* (46%) and *R. filosa* (35%). The genome is small compared to the free-living Rhizaria *B. natans* (~100 Mb) and *R. filosa* (~320 Mb) but larger than earlier suggested (20.3 Mb)^13^. A total of 9,730 genes were predicted with support from seven transcriptome libraries (Supplementary Table S3) representing 66% of the genome with an average of 404 genes per Mb. *P. brassicae* genes contain an average of 4 introns per gene of 60bp length. With only 5,4% the detected content of repetitive and transposable elements, including new identified and previously described repeats (AF296444, AF296445, AF537105)^10^, is low (Supplementary Fig. S3; Supplementary Table S4). The low number of repeat is concurrent with a total genome size estimation of 25.5 Mb based on 17-mer analysis. The gene density of *P. brassicae* (Supplementary Figs. S3, S4), lower number of genes (Supplementary Fig. S5), and low content of repetitive elements, contribute to the smaller genome size compared to the free-living *B. natans* and *R. filosa*. Similar genome reductions are observed for other eukaryotic parasites compared to free-living species^14^.

CEGMA evaluation^15^ revealed that 92% of the eukaryotic core genes were of full length (94% partially), which is slightly higher compared to the other two Rhizarian genomes (Supplementary Table S5). The *S. subterranea* transcriptome was assembled into a total of 12,732 protein models of which 7,490 were full-length, covering 69% of CEGMA genes. The higher number compared to *P. brassicae* is likely due to partial assembled genes or might reflect current or recent transposon activity as suggested earlier^16^.

**Figure 2:**
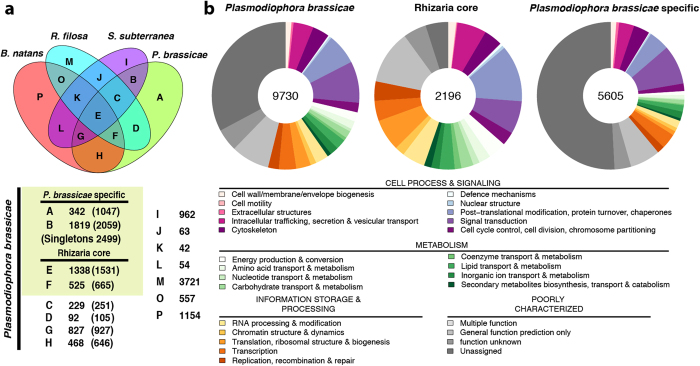
Protein families in Rhizaria and *P. brassicae*. (**a**) Venn diagramm of OrthoMCL protein families predicted for *P. brassicae*, *S. subterranea* and the the non-pathogenic Rhizarian *B. natans* and *R. filosa*. A Rhizarian core set was defined by common familes of *B. natans*, *R. filosa* and *P. brassicae* (groups E + F). Groups A-D build *P. brassicae* specific proteins. The *S. subterranea* predicted models were not regarded for the definition of the core set and *P. brassicae* specific set as no complete genome information is available. Numbers in the table show the number of OrthoMCL families and in brackets the number of *P. brassicae* proteins in each family. (**b**) Functional annotation of the *P. brassicae* proteins according to KOG functional categories. Rhizaria core set and *P. brassicae*-specific genes are defined as in (**a**).

OrthoMCL^17^ analysis defined a Rhizaria core set of 1,863 protein families common to *P. brassicae*, *R. filosa* and *B. natans* of which 1,338 families were shared with *S. subterranea* (Fig. 2). The specific set of *P. brassicae* contained 2,161 protein families out of 5,605 proteins with no orthologues in *R. filosa* and *B. natans.* KOG-term analysis showed that high proportions of the proteins in the Plasmodiophorids are poorly characterized and the majority of the *P. brassicae* specific genes have no assigned function (Fig. 2; Supplementary Fig. S5). The *P. brassicae*-specific genes were diversely expressed in the different developmental stages, whereas the Rhizarian core genes were most active in the plasmodial stage, when *P. brassicae* was already established inside the plant root (Supplementary Fig. S6).

Compared to *R. filosa* and *B. natans*, fungi and plant pathogenic oomycetes, the two Plasmodiophorids were enriched for the carbohydrate esterase family 4 (CE4, polysaccharide deacetylases), galactose-binding lectins, ankyrin domains, the carbohydrate/chitin binding domain CBM18 and RAP RNA-binding domain proteins (Supplementary Fig. S7; Supplementary Table S6). The Gal-lectin domain is proposed to be of animal origin and acquired by the fish pathogenic oomycete *Saprolegnia parasitica* via horizontal gene transfer^18^. However, our analyses of Gal-lectin domains including SARP and Amoebozoa data suggesting a more ancient origin of these proteins (Supplementary Fig. S8). Unlike *P. brassicae*, the *S*. *subterranea* transcriptome was also enriched in transposon and virus-related genes (Supplementary Table S6), which has been observed earlier^16^.

It is not known if RNA-directed gene silencing occurs in Plasmodiophorids. *P. brassicae* and *S. subterranea* contain argonautes but only one gene each encoding for a protein with two RNaseIII domains and a dsRNA-binding motif (Supplementary Fig. S9). Those proteins resemble the Dicer-like protein 1 from the ciliate protozoan *Tetrahymena thermophile* and Drosha proteins of *Drosophila melanogaster* involved in miRNA processing. Any predicted RNA-dependent RNA polymerase, and RNA polymerase IV were not detected in *P. brassicae.*

### Metabolism shows biotrophic signatures

Similar to other eukaryotic biotrophic plant pathogens^19^,^20^,^21^ *P. brassicae* and *S. subterranea* are reduced in several metabolic pathways. Both Plasmodiphorids lack genes encoding for proteins involved in sulfur and nitrogen uptake as well as genes for histidine, tryptophan and threonine biosynthesis. Arginine and lysine synthesis were also compromised in *P. brassicae* (Supplementary Figs. S10, S11). Thiamine biosynthesis genes were also reduced in the Plasmodiophorids (Supplementary Fig. S12), suggesting a dependence of their host plants for those metabolites. No fatty acid synthase was identified in *P. brassicae* (Supplementary Fig. S13). The inability for fatty acid synthesis has been described for obligate biotrophic bacterial pathogens of insects and plants^22^, and kinetoplast parasites such as *Trypanosoma* species. The *P. brassicae* gene content for fatty acid biosynthesis was similar as for the kinetoplasts, which use an alternative parasitic-specific fatty acid synthesis, the microsomal elongase pathway^23^. Our data suggest that *P. brassicae* metabolize fatty acids from the host by elongating and modifying molecules of four or more carbons. Protein and transcript data showed that fatty acids were synthesized in the plasmodia and degradation occurred in the spores (Supplementary Figs. S14, S15). Seven putatively secreted lipases in the *P. brassicae* genome were among the highest expressed genes in the plasmodial stage providing the pathogen with fatty acid starter molecules. However, in the potato scab transcriptome, we detected two potential fatty acid synthase genes and no secreted lipases, suggesting that *S. subterranea* synthesizes fatty acids *de novo.*

We predicted carbohydrate conversion to mainly occur via glycolysis, glyoxylate, pyruvate metabolism, pentose phosphate pathway and the TCA cycle (Supplementary Figs. S14–16). Proteins and transcripts of the glyoxylate cycle were highly abundant in the germinating spore sample indicating a high conversion of sugars and lipids in this active life-stage. The *P. brassicae* genome contained trehalose biosynthesis genes expressed in all transcriptome sets (Supplementary Fig. S17), which corroborates to earlier findings of trehalose accumulation by *P. brassicae* in infected plants^24^. It has been speculated that trehalose production by plant pathogens interferes with the sugar sensing pathways of their host and thereby manipulate plant metabolism to their favor or regulate energy and nitrogen supply during colonization^25^. Besides affecting the host, trehalose in the resting spores could be important for their long-term survival as an energy resource or an osmoprotectant against the effects of desiccation. A trehalase-encoding gene of *P. brassicae* was primarily expressed in spores indicating a release of glucose as an energy resource in the germination process.

### Plant hormone homeostasis

**Figure 3:**
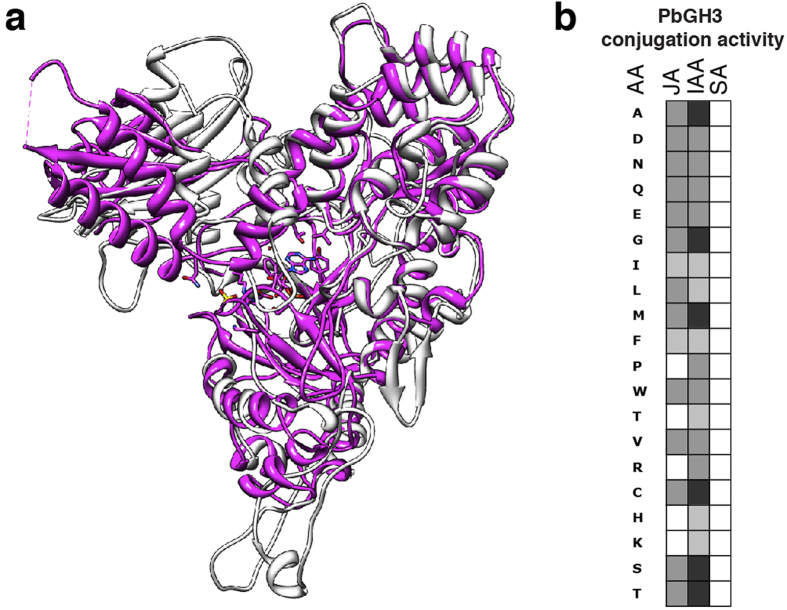
Structure prediction and activity of the PbGH3 protein. (**a**) Modeling of PbGH3 on plant GH3-proteins for which crystal structures are available. An overlay of the obtained protein model for PbGH3 (gray) with a monomer of the *A. thaliana* AtGH3.12 (PDB database entry 4EPM (http://www.rcsb.org/pdb/home/home.do) protein (magenta) is shown. The model was built in SWISS MODEL^65^ and the overlaid with the standard settings of Chimera^66^. (**b**) Conjugation activity of PbGH3 with IAA, SA and JA and amino acids (AA). Activity is indicated by black boxes (high activity) to white boxes (no activity).

Plant tissue infected with *P. brassicae* shows a modified hormone balance^26^,^27^ that induces hypertrophy and cell divisions in host roots leading to gall formation. *P. brassicae* can potentially modify the plant hormone homeostasis of at least four plant hormones: cytokinins^26^ and salicylic acid (SA)^28^ and, as shown here, auxins and jasmonic acid (JA). The *P. brassicae* genome, but not the *S. subterranea* transcripts, contained an auxin-responsive Gretchen Hagen 3 (GH3) protein (PbGH3). GH3-proteins are known to maintain hormonal homeostasis by conjugating plant hormones to amino acids^29^. The PbGH3 predicted protein structure resembles plant GH3-proteins (Fig. 3a). The heterologous expressed PbGH3 protein conjugated auxins and JA with proteinogenic amino acids *in vitro*, although it is phylogenetically not related to plant GH3-like proteins (Fig. 3b, Supplementary Fig. S18). Both Plasmodiophorids contained two isopentenyl-transferases (IPTs) belonging to DMAPP:tRNA-IPTs classes I and II (Supplementary Fig. S19). Class I DMAPP:tRNA-IPTs include members from *Physcomitrella patens* and *Arabidopsis thaliana* that are involved in *cis*-zeatin synthesis^30^. Cytokinin homestasis can also be inferred by a *P. brassicae* cytokinin oxidase, although the expression in our transcript sets was low (Supplementary Fig. S19). *P. brassicae* produces *trans-*zeatin^26^ and the contribution of the Plasmodiophorid IPTs and the potential cytokinin oxidase to the host cytokinin homeostasis remains to be shown. The gall phenotype might be a result of pathogen-driven interference of the plant root phytohormone system combined with reprogramming of existing meristematic activity^31^. The recently described secreted methyl transferase (PbBSMT) can reduce the accumulation of SA in infected roots^28^. *PbBSMT* expression in spores and plasmodia was low, but high expression levels in clubroots of *Brassica napus*, *B. oleracea* and *B. rapa* host plants highlights its important role (Supplementary Fig. S20). A putative homolog was also found in *S. subterranea* indicating a conserved Plasmodiophorid strategy to interfere with plant host SA-defense responses.

### The Plasmodiophorid secretome

Microbes secrete effector proteins to manipulate host processes in order to facilitate colonization. We predicted 553 secreted proteins in *P. brassicae* and 613 in *S. subterranea*. Both secretomes were smaller than that of *B. natans* but similar in numbers to other biotrophic fungi and oomycete plant pathogens (Supplementary Fig. S21). An OrthoMCL comparison of the two secretomes revealed that most secreted proteins were not shared between the two species (Supplementary Fig. S21) but were enriched in ankyrin and protein domains detected in effectors of other plant pathogens^32^, such as small cysteine-rich secreted proteins, chitin recognition proteins, proteases and protease inhibitors (Supplementary Table S7). The secretome of *P. brassicae* was further enriched in leucine–rich repeat domains and pathogenesis-related (PR) proteins, such as PR-5 thaumatin like-proteins. A cysteine-rich secretory protein with BLASTP hits to PR-1-like proteins with no homologues in the non-plant pathogenic Rhizarians, was also present in *S. subterranea*, and identified in the Plasmodiophorid *P. betae* (Pbef2)^33^.

Oomycete effectors frequently code for host-targeting sequence motifs, such as RXLR, LXLFLAK, and CHXC^34^. The secreted proteins of *P. brassicea* and *S. subterranea* do not contain such overrepresented amino acid motifs (Supplementary Table S8). Instead, we categorized the *P. brassicae* secretome into three groups based on their gene expression to identify potential effectors: 1) the PLeff genes - highest expressed in plasmodia, 2) the Heff genes – highest expressed in the transcriptomes obtained from the hosts, and 3) the Leff genes – highest expressed late in the life cycle during spore formation (Supplementary Fig. S21; Supplementary Table S9). Each group contains proteins that could reprogram the plant metabolism and interfere with the plant defense on a protein level. Highly expressed PLeff genes contained ankyrin domains, which are frequent in all Rhizarian^8^,^9^, but are effector candidates for bacterial pathogens and symbionts^35^,^36^. PLeff genes also included a secreted lipase, leucine-rich repeats proteins, PBRA_T005609, that shows similarities to effector candidates of biotrophic smut fungi in the Ustilagomycetes^37^, and RING finger domain containing proteins. Secreted RING finger domain proteins interfere with the host ubiquitation system of the human intracellular pathogen *T. cruz*i^38^, a strategy also seen for plant pathogens^32^. *PbBSMT* is one of the highest expressed genes in the Heff group, likely reducing the SA content in infected host tissue. The Leff candidates include CE4-domain polysaccharide acetylases, proteases and protease inhibitors including Kazal-like and papain protease inhibitors, and fascilin domain proteins.

### Carbohydrate-Active enZymes (CAZymes)

**Figure 4:**
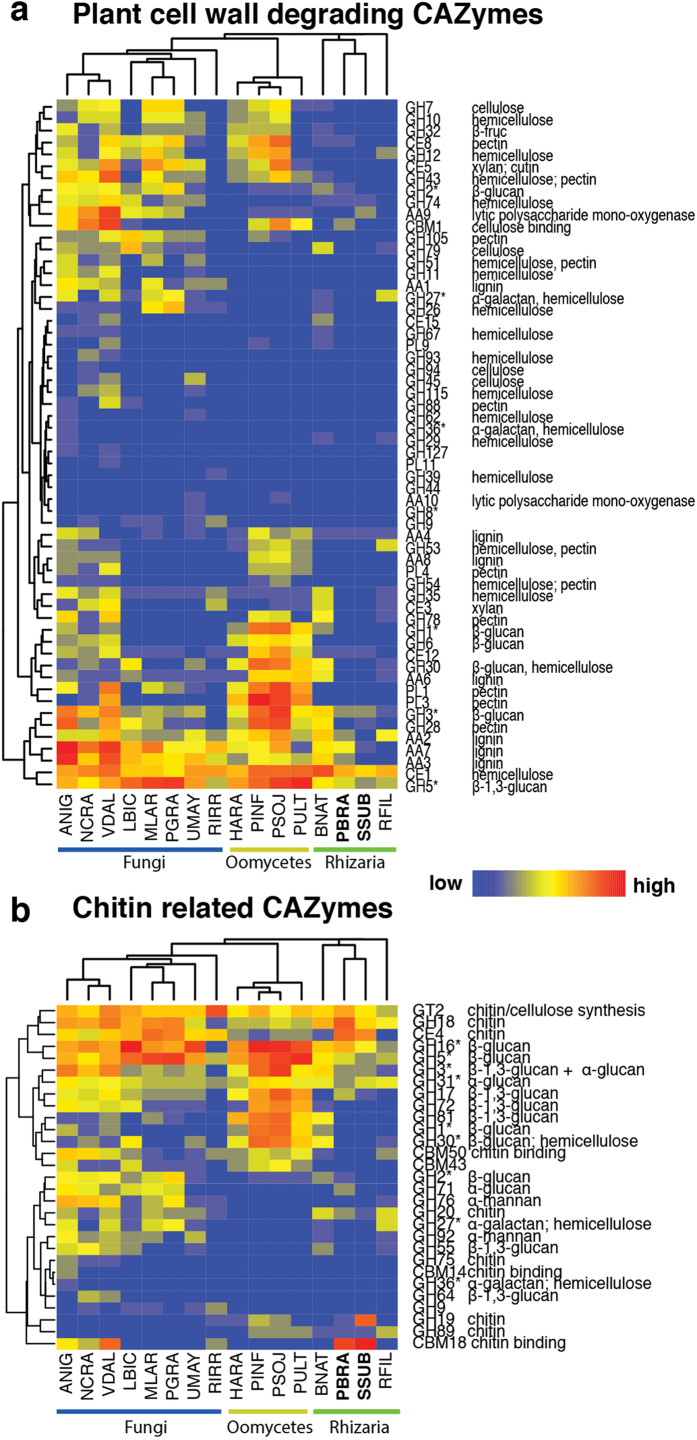
Distribution of (**a**) plant cell wall degrading enzymes, and (**b**) chitin related enzymes in Rhizarian and selected fungal and oomycete genomes. The heatmap represents the abundance of CAZyme domains in each genome. Clustering of gene families is based on Pearson correlation of gene numbers in each family in each species. *Indicates CAZymes with a possible effect on polysaccharides contained in both chitinous and/or plant cell walls. Species abbreviations: HARA = *Hyaloperonospora arabidopsidis*, PINF = *Phytophthora infestans*, PSOJ = *Phytophthora sojae*, PULT = *Pythium ultimatum*. BNAT = *Bigellowiella natans,* PBRA = *Plasmodiophora brassicae*, SSUB = *Spongospora subterranea*, RFIL = *Reticulomyxae filosa*, ANIG = *Aspergillus nigra*, NCRA = *Neurospora crassa*, VDAL = *Verticillium dahliae,* LBIC = *Laccaria bicolor*, MLAR = *Melampsora larici-populina,* PGRA = *Puccinia graminis* f. sp. *tritici*, UMAY = *Ustilago maydis*, RIRR = *Rhizophagus irregularis*.

Like other biotrophic plant pathogens^19^,^20^,^21^,^39^ the Plasmodiophorids have a reduced set of CAZymes (Supplementary Fig. S22). The total number of CAZymes was similar to those identified in the genomes of *Hyaloperonospora arabidopsidis, Ustilago maydis* or the arbuscular mycorhizal fungus *Rhizophagus irregularis.* However, the arsenal for both potential plant cell wall (PCW) modifying and chitin cell wall modifying enzymes, differed substantially from fungal and oomycete species (Fig. 4). In the proteins models of *P. brassicae* and *S. subterranea* only a few domains were identified that could degrade hemicellulose. Predicted CAZymes with domains involved in cellulose, lignin or pectin degradation or their modifications were also rare, compared to selected fungi, oomycetes, *B. natans* and *R. filosa* (Fig. 4a). Only one protein with a cellulose-binding motif (CBM1) was found. The Plasmodiophorids contain several members of the cellulase containing GH5-hydrolase family. However, the GH5 hydrolases of the Plasmodiophorids that we could classify based on phylogeny belonged to the ceramidase GH5_12 and GH5_27 subfamilies^40^ (Supplementary Fig. S23).

The most prominent chitin-related CAZy domains in the protein models belonged to the GH18 chitinases, CE4 and CBM18 (Fig. 4b). *P. brassicae* contained 16 GH18 domain-containing chitinases and their evolutionary history shows whole gene duplications and internal GH18 domain duplication events (Supplementary Fig. S24). GH18 domains can be separated into three phylogenetic clusters A, B and C^41^. The Plasmodiophorid GH18 chitinase domains grouped to cluster A and the bacterial cluster C previously not reported to contain eukaryotic members. In the cluster A, the Plasmodiophorid GH18 s formed a specific subgroup (Pd1). These Pd1-chitinase genes were mainly expressed during spore formation and germination suggesting a specific role for those proteins in resting spore cell wall modification and degradation. The phylogeny also revealed a new oomycete-specific cluster of GH18 domains.

**Figure 5:**
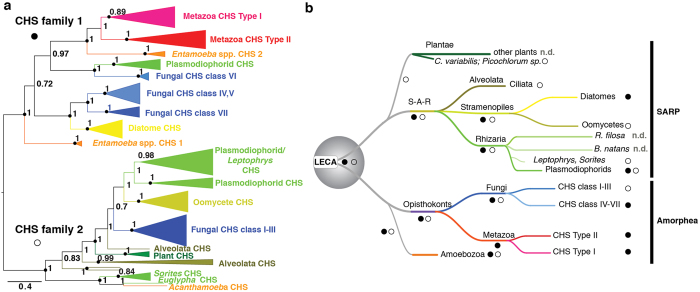
Interrelationships of eukaryotic chitin synthases (CHS) and model for CHS evolution in eukaryotes. (**a**) Unrooted Bayesian phylogenetic reconstruction of chitin synthase CHS domains (Chitin_synth_2; PF03142). Node support as represent posterior probabilities. Black circles indicate nodes supported of parallel ML analysis (RAxML, LG model, 500 replicates) with a bootstrap >70. The detailed tree is shown in Supplementary Fig. S25, and proteins used are listed in Supplementary Dataset 1. (**b**) A proposed model for the evolution of CHS in the eukaryotic kingdom based on current available data.

Recognition of microbial cell walls including chitin and chitin degradation products can be recognized by plants and trigger defense responses^12^. In a microarray assay^27^ the plant receptor genes for this type of recognition were suppressed after *P. brassicae* infection (Supplementary Table S10). The CBM18 domain and the LysM harboring CBM50 domain are known to bind chitin. Plants contain LysM domains in receptors involved in microbe recognition. Fungi can escape host recognition via LysM effectors, proteins that consist of a signal peptide and multiple LysMs, but no catalytic domain^12^. In the datasets of the two Plasmodiophorids only *P. brassicae* contained one protein with a single CBM50 domain. However, the CBM18 domain, which is reduced in biotrophic fungal lineages^39^,^42^ and absent in *B. natans* and *R. filosa* and plant pathogenic oomycetes, is frequent in both Plasmodiophorids (Fig. 5b). Similarly CBM18-domain proteins are enriched in the aquatic fungus *Batrachochytrium dendrobatadis*, and protect the fungus from breakdown by chitinases^43^. However, if the CBM18 proteins have similar functions in the Plasmodiophorids remains to be shown.

CE4-domain proteins can convert chitin into its derivate chitosan and were frequent in the Plasmodiophorids but absent in the other available Rhizaria genomes. Some CE4-domain proteins also included the chitin binding CBM18 domain, suggesting that these proteins bind to the Plasmodiophorid chitin in order to promote modification into chitosan, a weaker inducer of immune responses than chitin in many plants^12^.

### Plasmodiophorids contain two ancient chitin synthase (CHS) families

CHS are essential for the chitin synthesis. In *P. brassicae* 13 *CHS*-encoding genes and six in *S. subterranea* were identified. Whereas *B. natans* and *R. filosa* contained no predicted CHS, transcript data from *Leptophrys, Sorites* and *Euglypha* species include CHS domains, suggesting that chitin is also synthesized by Rhizarian outside the Plasmodiophorids. CHS are divided into two paralogous families, with family 1 represented by fungal CHS classes IV-VII, and family 2 by fungal CHS classes I–III^44^. We extended previous phylogenetic analyses of CHS by including CHS domains from Rhizarian, Stramenopiles, Alveolata and from green algae (*Chlorella variabilis, Picochlorum* species) (Supplementary Fig. S25). Plasmodiophorid harbor both CHS families. No Plasmodiophorid CHS acquired additional protein domains, unlike Metazoa and fungi CHS where fusion with myosin domains have occurred^45^. The protein domain structure and phylogeny of the Plasmodiophorid family 1 CHS are related to fungal class VI CHS. A family previously only detected in fungal species (Fig. 5a; Supplementary Figs. S25, S26). In contrast to recent analyses^45^, animal CHS regrouped with fungal CHS classes IV–VII in family 1, together with SARP CHS of Plasmodiophorids and diatoms. The CHS family 2 comprised fungal classes I-III, and CHS of oomycetes, plants, Aveolata and Rhizaria, including Plasmodiophorid in the SARP.

It was proposed that a branch of *β*-glycosyl-transferases developed into both CHS families after the plant kingdom split from the evolutionary line of Metazoa and fungi^44^. Our results revealed a different evolutionary scenario where the two lineages had already diverged in the last common ancestor of the SARP and Amorphea. This makes the absence of family 2 in Metazoa and family 1 in Plants and Alveolata all secondary losses (Fig. 5b). Family 2 was lost in the Metazoa concurrent with the divergence from Fungi in the Amorphea. In the SARP, Family 1 was lost in the Alveolata when they diverged from Rhizaria and Stramenopiles. Subsequently in the Stramenopiles, the loss of family 2 in diatoms, and family 1 in oomycetes occurred when those groups separated. In the Rhizaria both families are present the Plasmodiophorids. At least family 2 is also contained in *Sorites, Euglypha* and *Leptophrys*, whereas CHS appear to been lost in *R. filosa* and *B. natans*. The ancient origin of the CHS families suggests that synthesis of chitin or chitin-like structures has been a driving force in the evolution and diversification of early eukaryotes.

## Conclusion

We provide the *de novo* assembled, and extensively annotated genome sequence of *P. brassicae*, the first pathogenic genome in the eukaryotic supergroup Rhizaria. Our data identified new evolutionary histories, as shown for the *CHS* proteins. This emphasizes the importance of genome data from neglected eukaryotic kingdoms such as the Rhizaria^7^ for a better understanding of the evolutionary events in Eukaryotes. We give an important first comprehensive insight into the life cycle events of this uncultivable intracellular pathogen, including basic metabolism, secreted proteins, CAZymes, and phytohormones (Supplementary Fig. S27). Similar biotrophic features of Plasmodiophorids and phylogenetically unrelated eukaryotic biotrophic plant pathogens suggest a functional converging evolution. It remains challenging to elucidate the function of proteins in these uncultivatable protists. We provide Plasmodiophorid effector candidates which will be in focus of future studies to battle the agricultural important diseases caused by *P. brassicae* and other Plasmodiophorid pathogens.

## Methods

### DNA and RNA isolations

The *P. brassicae* single spore isolate e3^10^ was used in this work. Several centrifugation steps followed by surface sterilization, antibiotic treatments, achieved resting spore isolation and purification. Prior DNA extraction resting spores were treated with DNaseI to minimize host DNA contamination. DNA was extraction from purified *P. brassicae* resting spores using a modified CTAB method^46^ with an additional phenol:chloroform step after lysis or using the IIlustra phytopure DNA extraction kit. Prior the lysis step, the spores were incubated in 5 mg/ml Trichoderma lysing enzyme in 0.64 M KCl and 0.2 M CaCl_2_ for 12–16 h at room temperature to weaken the resting spore cell walls and grinded in liquid nitrogen using a mortar and pestle. DNA was further purified using the gDNA Zymo purification kit or magnetic beads. Total RNA was obtained from surface sterilized clubroots of *B. rapa, B. napus*, and *B. oleracea* var. *capitata*, 5 weeks post infection. Life-stage specific RNA was generated from germinating resting spores, maturing resting spores and plasmodia. *S. subterranea* RNA was extracted from potato root galls from potatoes grown for 2 months in natural infested soil. All RNA extractions were performed using the Spectrum™ Plant Total RNA Kit (Sigma-Aldrich).

### Genome sequencing and assembly

The *P. brassicae* genome was assembled by combining Roche 454 FLX and lllumina Hiseq 2100 sequencing data. A standard and a mate pair library with 3 kb inserts were constructed and analyzed by Macrogen, Korea using their 454 platform. Illumina libraries used included a 5 kb mate-pair library sequenced at the Bejing Genome Institute, Hong Kong, China, and a 200bp pair-end library sequenced at SciLife Laboratory, Stockholm, Sweden.

A first *de novo* genome draft of *P. brassicae* was assembled from 454 reads using Newbler v2.9^47^ with default settings. Homopolymer errors were corrected by the Nesoni pipeline v0.109. High quality Illumina genomic reads were used to scaffold the 454 assembly with SSPACE v2.2^48^ and to fill assembly gaps using GapFiller v1.11^49^. Scaffolds derived from contaminating host DNA were identified by BLASTN searches and their Illumina coverage and were removed from the assembly.

The *P. brassicae* genome assembly and the *S. subterranea* transcript data were checked for completeness by analysis of the single copy core eukaryotic orthologous genes using CEGMA 2.4^13^. Genome size estimation of *P. brassicae* was based on *k*-mer analysis of raw Illumina genomic reads. 17-mer coverage distribution was inferred using jellyfish^50^, using only reads that mapped to the *P. brassicae* assembly. The peak depth was at 478x. The peak of 17-mer frequency (M) in reads is correlated with the real sequencing depth (N), read length (L), and kmer length (K), their relations can be expressed in the formula: M = N * (L – K + 1)/L. The total sequence length (1447 Gb) divided by the real sequencing depth estimated a total genome size of 25.47 Mb.

### Transcriptome sequencing and filtering

RNA samples were sequenced with Illumina sequencing technology at SciLife Laboratory, Stockholm, Sweden and low-quality reads were removed. The transcriptome libraries of *P. brassicae* and *S. subterranea* were quality-filtered filtered using Condetri v.2.2^51^. *P. brassicae* libraries were mapped to the genome assembly using Tophat v2.0.9^52^ and analyzed using Cufflinks v 2.1.1^53^, with default settings and an expected intron length of 5 to 5 kb. *S. subterranea* transcripts were *de novo* assembled using Trinity^54^. Potato host transcripts were removed from the subsequent analyses, if the assembled transcripts had a BLASTN match to either the *Solanum tuberosum* genome^55^ or *A. thaliana* (TAIR 9, www.arabidopsis.org), and no BLASTN match to the *P. brassicae* assembly at an e-value cut-off <10^−9^. We removed transcripts with BLASTN matches to the potential contaminant Potato virus A/Y (11 transcripts), and transcripts masked as retroelement (1288 transcripts) or transposons (251 transcripts), detected using RepeatMasker ( http://www.repeatmasker.org), combined with the repeat database, RepBase^56^. Only transcripts coding for peptides of minimal length of 100 amino acids were subsequently analyzed.

### Gene content, annotation and expression

Repeats were annotated using RepeatModeler and RepeatMasker ( http://www.repeatmasker.org), combined with the repeat database, RepBase^56^ and the transposable element database of MAKER^57^. RepeatModeler was used to predict the novel repeat families, and these families were combined with RepBase to produce the final library, from which RepeatMasker was used to call the consensus repeat sequences. For subsequent gene annotations, the genome was masked from these repeat regions, except for simple repeats. We annotated the repeat masked *P. brassicae* genome to predict protein-coding genes with combined annotation methods of *ab initio* gene annotation, homology-based gene prediction and transcriptome information using MAKER^57^. All *ab initio* predictors in the MAKER pipeline, except GeneMark-ES, were initially trained with the CEGMA set and iterated retrained. The strand-specific RNA-sequencing reads of *P. brassicae* mapped to the assembly were used as expressed sequence tag (ESTs) evidence. The *P.brassicae* ESTs, tBLASTX hits (e-value<10^−10^) to the UniProt/Swissprot database, to *B. natans*^8^ and *R. filosa*^9^ and to Rhizarian ESTs from the NCBI database, were combined to generate an protein-coding gene set using Maker. All predicted gene models were if necessary manually adjusted using Apollo^58^. Gene models with insufficient evidence from either transcript coverage or BLASTP searches were removed. For protein family classification analysis, we used Interproscan v5.44.0^59^ to query multiple protein sequence and protein domain databases (Pfam v27.0, SMART v6.2, Gene3D v3.5.0, PANTHER v8.1, Phobius v1.01, SUPERFAMILY v1.75, PRINTS v42.0, ProSiteProfiles v20.89, ProSitePatterns). Gene Ontology terms were assigned with Blast2GO v2.5^60^. Biological pathway information for the *P. brassicae* genome and *S. subterranea* transcriptome, was obtained using the KEGG Automatic Annotation Server (KAAS) ( www.genome.jp/tools/kaas/) with the non-organism specific option. Orthologous and close paralogous genes were identified using OrthoMCL v2.2^17^. RNA sequencing reads mapped to the genome by TopHat^52^ were used for *P. brassicae* gene expression analysis by calculating the expected fragments per kilobase of transcript per million fragments (FPKM) using Cufflinks v 2.1.1^53^. Gene expressions were visualized in heat maps as log10 transformed FPKM values and normalized by calculating *Z*-scores for each gene across all transcriptome libraries.

### Secretome and selection of effector candidates

Proteins with a secretion signal predicted using SignalP-4.0^61^ and no transmembrane domain predicted using TMHMM v2.0^62^ were considered to be secreted. We identified 416 predicted secreted proteins below 450 amino acids in length. Number of candidates was reduced to 300 proteins as only genes that were expressed with a minimum value of at least 10 FPKM in at least one transcriptome library were considered. The transcriptome from the germinating spores was the only sample of *P. brassicae* without host contact. As effectors should interact with the plant we only selected protein models which genes had FPKM log2 fold change >5 in at least one other transcriptome library compared to the germinating spore library. This reduced the number of effector candidates to 92 (Supplementary Table 9).

### Identification and analysis of carbohydrate-related proteins

Carbohydrate active enzymes (CAZymes) in the protein models of *P. brassicae* and the *S. subterranea* were predicted with dbCAN “DataBase for automated Carbohydrate-active enzyme Annotation” annotation pipeline^63^. The protein models for all other compared organism were analyzed equally.

### Cloning, recombinant expression and conjugate synthetase test of the PbGH3 gene

The *PbGH3* gene was cloned in the expression vector pGEX 4T3 to give a glutathione S-transferase (GST) fusion protein (GST::PbGH3). The GST::PbGH3 protein was recombinant expressed in *Escherichia coli* and the protein purified. Quality and quantity of the eluted fusion protein were verified using 12% SDS-PAGE gel electrophoresis. As controls *E. coli* cells transformed with empty vector were used. Conjugation activity tests were performed as previously described^27^,^64^.

## Additional Information

**Accession Codes**: Data retrieved in this study are deposited at European Nucleotide Archive (ENA) under the projects PRJEB8376 (Accessions CDSF01000001-CDSF01000165) for the P. brassicae genomic data and PRJEB9159 (Accession HACM01000001-HACM01012732) for the S. subterranea transcriptome data.

**How to cite this article**: Schwelm, A. *et al.* The *Plasmodiophora brassicae* genome reveals insights in its life cycle and ancestry of chitin synthases. *Sci. Rep.* **5**, 11153; doi: 10.1038/srep11153 (2015).

## Supplementary Material

Supplementary Information

Supplementary Dataset 1

Supplementary Dataset 2

## Figures and Tables

**Figure 1:**
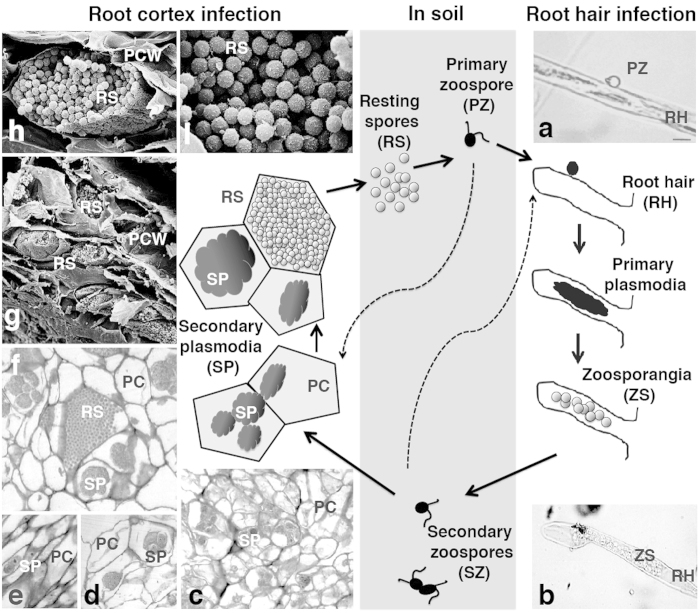
The life cycle of *Plasmodiophora brassicae*. A biflagellate primary zoospore (PZ) is released from each resting spore (RS). When the PZ reaches the surface of a root hair (RH) (**a**), it penetrates the cell wall and forms primary plasmodia in the root hairs. After a number of nuclear divisions the plasmodia cleaves into zoosporangia (ZS), developing and releasing secondary zoospores (SZ) (**b**). Occasional fusion of SZ has been suggested. PZ or SZ can penetrate root cortex cells, in which the pathogen develops into secondary plasmodia (SP) (**c**–**f**). Proliferation of SP is associated with cellular hypertrophy, causing a swollen root or club phenotype (Supplementary Fig. S1). After a number of nuclear divisions, the secondary plasmodia develop into multinuclear plasmodia and finally into resting spores (**f**–**i**), which are released into the surrounding soil when plants decay. RS = Resting spore, PZ = Primary zoospore, RH = root hair, ZS = Zoosporangia, SZ = Secondary zoospore, SP = Secondary plasmodia. PC = Plant cell, PCW = Plant cell wall
