# Supplementary material for: The Plasmodiophora brassicae genome reveals insights in its life cycle and ancestry of chitin synthases
Source: Sci Rep. 2015 Jun 18;5:11153. doi: 10.1038/srep11153 (PMC4471660; doi:10.1038/srep11153)
Supplement: Supplementary Information [file srep11153-s1.pdf]

Supplementary Information

The *Plasmodiophora brassicae* genome reveals insights in its life cycle and ancestry of chitin synthases

Arne Schwelm, Johan Fogelqvist, Andrea Knaust, Sabine Jülke, Tua Lilja, German Bonilla-Rosso, Magnus Karlsson, Andrej Shevchenko, Vignesh Dhandapani, Su Ryun Choi, Hong Gi Kim, Ju Young Park, Yong Pyo Lim, Jutta Ludwig-Müller and Christina Dixelius

Supplementary Figures S1-S27

Supplementary Tables 1-10

Supplementary Notes

Supplementary References

Supplementary Figures

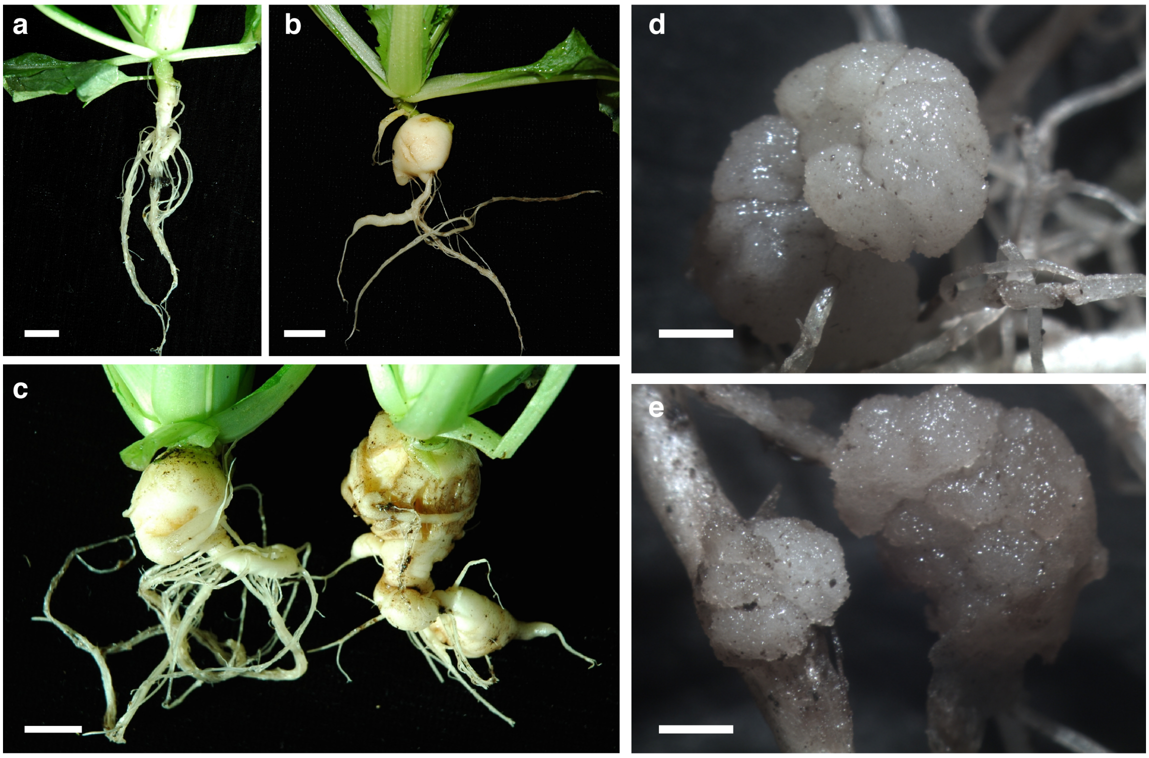

Supplementary Fig. S1. Plasmodiophorid galls on host plants. Clubroot symptoms of *P. brassicae* infected Chinese cabbage (*B. rapa* cv. Granaat) roots. (a) Healthy root. (b,c). Galls 5 weeks after infection on *B. rapa* cv. Granaat. (d,e) *S. subterranea* galls on potato roots. Size bar: a-c = 1 cm, d-e = 0.5 mm.

a

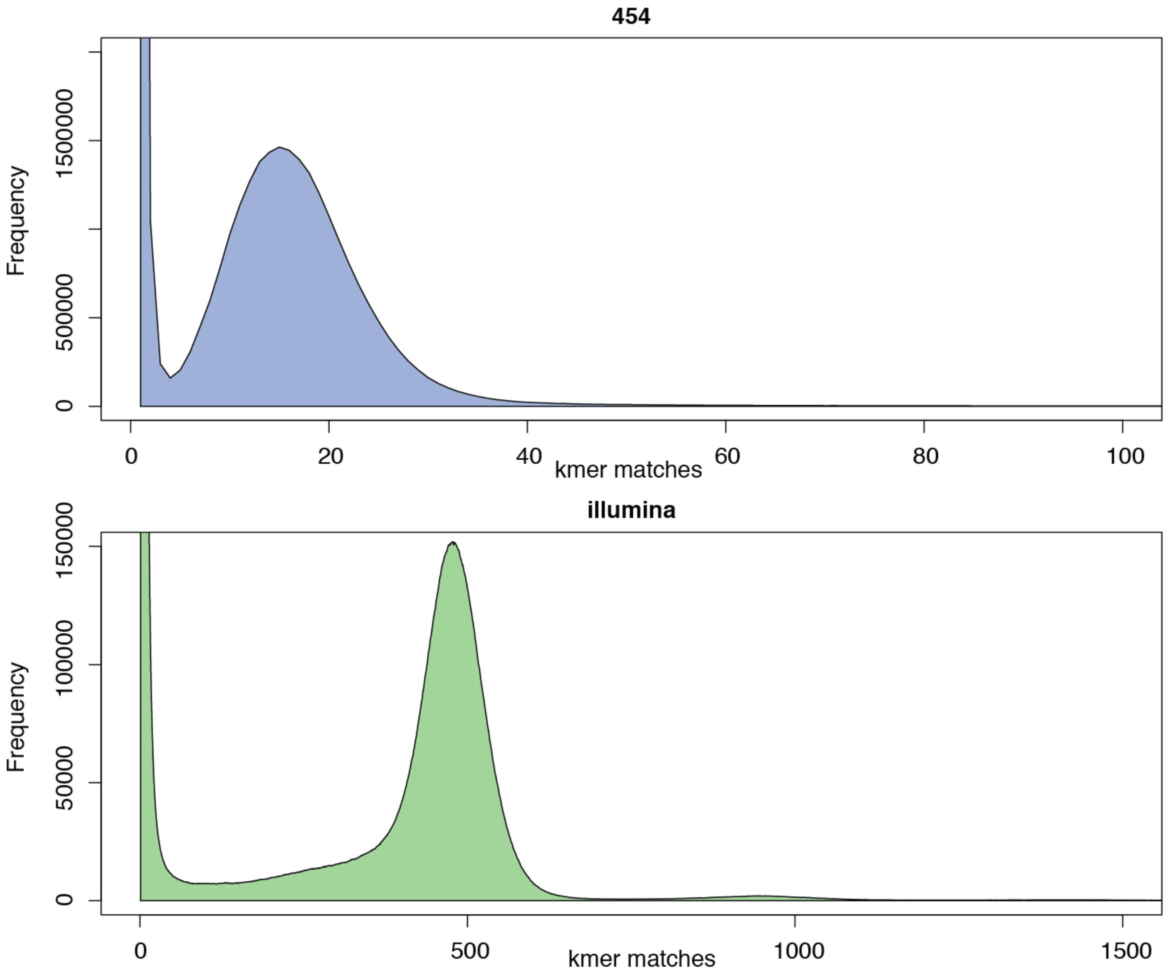

b

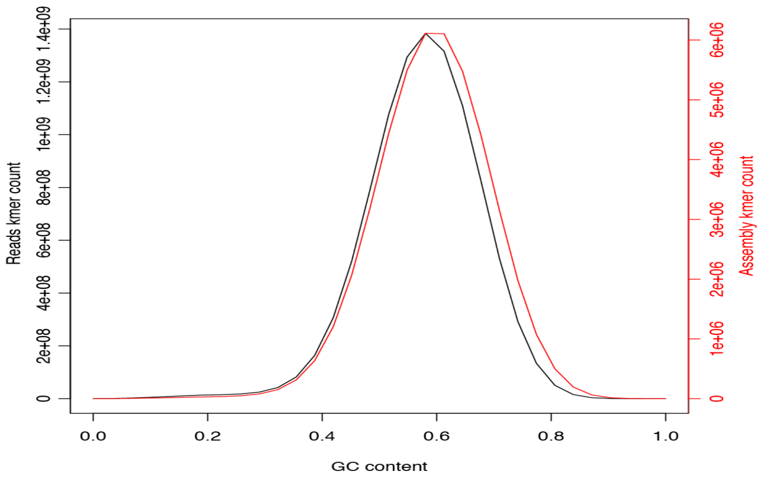

Supplementary Fig. S2. Genome coverage of the *P. brassicae* genome. (a) Distribution of 17-mer frequency in the raw 454 and Illumina genomic reads. (b) GC-content of assembly and reads.

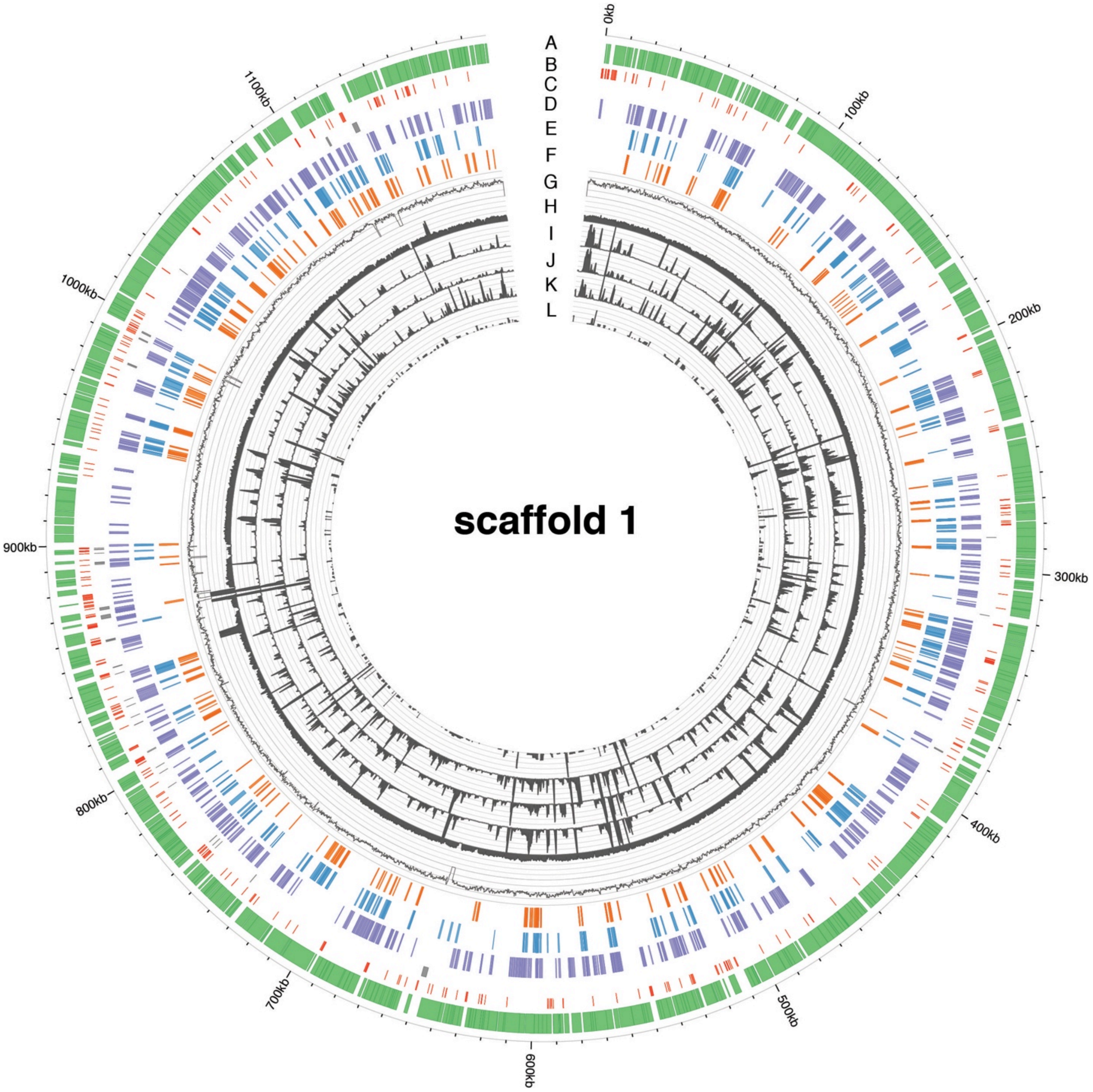

A
B
C
D
E
F
G
H
I
J
K
L
scaffold 1
0kb
100kb
200kb
300kb
400kb
500kb
600kb
700kb
800kb
900kb
1000kb
1100kb

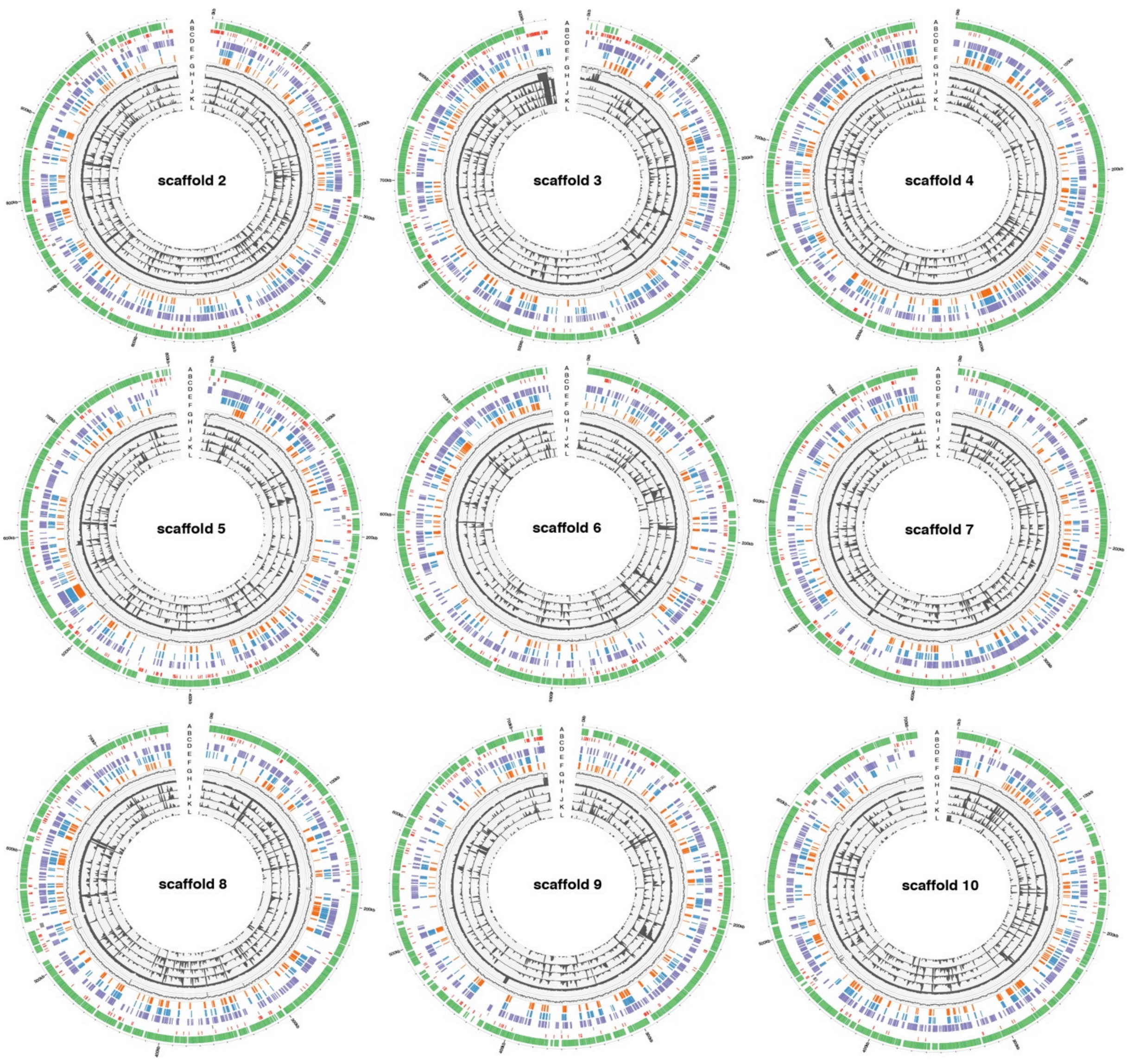

Supplementary Fig. S3. Circos circular visualization of the scaffolds 1-10 of the *P. brassicae* genome assembly. Track A denotes *P. brassicae* gene models (green), track B, repeated elements (red), and track C, sequence gaps (gray). tBLASTn hits of an e-value of $<10^{-10}$ to track D, *Bigellowiella natans* (blue), track E, *Reticulomyxae filosa* (orange) and track F, *Spongospora subterranea* (purple) are indicated. Track G shows guanosine and cytosine (GC) content based on a sliding window average of 200bp. Track H shows the Illumina read coverage on the scaffold (scale 0-650). Track I-L shows transcript coverage by FPKM. RNA-Seq reads from *P. brassicae*: I, germinating spores (scale 0–6000), J, maturing spores (scale 0-5000) and K, plasmodia (scale 0-10000). Track L shows RNA-Seq reads from *S. subterranea* (scale 0-1500). The gene sparse, highly covered and highly repetitive region at end of scaffold 3 represents the ribosomal genes.

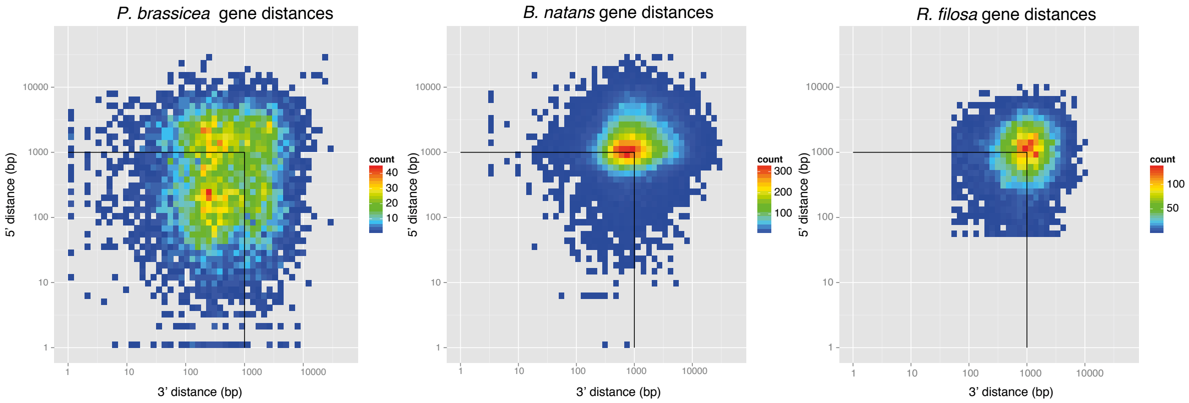

Supplementary Fig. S4. Gene densities of *P. brassicae, B. natans* and *R. filosa* genomes. Number of genes sorted by their 3' and 5' flanking intergenic distances. Genes with less than two flanking regions (i.e. genes at ends of scaffolds) were excluded. Color code indicates the numbers of genes found with the corresponding 3' and 5' end distances. Genes captured within the black lines represents genes with shorter intergenic distances than 1kb on 3' and 5' ends. Those represent 46.7% (4409 out of 9437) of *P. brassicae* genes and 20.5 % (4351 out of 21243) and 26.1% (2125 out of 8157 genes) of *B. natans* and *R. filosa* genes respectively.

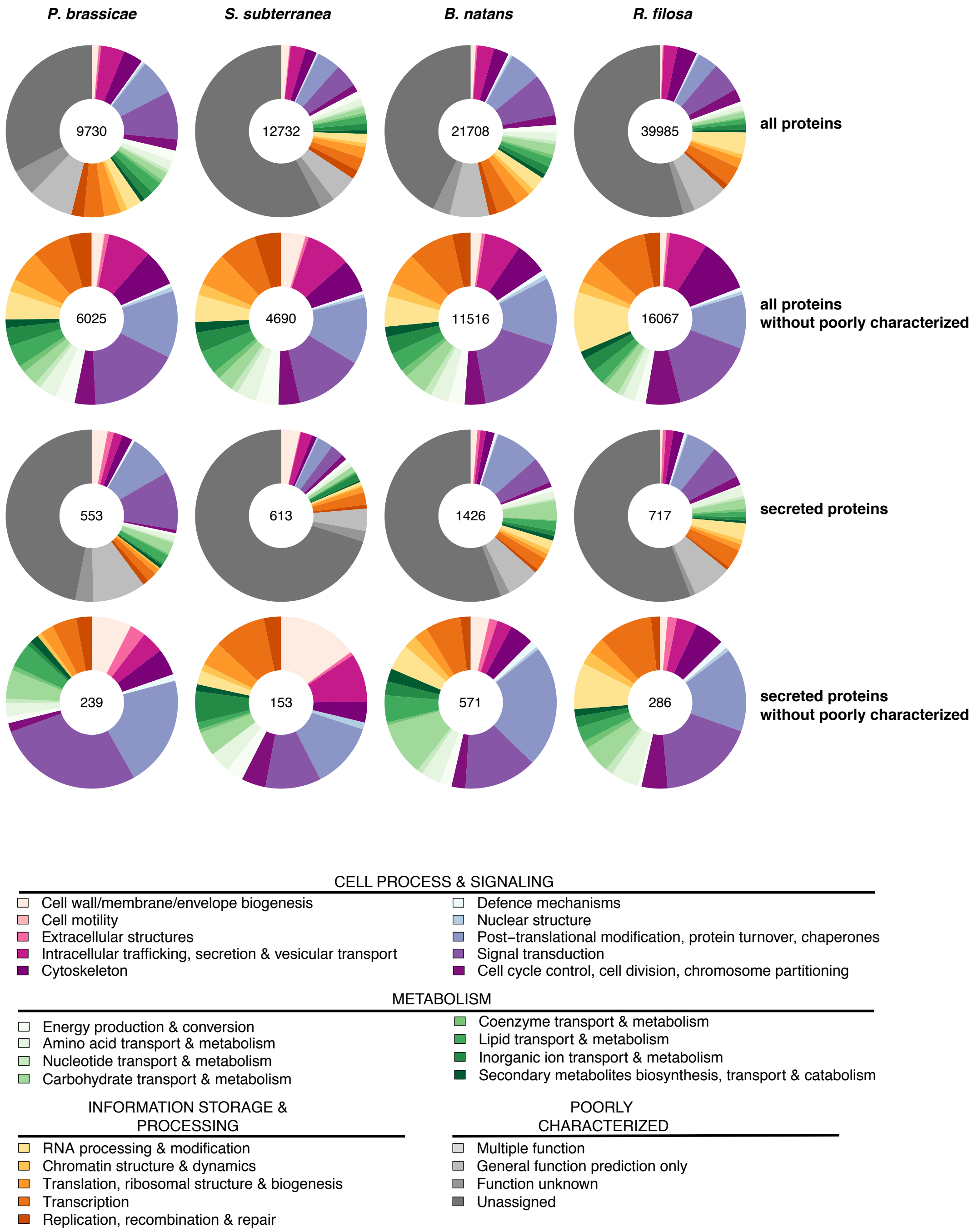

Supplementary Fig. S5. Comparison of KOG categories for the protein models of Rhizaria. Functional annotation according to KOG categories of the *P. brassicae, B. natans* and *R. filosa* genome and the *S. subterranea* transcriptome.

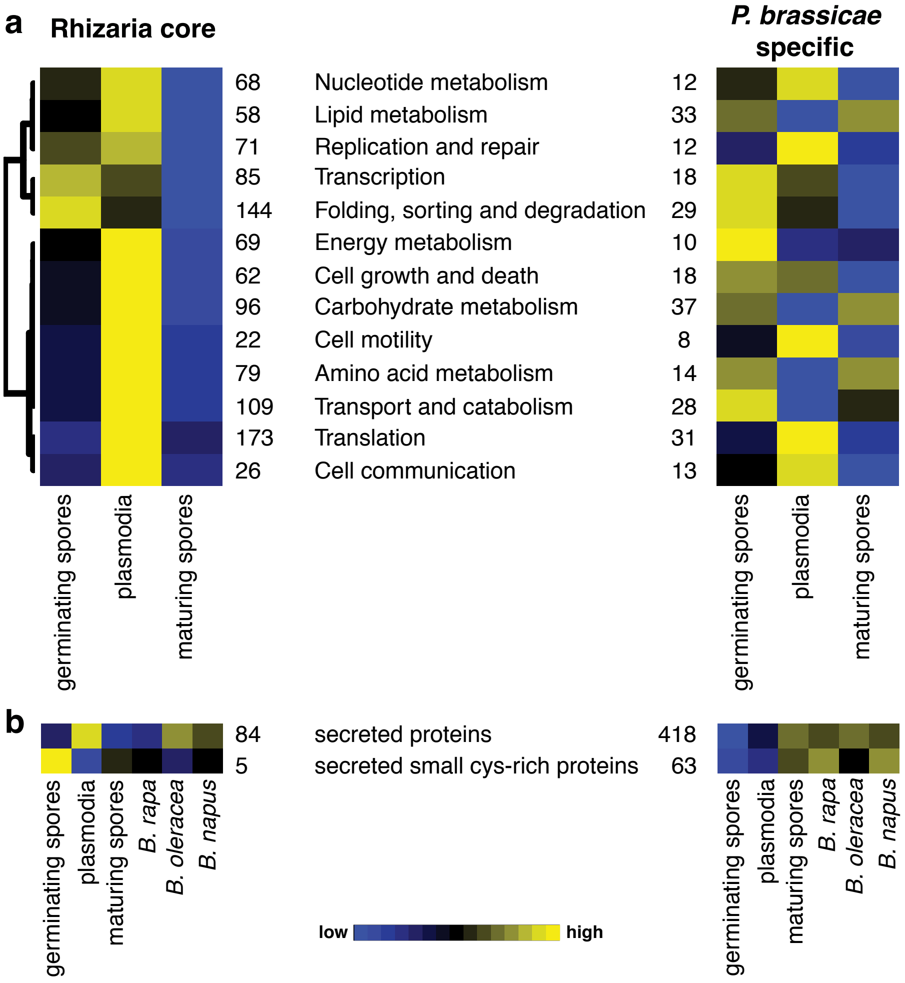

Supplementary Fig. S6. Gene expression of the Rhizaria core set and the *P. brassicae* specific genes. (a) Accumulative gene expression patterns of KEGG main categories. (b) Predicted secreted proteins for the Rhizaria core set. The *P. brassicae*-specific genes are determined by OrthoMCL-groups as shown in Fig. 2. Gene expression pattern (left) is visualized showing Z-scores for each gene within all transcriptome libraries. Numbers of genes for each set is given.

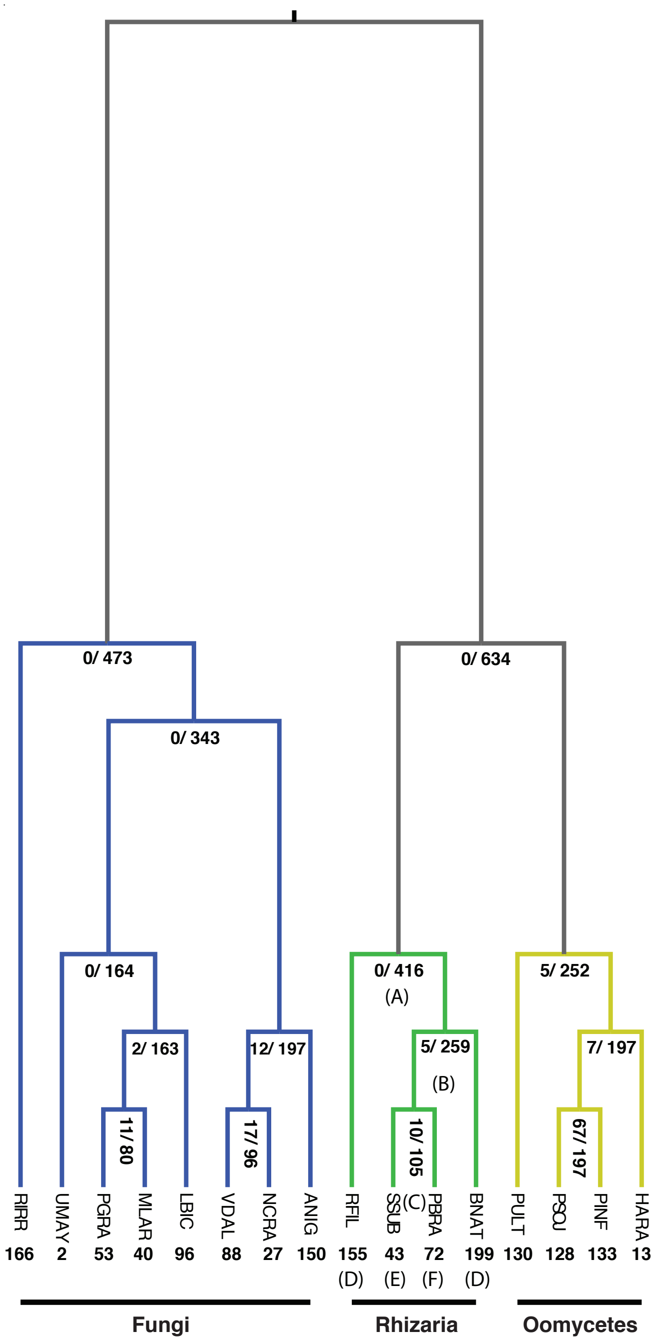

(A) Enriched pfam domains PBRA-SSUB-BNAT-RFIL
none

(B) Enriched pfam domains PBRA-SSUB-BNAT
PF00023.25 Ankyrin repeat
PF12796.2 Ankyrin repeats (3 copies)
PF13606.1 Ankyrin repeat
PF13637.1 Ankyrin repeats (many copies)
PF13857.1 Ankyrin repeats (many copies)

(C) Enriched pfam domains PBRA-SSUB
PF00023.25 Ankyrin repeat
PF00187.14 Chitin recognition protein
PF01522.16 Polysaccharide deacetylase
PF02140.13 Galactose binding lectin domain
PF08373.5 RAP domain
PF12796.2 Ankyrin repeats (3 copies)
PF13371.1 Tetratricopeptide repeat
PF13606.1 Ankyrin repeat
PF13637.1 Ankyrin repeats (many copies)
PF13857.1 Ankyrin repeats (many copies)

(D) Enriched pfam domains RFIL-BNAT but not PBRA-SSUB
PF00025.16 ADP-ribosylation factor family
PF00036.27 EF hand
PF00071.17 Ras family
PF00225.18 Kinesin motor domain
PF00610.16 Domain found in Dishevelled, Egl-10, and Pleckstrin (DEP)
PF00621.15 RhoGEF domain
PF00641.13 Zn-finger in Ran binding protein and others
PF01129.13 NAD:arginine ADP-ribosyltransferase
PF01344.20 Kelch motif
PF01697.22 Glycosyltransferase family 92
PF02181.18 Formin Homology 2 Domain
PF03016.10 Exostosin family
PF03176.10 MMPL family
PF03567.9 Sulfotransferase family
PF04564.10 U-box domain
PF08477.8 Miro-like protein
PF10591.4 Secreted protein acidic and rich in cysteine Ca binding region
PF13202.1 EF hand
PF13499.1 EF-hand domain pair
PF13704.1 Glycosyl transferase family 2
PF13833.1 EF-hand domain pair

(E+F) Enriched pfam domains in SSUB and PBRA see Suppl. Table 6

Supplementary Fig. S7. Pfam domain enrichment in the Rhizaria and Plasmodiohorids, compared to selected fungi and oomycete plant pathogens. Numbers at the tree nodes show the Pfam domains enriched in each clade (enriched in all species of the clade/total number of enriched Pfam domains) compared to all species in the tree. Numbers of Pfam domains enriched in each species are shown below the species abbreviation. Common enriched Pfam domains of clades A-C, and common enriched Pfam domains of *B. natans* and

R. filosa D are listed. Enriched Pfam domains of *S. subterranea* and *P. brassicae* are listed in Supplementary Table S6.

Phylogenetic tree was obtained from CEGMA core genes, with a single copy in at least 10 of the analyzed species. CEGMA genes were identified using BLAST (e-value<10^{-9}) against the CEGMA database (http://korflab.ucdavis.edu/datasets/cegma)[1]. A total of 128 genes were aligned using MUSCLE v3.8.31[2] and conserved regions were identified using GBLOCKS 0.91b[3]. The conserved regions were concatenated and the phylogeny constructed using MrBayes v3.4[4] assuming a strict molecular clock with uniform rate, using the gamma mutation model with invariable sites estimation. The Rhizarian, Oomycete and Fungi forced to occur as groups. MrBayes was run for 10^6 generations, using a burn-in of 2.5×10^5 generations.

Abbreviations for species used in the analysis: HARA = *Hyaloperonospora arabidopsidis*, PINF = *Phytophthora infestans*, PSOJ= *Phytophthora sojae*, PULT = *Pythium ultimatum*, BNAT = *Bigellowiella natans,* PBRA = *Plasmodiophora brassicae*, SSUB = *Spongospora subterranea*, RFL = *Reticulomyxae filosa*, ANIG= *Aspergillus nigra*, NCRA = *Neurospora crassa*, VDAL = *Verticillium dahliae,* LBIC = *Laccaria bicolor*, MLAR = *Melampsora larici-populina,* PGRA = *Puccinia graminis* f. sp. *tritici*, UMAY = *Ustilago maydis*, RIRR = *Rhizophagus irregularis.*

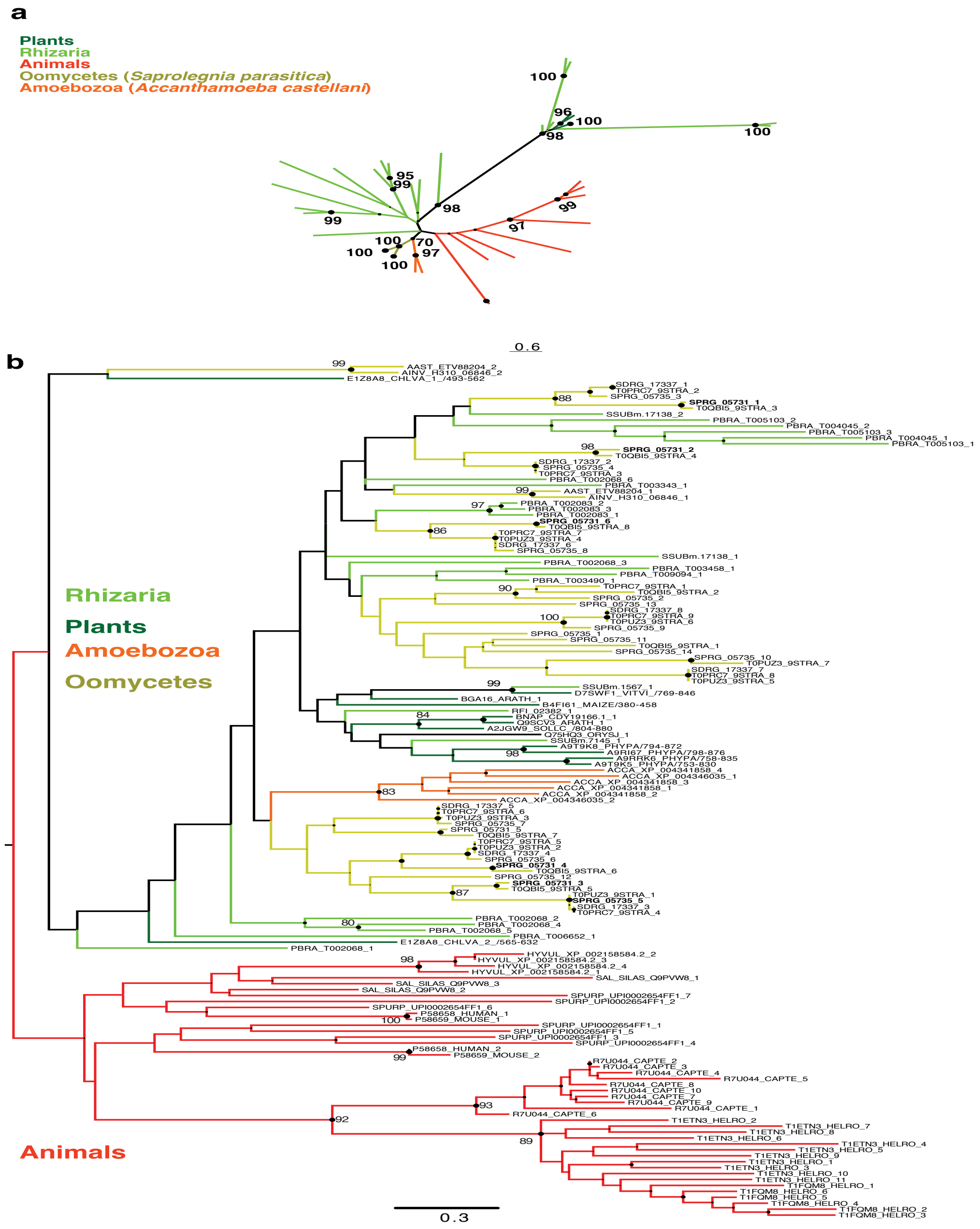

Supplementary Fig. S8. Phylogeny of galactose binding lectin (gal-lectin) domain containing proteins. (a) Phylogeny of whole length proteins that include a gal-lectin domain (PF02140) of plants, Rhizaria, Oomycetes, Amoebozoa and animals. (b) Phylogeny of the individual gal-lectin domains. The gal-lectin protein domains were identified by SMART (http://smart.embl.de/)[5]. Alignments were performed using T-Coffee[6]. Evolutionary analyses were conducted in MEGA6 using the Maximum Likelihood method based on the JTT matrix-based model. Numbers indicate interior branch bootstrap values as percentage, based on 1,000 replicates (values >80 are shown). Full information about abbreviations of proteins, accession number and species used in the analyses are deposit in Supplementary Dataset S1.

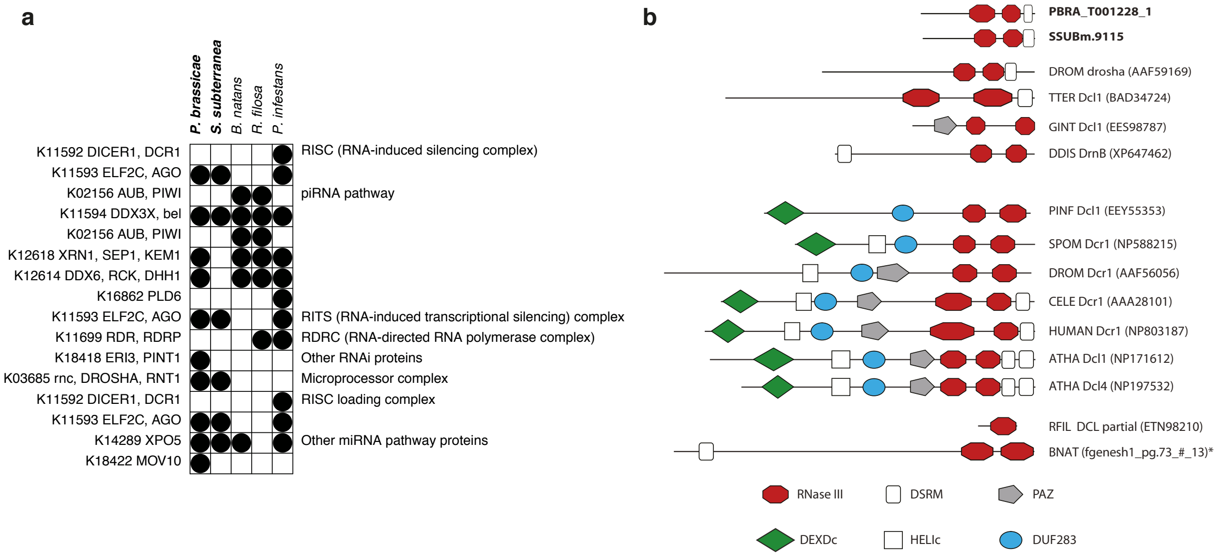

Supplementary Fig. S9. RNA silencing components and Dicer-like proteins. (a) Presence (black dot) and absence of RNA-silencing proteins in the Rhizaria compared to *P. infestans* according to the KEGG BRITE hierarchy. KEGG K-numbers and protein abbreviations are shown on the left, RNA silencing pathway categories on the right. (b) Domain organization of the Dicer/Drosha-like protein models of *P. brassicae* (PBRA_T001228_1) and *S. subterranea* (SSUBm.9115) compared to Drosha, Dicer-like proteins, and the RNAseIII domain proteins of *B. natans* and *R. filosa*. Protein accession numbers in parentheses.

Abbreviations: ATHA, *Arabidopsis thaliana*; BNAT, *B. natans*, CELE, *Caenorhabditis elegans*; DDIS, *Dictyostelium discoideum*; DROM, *Drosophila melanogaster*; GINT, *Giardia intestinalis*; HUMAN, *Homo sapiens*; PINF, *P. infestans*; RFIL, *R. filosa*; SPOM, *Schizosaccharomyces pombe*; TTER, *Thalassiosira pseudonana*.

Protein domains: RNase III, Ribonucelase III; DSRM, double-stranded RNA-binding; PAZ, Piwi, Argonaut and Zwille; DEXDc, DEAD-like helicase C-terminal; HELIc, Helicase C-terminal; DUF283, double strand RNA-binding.

* protein model from http://genome.jgi.doe.gov/Bigna1/Bigna1.home.html

a

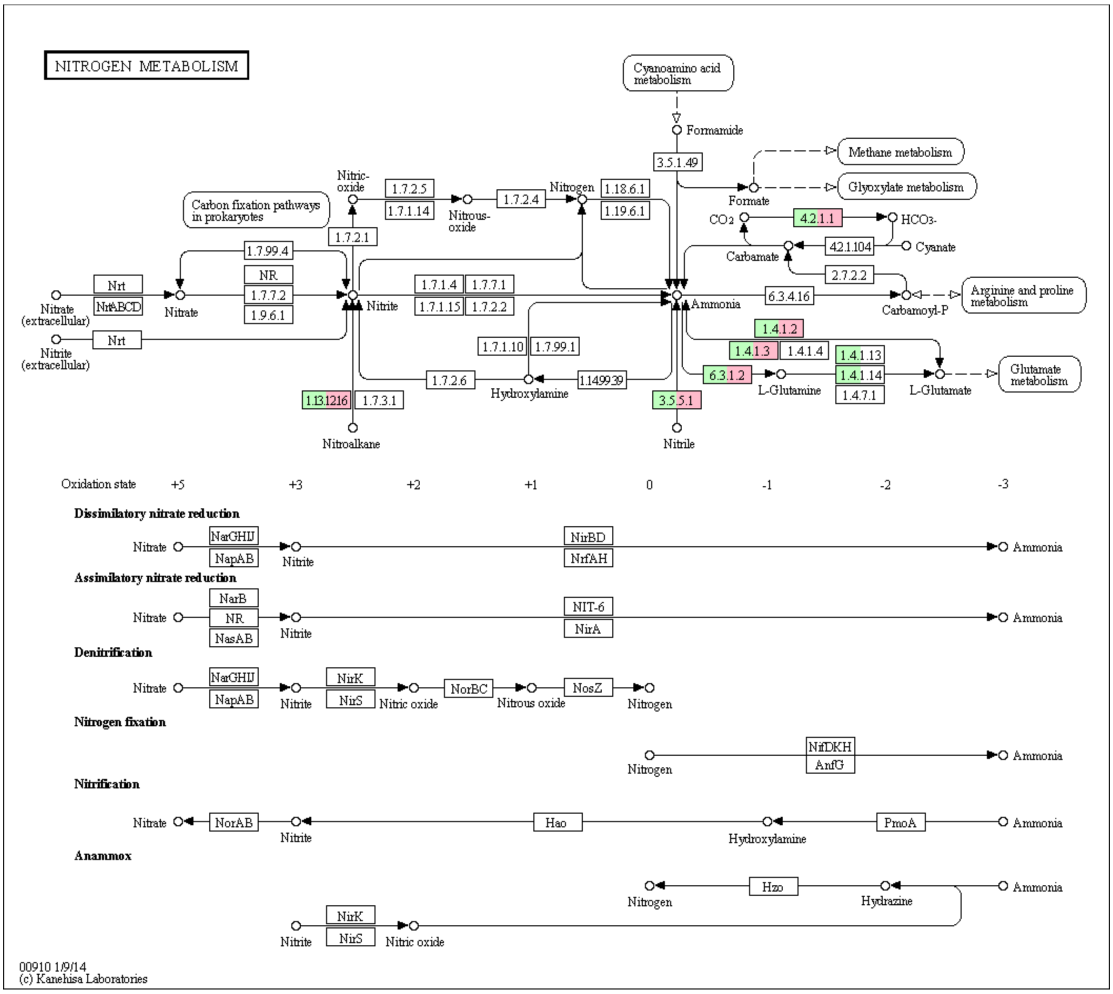

NITROGEN METABOLISM
Cyanoamino acid metabolism
Formamide
3.5.1.49
Methane metabolism
Glyoxylate metabolism
Formate
CO2
4.2.1.1
HCO3-
4.2.1.104
Cyanate
Carbamate
2.7.2.2
Arginine and proline metabolism
Carbamoyl-P
6.3.4.16
Carbon fixation pathways in prokaryotes
Nitric-oxide
1.7.2.5
1.7.1.14
Nitrous-oxide
1.7.2.4
Nitrogen
1.18.6.1
1.19.6.1
1.7.2.1
1.7.99.4
NR
1.7.7.2
1.9.6.1
Nrt
NrtABCD
Nitrate (extracellular)
Nitrate
Nitrite (extracellular)
Nitrite
1.7.1.4
1.7.7.1
1.7.1.15
1.7.2.2
Ammonia
1.4.1.2
1.4.1.3
1.4.1.4
1.4.1.13
1.4.1.14
1.4.7.1
6.3.1.2
L-Glutamine
L-Glutamate
Glutamate metabolism
1.7.1.10
1.7.99.1
1.7.2.6
Hydroxylamine
1.14.99.39
1.13.12.16
1.7.3.1
Nitroalkane
3.5.5.1
Nitrile
Oxidation state
+5
+3
+2
+1
0
-1
-2
-3
Dissimilatory nitrate reduction
NarGHIJ
NapAB
NirBD
NrfAH
Assimilatory nitrate reduction
NarB
NasAB
NIT-6
NirA
Denitrification
NirK
NirS
Nitric oxide
NorBC
Nitrous oxide
NosZ
Nitrogen fixation
NifDKH
AnfG
Nitrification
NorAB
Hao
PmoA
Anammox
Hzo
Hydrazine
00910 1/9/14
(c) Kanehisa Laboratories

b

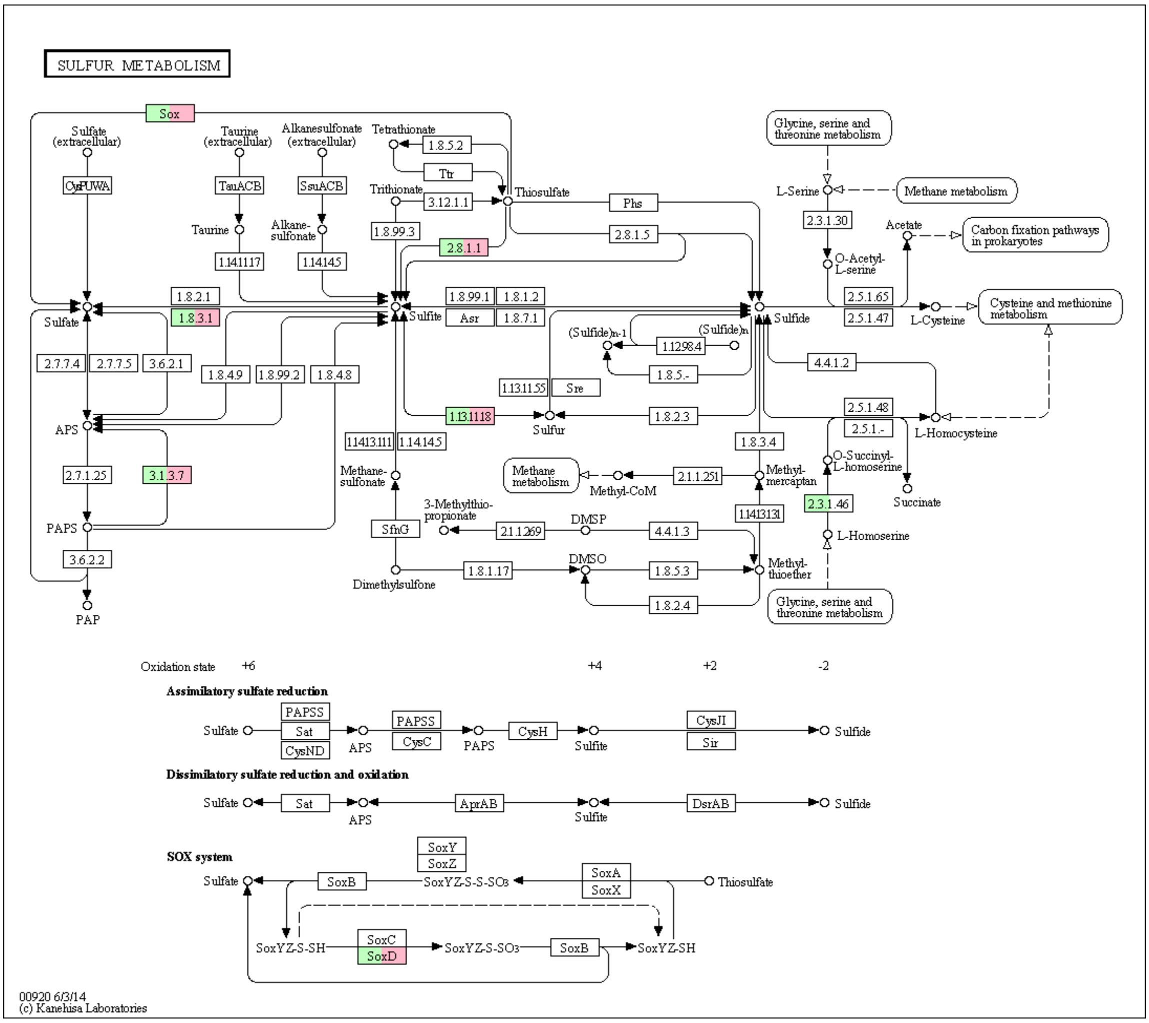

Supplementary Fig. S10. KEGG maps: on (a) nitrogen, and (b) sulfur metabolism of for *P. brassicae* (green) and *S. subterranea* (pink).

a

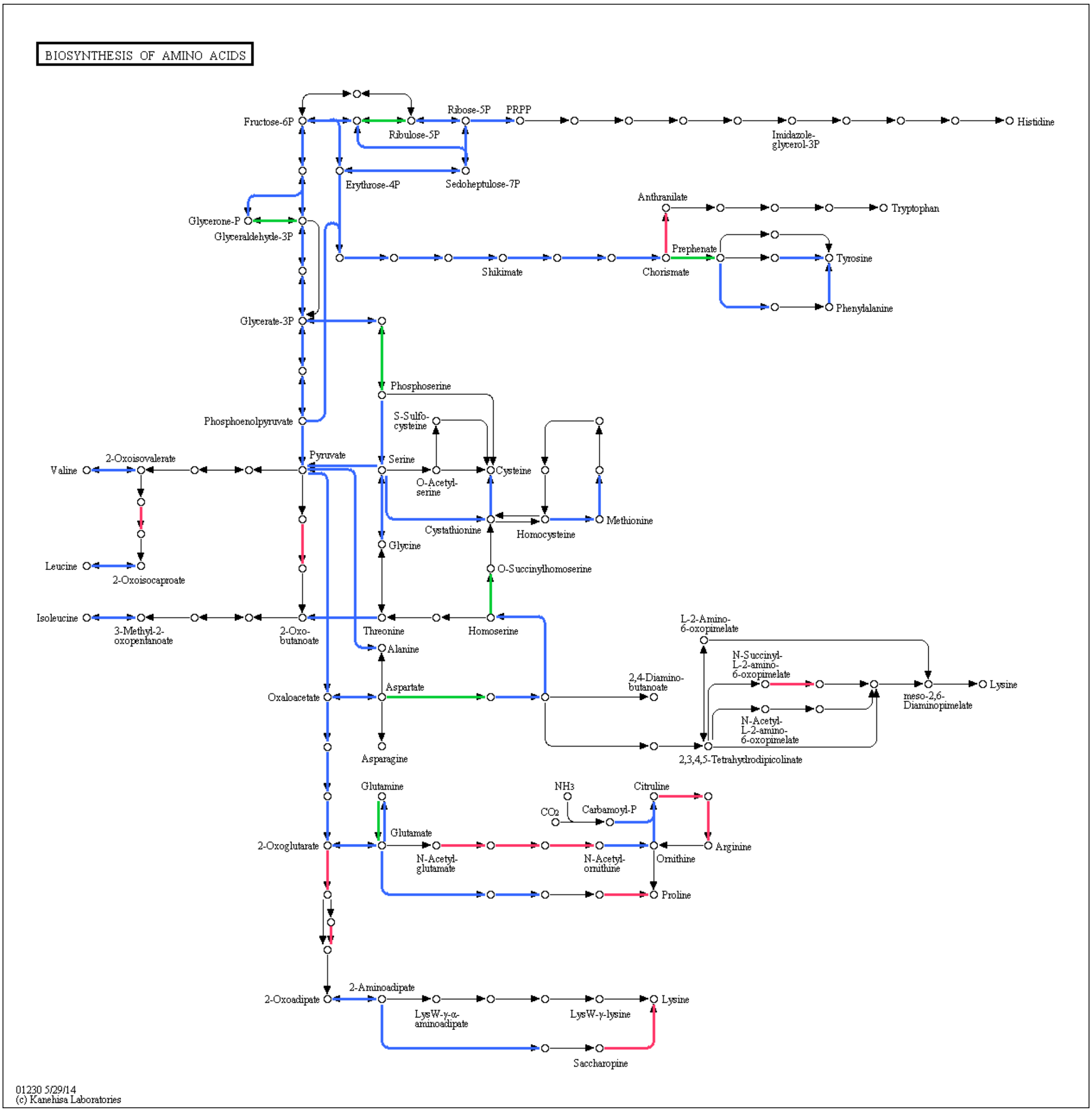

BIOSYNTHESIS OF AMINO ACIDS
Fructose-6P
Ribose-5P
PRPP
Ribulose-5P
Histidine
Imidazole-glycerol-3P
Erythrose-4P
Sedoheptulose-7P
Anthranilate
Tryptophan
Glycerone-P
Glyceraldehyde-3P
Prephenate
Tyrosine
Shikimate
Chorismate
Phenylalanine
Glycerate-3P
Phosphoserine
Phosphoenolpyruvate
S-Sulfo-cysteine
2-Oxoisovalerate
Pyruvate
Serine
Cysteine
Valine
O-Acetyl-serine
Methionine
Cystathionine
Homocysteine
Glycine
Leucine
2-Oxoisocaproate
O-Succinylhomoserine
Isoleucine
3-Methyl-2-oxopentanoate
2-Oxo-butanoate
Threonine
Homoserine
L-2-Amino-6-oxopimelate
Alanine
N-Succinyl-L-2-amino-6-oxopimelate
Aspartate
2,4-Diamino-butanoate
Lysine
Oxaloacetate
meso-2,6-Diaminopimelate
N-Acetyl-L-2-amino-6-oxopimelate
Asparagine
2,3,4,5-Tetrahydrodipicolinate
Glutamine
NH3
Citruline
CO2
Carbamoyl-P
Glutamate
2-Oxoglutarate
Arginine
N-Acetyl-glutamate
N-Acetyl-ornithine
Ornithine
Proline
2-Aminoadipate
2-Oxoadipate
Lysine
LysW-γ-α-aminoadipate
LysW-γ-lysine
Saccharopine
01230 5/29/14
(c) Kanehisa Laboratories

b

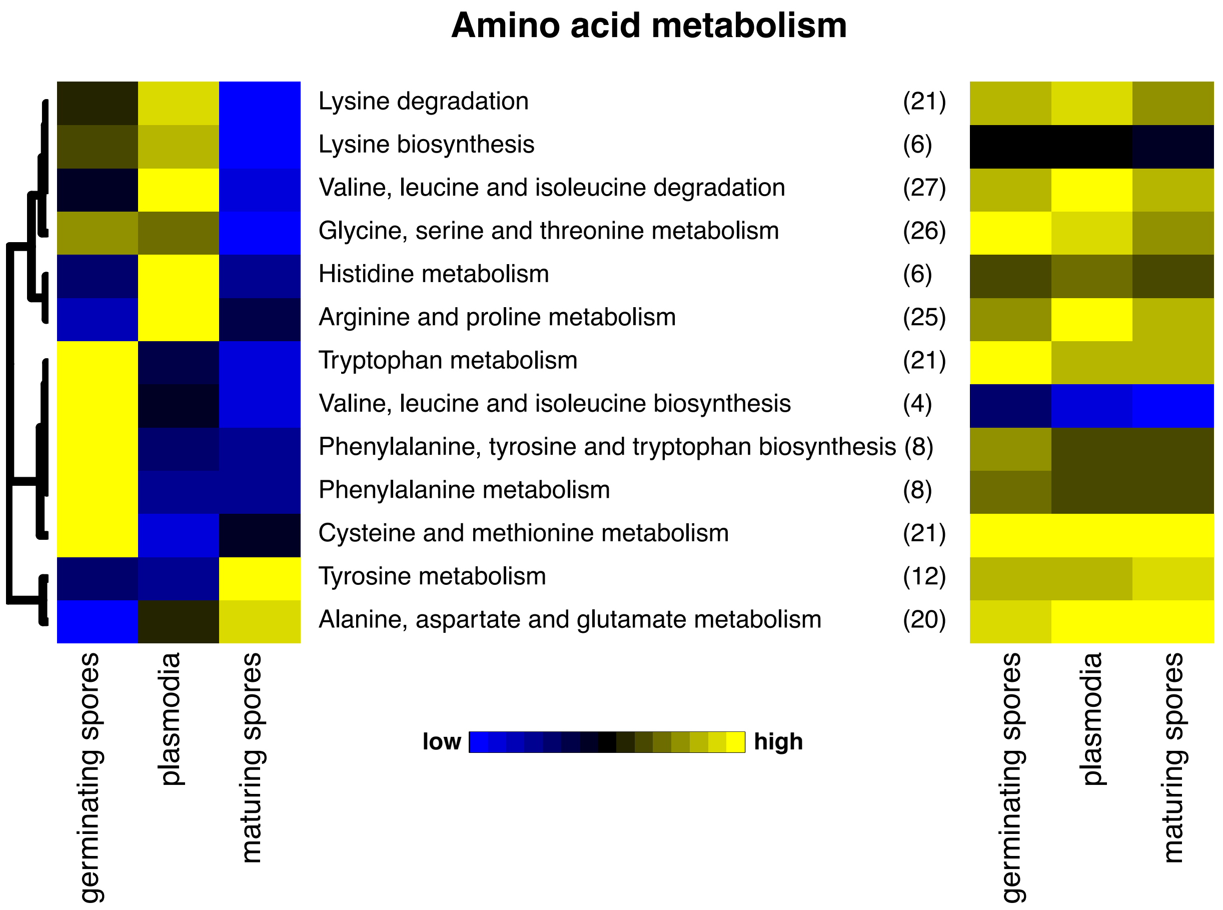

Supplementary Fig. S11. KEGG map and heat maps associated to amino acid metabolism. (a) KEGG map 01230 for amino acid metabolism of *P. brassicae* and *S. subterranea*. Common enzymatic steps identified by KEGG pathway reconstruction are indicated in blue. Steps missing in *P. brassicae* but present in *S. subterranea* are indicated in red and exclusive steps of *P. brassicae* are shown in green. (b) Gene expression of KEGG categories linked to map 01230. Gene expression pattern (left) is visualized showing Z-scores for each gene within all transcriptome libraries. Total gene expression (right) is visualized, showing log10 transformed FPKM values.

a

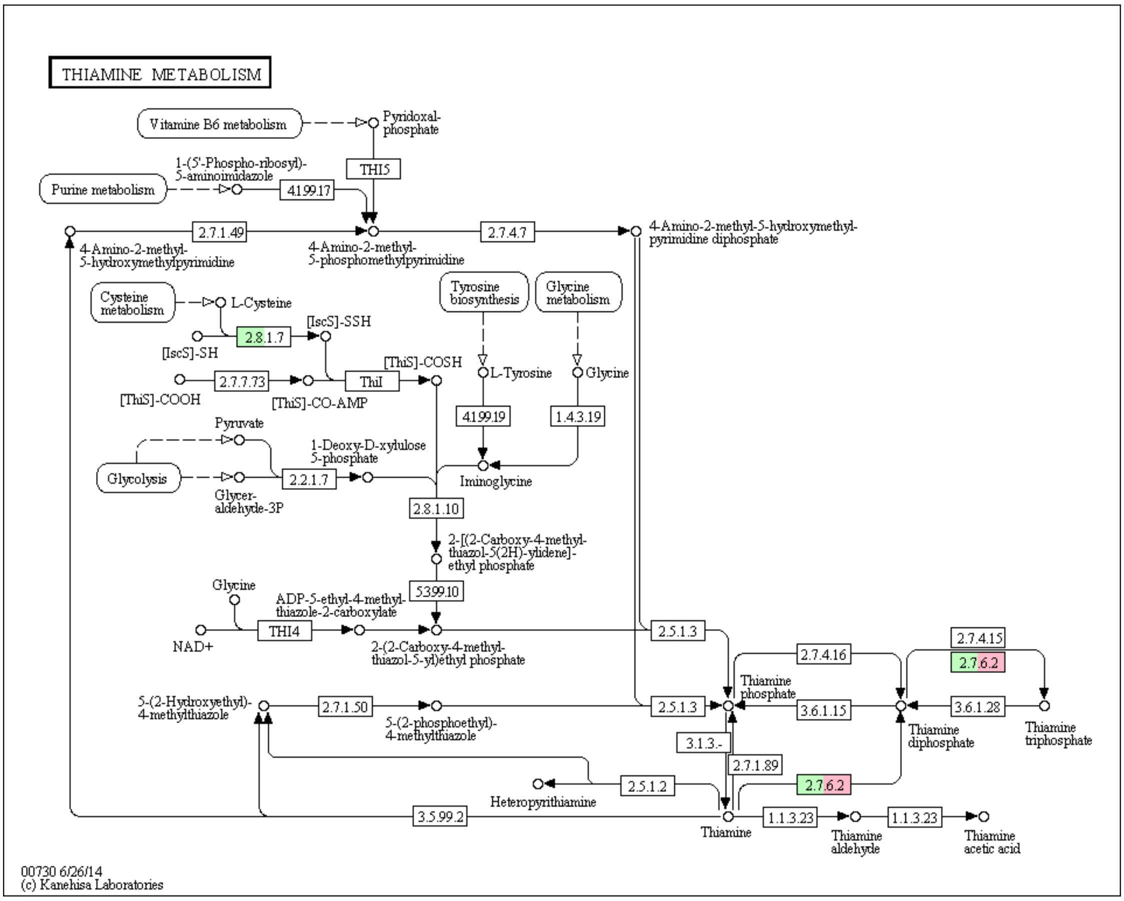
THIAMINE METABOLISM
Vitamine B6 metabolism
Pyridoxal-phosphate
THI5
1-(5'-Phospho-ribosyl)-5-aminoimidazole
Purine metabolism
4.1.99.17
2.7.1.49
2.7.4.7
4-Amino-2-methyl-5-hydroxymethylpyrimidine diphosphate
4-Amino-2-methyl-5-hydroxymethylpyrimidine
4-Amino-2-methyl-5-phosphomethylpyrimidine
Cysteine metabolism
L-Cysteine
Tyrosine biosynthesis
Glycine metabolism
[IscS]-SSH
2.8.1.7
[IscS]-SH
[ThiS]-COSH
2.7.7.73
ThiI
L-Tyrosine
Glycine
[ThiS]-COOH
[ThiS]-CO-AMP
4.1.99.19
1.4.3.19
Pyruvate
1-Deoxy-D-xylulose 5-phosphate
Glycolysis
2.2.1.7
Iminoglycine
Glyceraldehyde-3P
2.8.1.10
2-[(2-Carboxy-4-methylthiazol-5(2H)-ylidene]-ethyl phosphate
Glycine
ADP-5-ethyl-4-methylthiazole-2-carboxylate
5.3.99.10
NAD+
THI4
2.5.1.3
2-(2-Carboxy-4-methylthiazol-5-yl)ethyl phosphate
2.7.4.15
2.7.4.16
2.7.6.2
Thiamine phosphate
5-(2-Hydroxyethyl)-4-methylthiazole
2.7.1.50
2.5.1.3
3.6.1.15
3.6.1.28
5-(2-phosphoethyl)-4-methylthiazole
Thiamine diphosphate
Thiamine triphosphate
3.1.3.-
2.7.1.89
2.5.1.2
2.7.6.2
Heteropyrithiamine
3.5.99.2
1.1.3.23
1.1.3.23
Thiamine
Thiamine aldehyde
Thiamine acetic acid
00730 6/26/14
(c) Kanehisa Laboratories

b

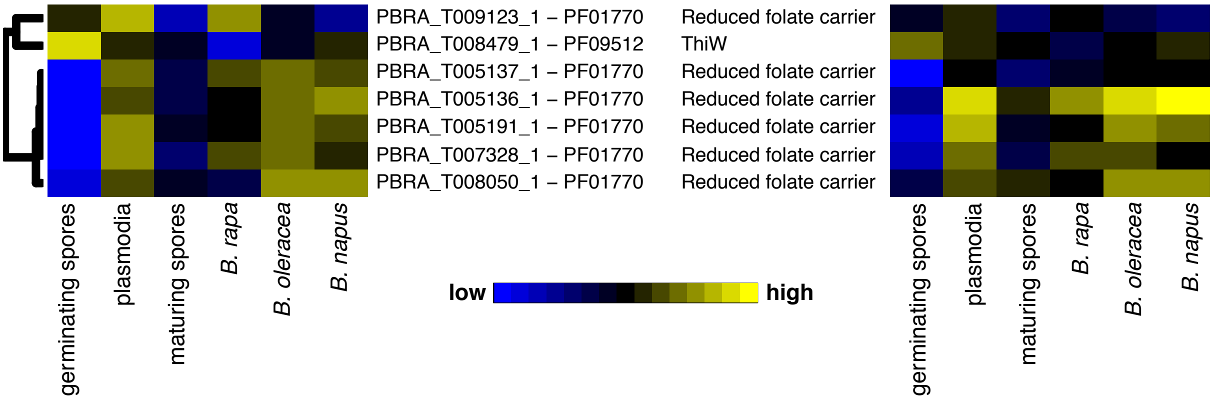
PBRA_T009123_1 – PF01770 Reduced folate carrier
PBRA_T008479_1 – PF09512 ThiW
PBRA_T005137_1 – PF01770 Reduced folate carrier
PBRA_T005136_1 – PF01770 Reduced folate carrier
PBRA_T005191_1 – PF01770 Reduced folate carrier
PBRA_T007328_1 – PF01770 Reduced folate carrier
PBRA_T008050_1 – PF01770 Reduced folate carrier
germinating spores
plasmodia
maturing spores
B. rapa
B. oleracea
B. napus
low
high
germinating spores
plasmodia
maturing spores
B. rapa
B. oleracea
B. napus

Supplementary Fig. S12. KEGG map for thiamine biosynthesis and gene expression of putative thiamine transporters. (a) KEGG map for thiamine biosynthesis with identified protein for *P. brassicae* (green) and *S. subterranea* (pink). (b) Expression pattern of potential thiamine transporter in *P. brassicae*. Gene expression pattern (left) is visualized showing Z-scores for each gene within all transcriptome libraries. Total gene expression (right) is visualized, showing log10 transformed FPKM values.

a

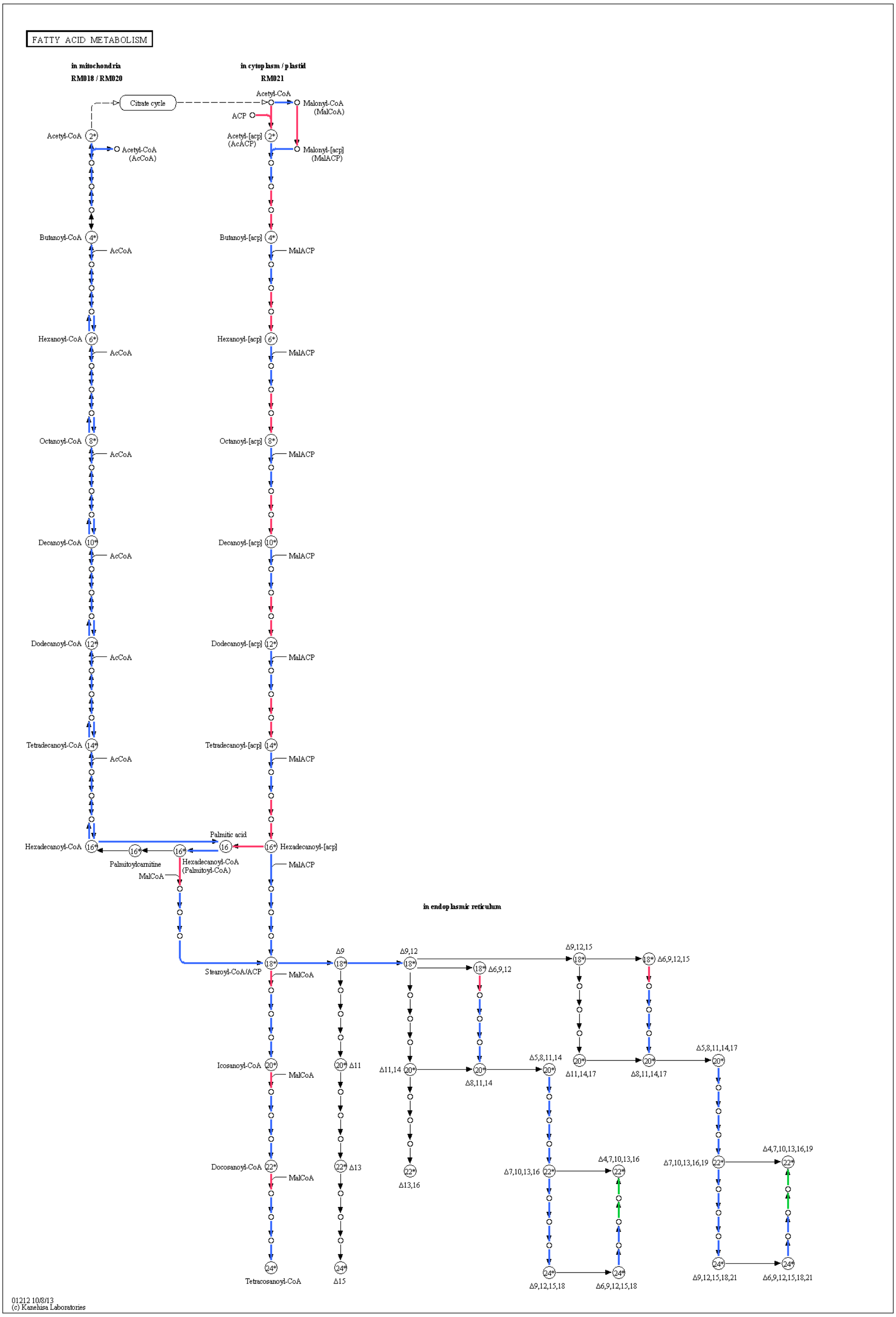
FATTY ACID METABOLISM
in mitochondria
RM018 / RM020
in cytoplasm / plastid
RM021
in endoplasmic reticulum
Citrate cycle
Acetyl-CoA
Malonyl-CoA
(MalCoA)
ACP
Acetyl-[acp]
(AcACP)
Malonyl-[acp]
(MalACP)
Acetyl-CoA
(AcCoA)
Butanoyl-CoA
Butanoyl-[acp]
Hexanoyl-CoA
Hexanoyl-[acp]
Octanoyl-CoA
Octanoyl-[acp]
Decanoyl-CoA
Decanoyl-[acp]
Dodecanoyl-CoA
Dodecanoyl-[acp]
Tetradecanoyl-CoA
Tetradecanoyl-[acp]
Hexadecanoyl-CoA
Hexadecanoyl-[acp]
AcCoA
MalACP
Palmitic acid
Palmitoylcarnitine
Hexadecanoyl-CoA
(Palmitoyl-CoA)
MalCoA
Stearoyl-CoA/ACP
Icosanoyl-CoA
Docosanoyl-CoA
Tetracosanoyl-CoA
Δ9
Δ9,12
Δ9,12,15
Δ6,9,12
Δ6,9,12,15
Δ11
Δ11,14
Δ8,11,14
Δ5,8,11,14
Δ11,14,17
Δ8,11,14,17
Δ5,8,11,14,17
Δ13
Δ13,16
Δ7,10,13,16
Δ4,7,10,13,16
Δ7,10,13,16,19
Δ4,7,10,13,16,19
Δ15
Δ9,12,15,18
Δ6,9,12,15,18
Δ9,12,15,18,21
Δ6,9,12,15,18,21
01212 10/8/13
(c) Kanehisa Laboratories

b

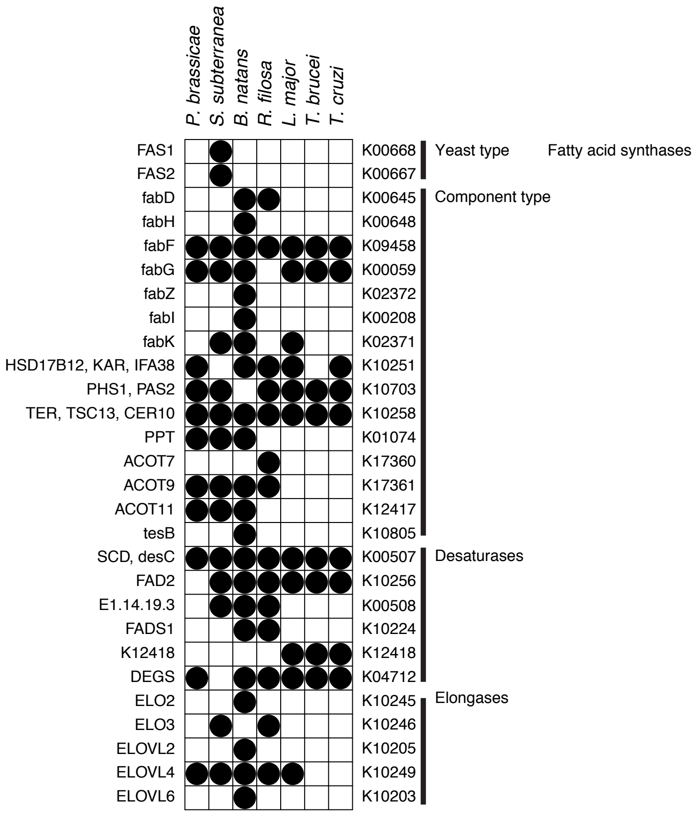

Supplementary Fig. S13. KEGG map for fatty acid synthesis associated gene content. (a) KEGG map of fatty acid biosynthesis for *P. brassicae* and *S. subterranea*. Common enzymatic steps identified by KEGG pathway reconstruction are indicated in blue. Steps missing in *P. brassicae* but present in *S. subterranea* are indicated in red and exclusive steps of *P. brassicae* are shown in green. (b) Fatty acid synthesis gene content according to the KEGG BRITE hierarchy. KEGG K-numbers and functional categories are shown on the right and protein abbreviations are shown on the left. A black dot marks the presence of a gene of the corresponding KEGG K-number. Rhizaria species are compared to the kinetoplasts *Trichoderma brucei*, *T. cruzi* and *Leishmania major*.

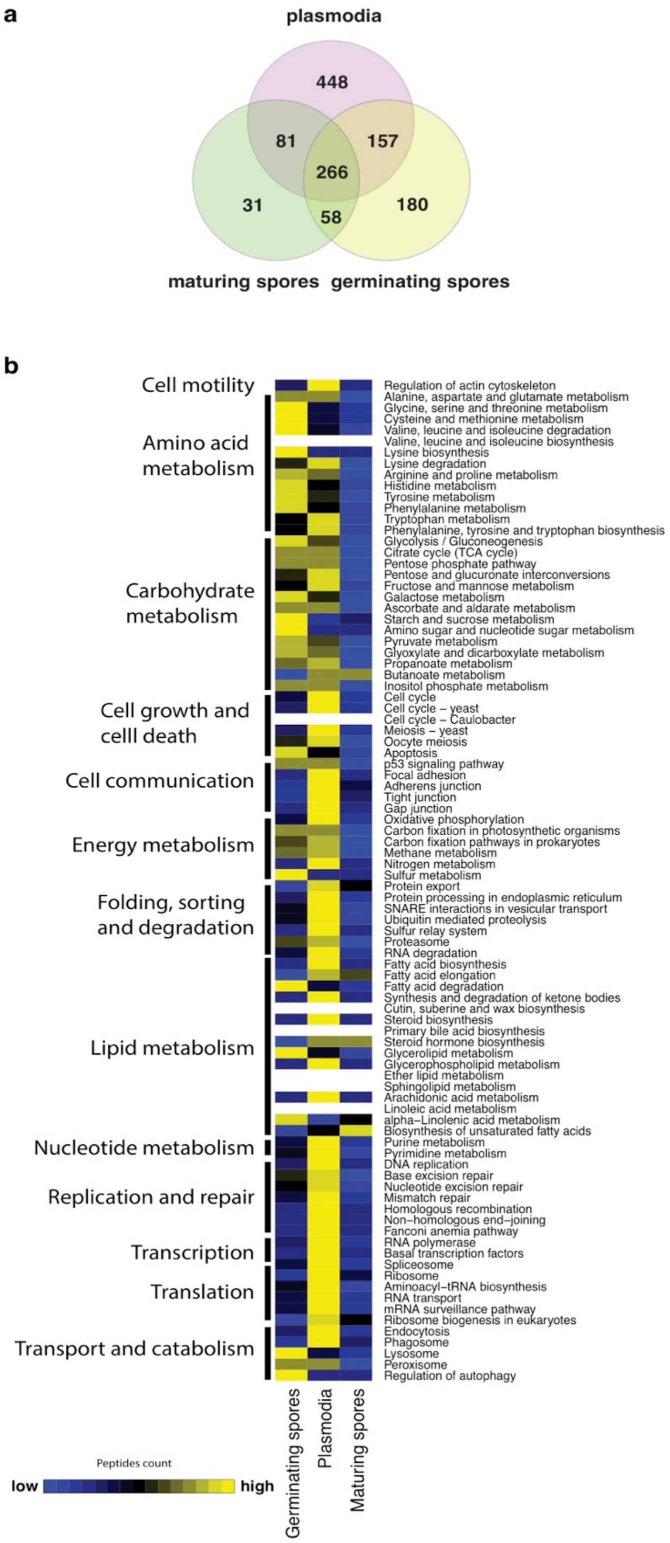

Supplementary Fig. S14. *P. brassicae* proteins in the three life-stages and their corresponding relative abundance. (a) Venn diagram of 1233 proteins identified by mass spectrometry and their distribution in the in three developmental stages. (b) Pattern of detected peptides sorted according to their KEGG categories. A full list of the peptide counts is deposited in Supplementary Dataset 2.

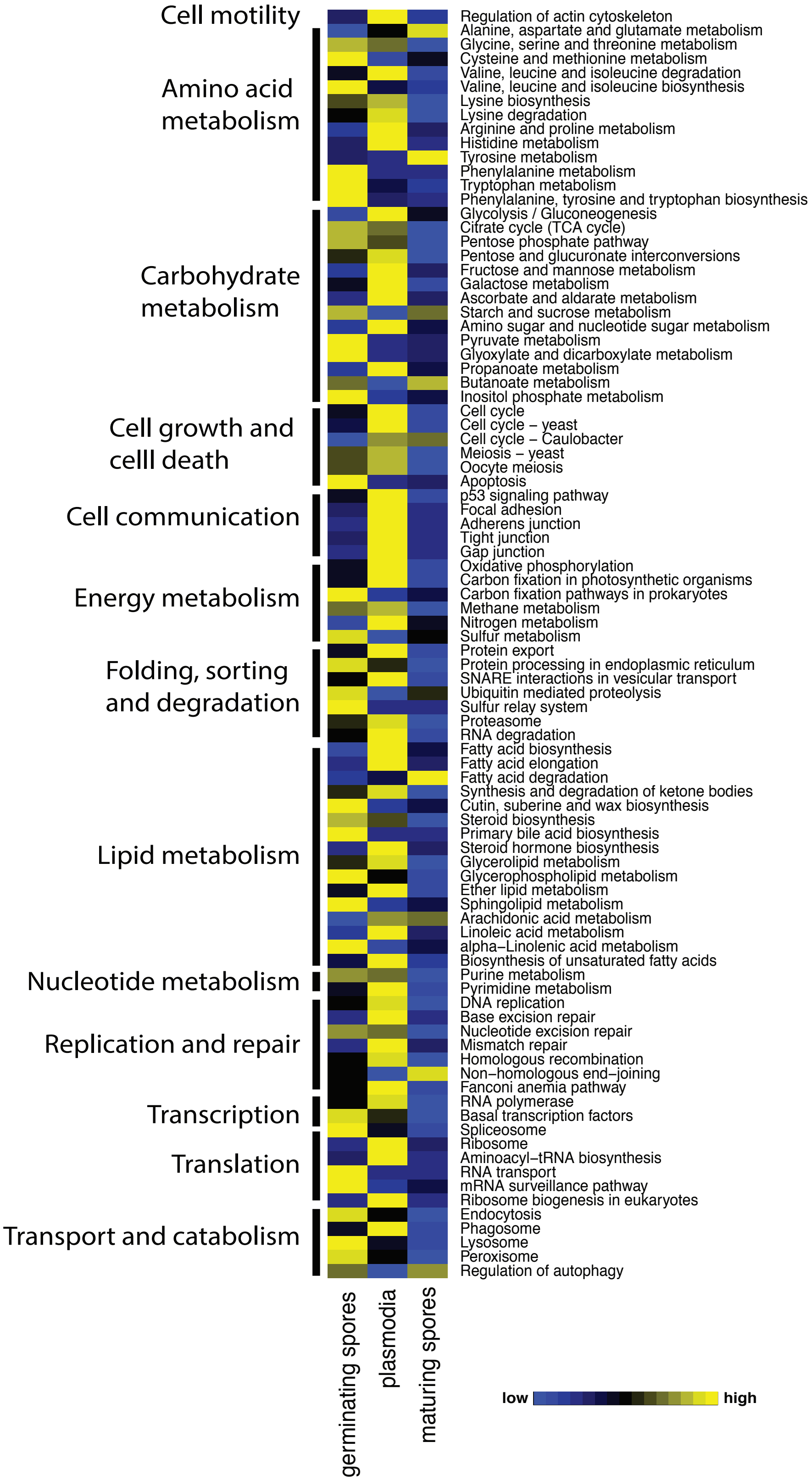

Supplementary Fig. S15. Life-stage specific gene expression patterns of *P. brassicae*. Gene expression pattern of three *P. brassicae* life stages sorted according to their KEGG categories. Gene expression is visualized showing Z-scores for each gene within all transcriptome libraries.

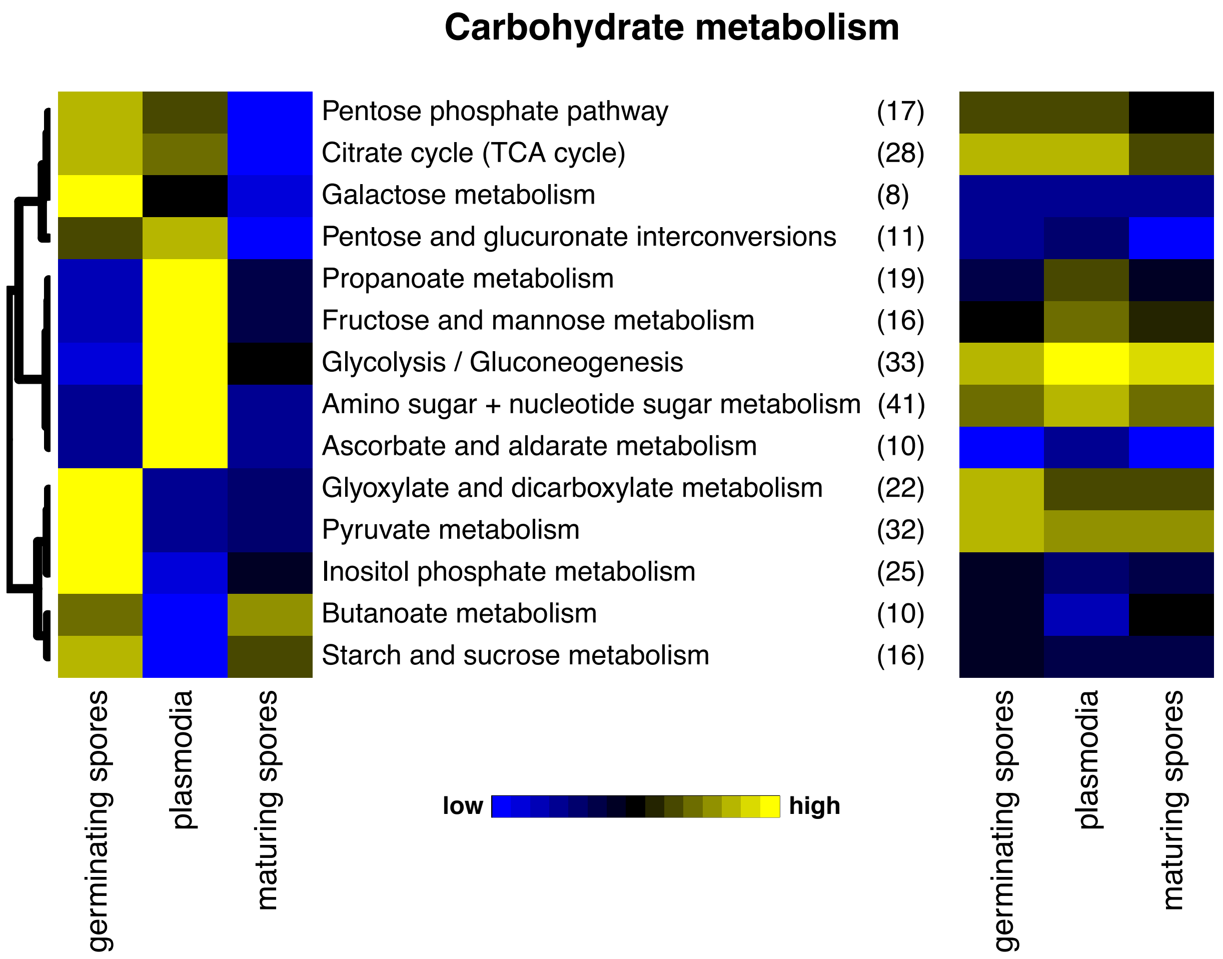

Supplementary Fig. S16. Gene expression of carbohydrate metabolic associated genes in *P. brassicae*. Gene expression for KEGG categories belonging to the carbohydrate metabolism. Number of genes for each category are shown in brackets. Gene expression pattern (left) is visualized showing Z-scores for each gene within all transcriptome libraries. Total gene expression (right) is visualized, showing log10 transformed FPKM values.

Trehalose-related enzymes

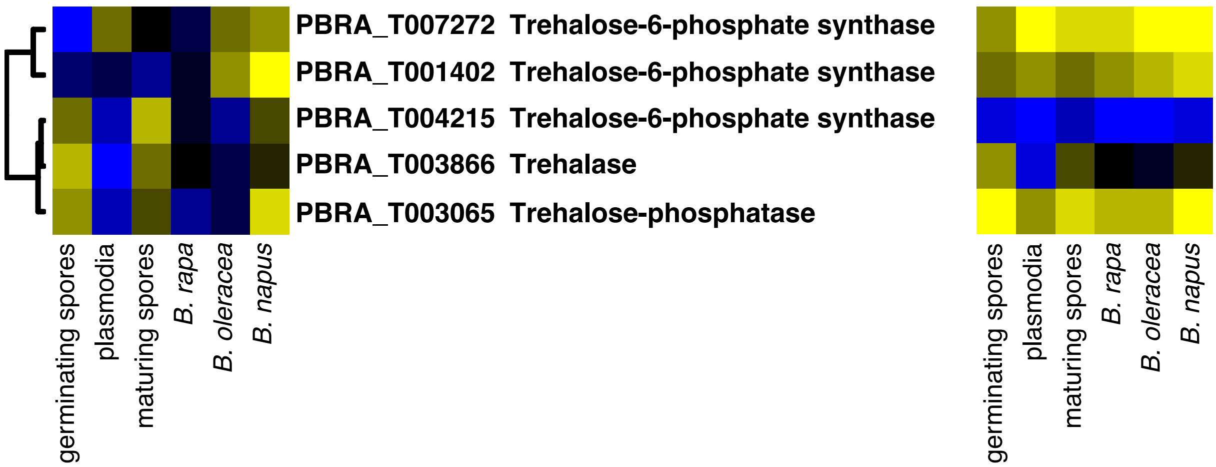

Supplementary Fig. S17. Expression profiles genes encoding of trehalose related metabolic enzymes in *P. brassicae*. Gene expression pattern (left) is visualized showing Z-scores for each gene within all transcriptome libraries. Total gene expression (right) is visualized, showing log10 transformed FPKM values.

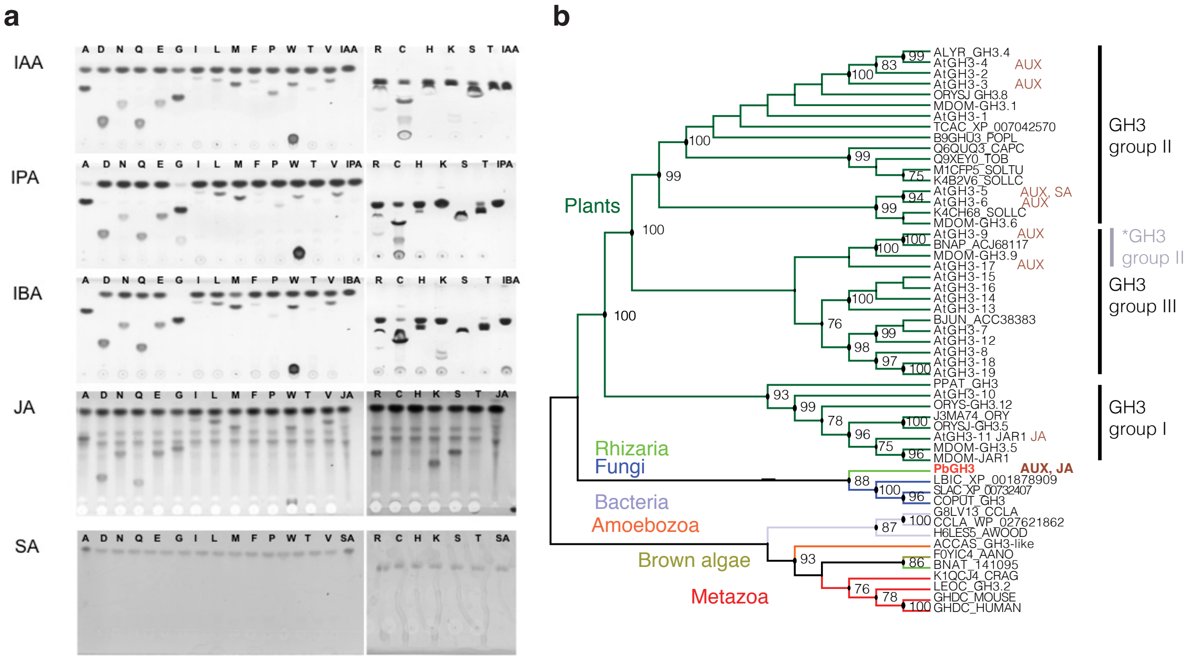

Supplementary Fig. S18. ***In vitro* activity and phylogeny of the *P. brassicae* GH3 protein (PBRA_T002258).** (a) TLC plates showing substrates and conjugation products. Indole-3-acetic acid (IAA), indole-3-propionic acid (IPA), indole-3-butyric acid (IBA), jasmonic acid (JA) and salicylic acid (SA). Amino acids are labeled according to the one-letter code. (b) GH3-domains were defined using SMART (http://smart.embl-heidelberg.de/) before analyzing as outlined in Supplementary Fig. S8. Bootstrap values >70% are shown. Full information on proteins, accession numbers and species are deposited in Supplementary Dataset 1. Scale bar represents the number of substitutions per site. Indicated are GH3 family groups I-III in plants and their substrates auxin (AUX), SA and JA. *GH3 proteins in group III previously clustered with group II are indicated with a gray bar.

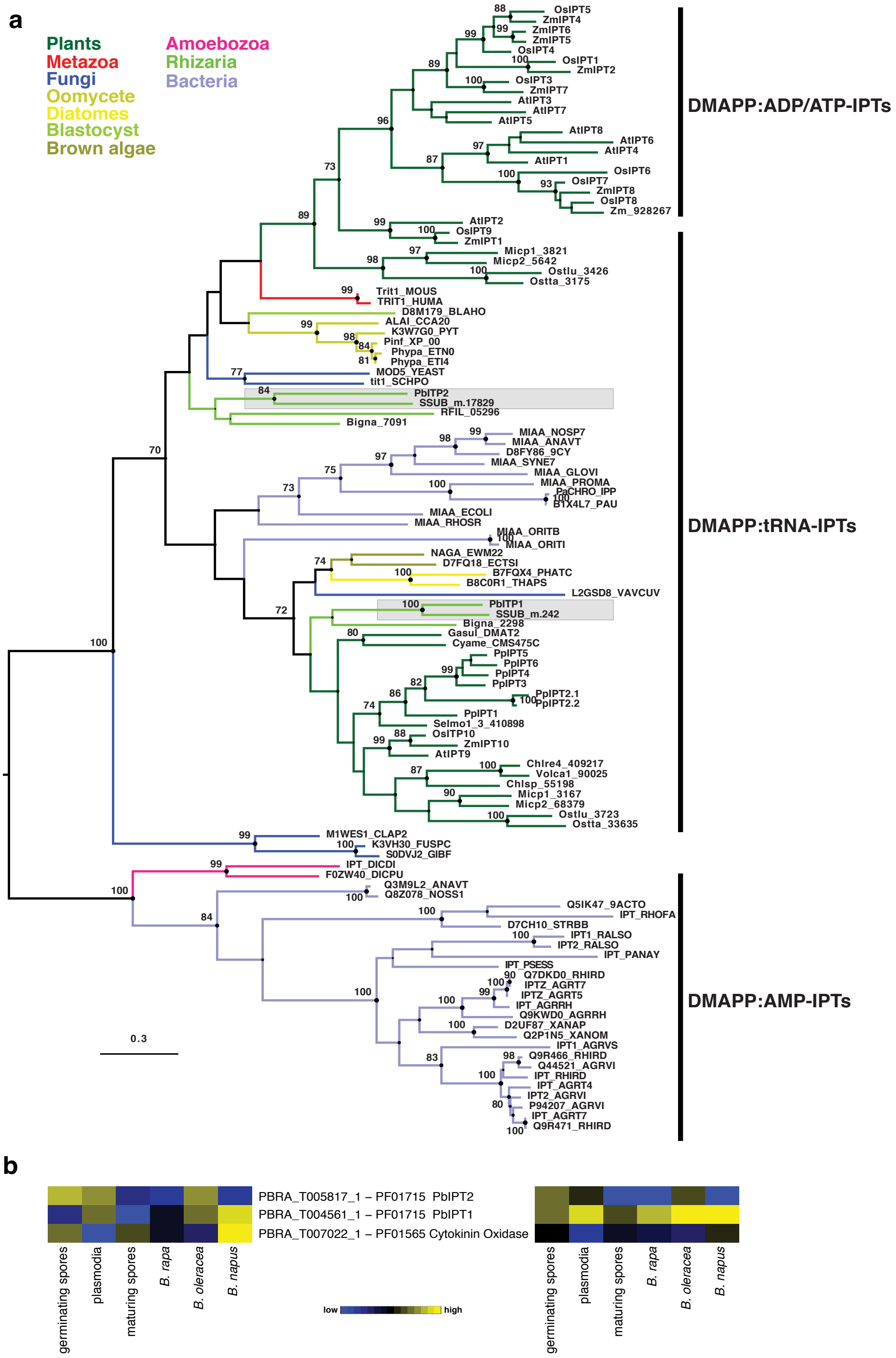

Supplementary Fig. S19. Phylogeny and transciptome analysis of isopentenyl-transferases (IPTs). (a) Phylogenetic analysis of *IPT* genes of Plasmodiophorids. IPT-domain proteins (PF01745; IPR002627) from the Plasmodiophorids, Rhizaria and additional proteins sequences from oomycetes, brown algae and blastocystis were analyzed with representative proteins containing the IPT protein domain from cyanobacteria, Alveolates, Euglenozoa, Stramenopiles, and *Agrobacterium tumefaciens* as earlier described[9]. Full

information about abbreviations of proteins, accession number and species used in the analyses are deposit in Supplementary Dataset 1. Alignments were performed using T-Coffee 8.14[6] Phylogenetic analyses were conducted in MEGA[7] using the Maximum Likelihood method based on the JTT matrix-based model[10] and bootstrapping was with 1,000 replicates (values >70 are shown). (b) Expression of *IPT* genes and a potential cytokinin oxidase in different hosts and life-stages. Gene expression pattern (left) is visualized showing Z-scores for each gene within all transcriptome libraries. Total gene expression (right) is visualized, showing log10 transformed FPKM values.

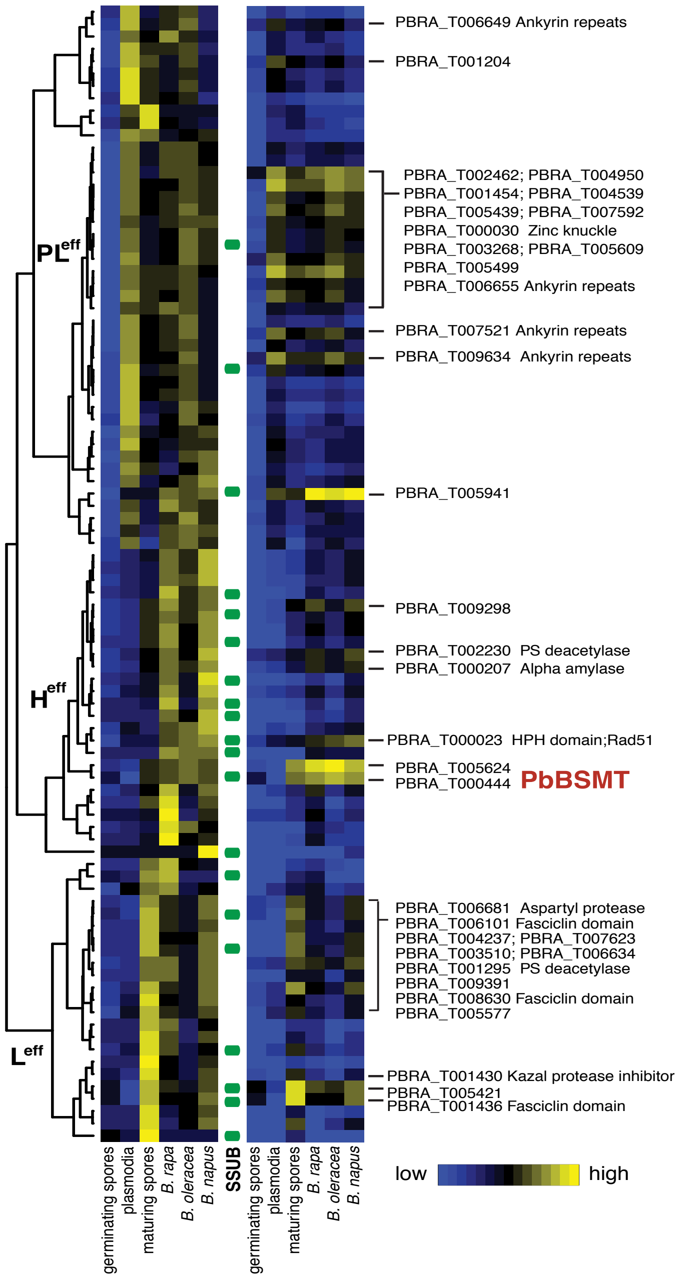

Supplementary Fig. S20. Gene expression of *P. brassica* effector candidates. Predicted secreted enzymes with a length below 450 amino acids are clustered into 3 groups (PL^{eff}, H^{eff}, L^{eff}) based on Pearson correlation of their expression patterns. Expression pattern normalized by Z-score (left) and absolute expression based on the FPKM value (right) are shown for 6 transcriptome libraries. Only candidates with at least 5 log-fold higher expression in at least one library compared to the plant free germinating spore sample are shown. Gene IDs and Pfam-descriptions of higher expressed genes are shown. Homologues in *S. subterranea* are indicated with a green bar in column SSUB. A full list of effector candidates is given in Supplementary Table S9. PS = Polysaccharide; PbBSMT = SA-methyltransferase

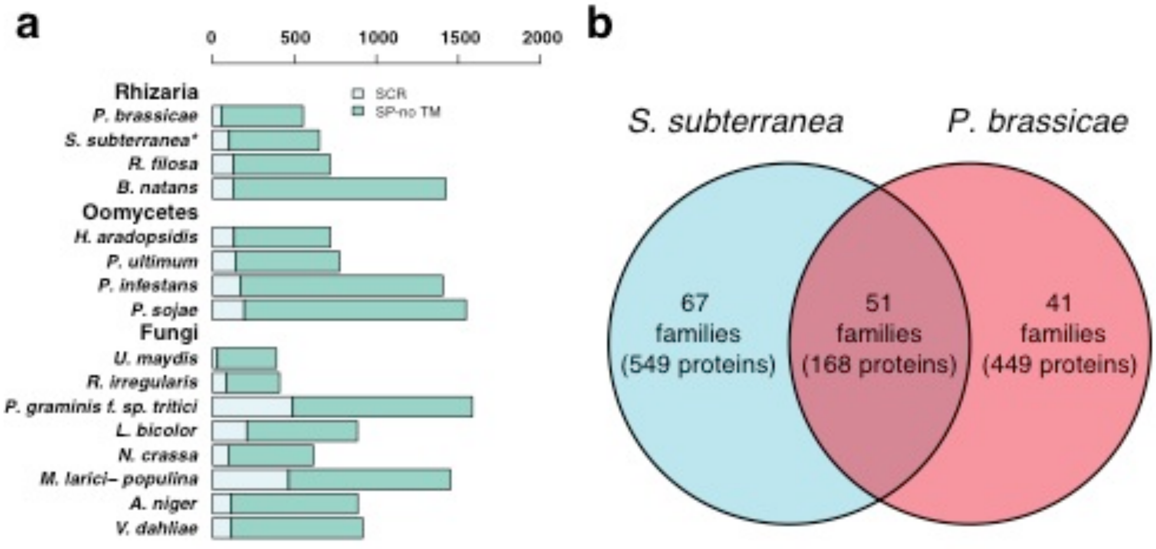

Supplementary Fig. S21. Secreted proteins. (a) Comparison of numbers of predicted secreted proteins in the Rhizaria, selected fungi and oomycete plant pathogens**.** Secreted proteins were predicted when a signal-peptide (SP) but no transmembrane domain was present. The number of small cysteine-rich protein (SCR) is defined by proteins smaller as 450 amino acids in length and more than 3% cysteines. (b) OrthoMCL[11] comparison of the secretomes of *P. brassicae* and *S. subterranea*. Shown are the number of protein families and the total number of proteins (including singletons) in brackets.

**S. subterranea* numbers are based on transcriptome only.

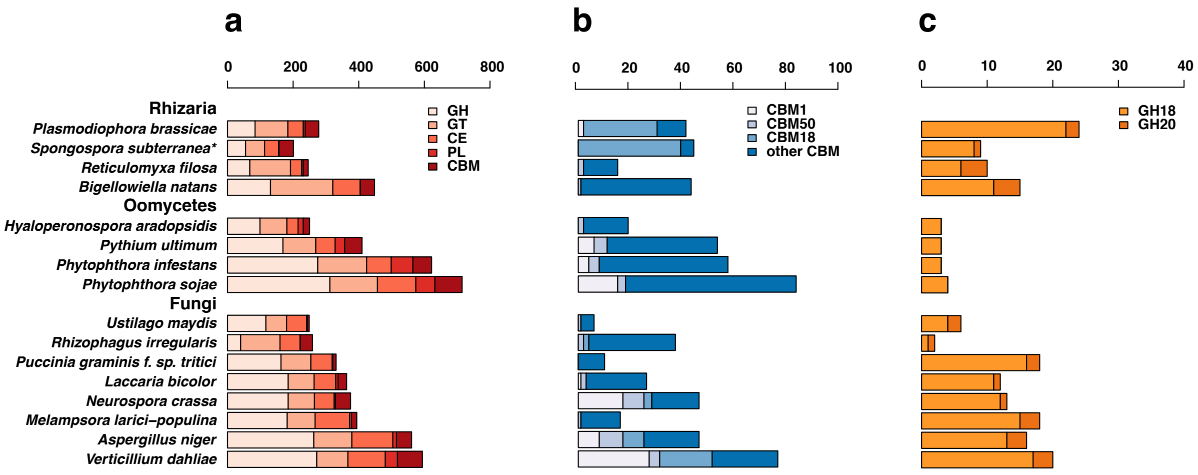

Supplementary Fig. S22. Total numbers of CAZymes (12) in Rhizaria, and selected plant pathogenic oomycetes and fungi. (a) CAZY domains belonging to glycosyl hydrolases (GH), glycosyl transferases (GT), carbohydrate esterases (CE), pectate lyases (PL) and carbohydrate binding domains (CBM). (b) CAZY domains of CBMs, chitin-binding domains CBM18, CBM50 and the cellulose-binding domain CBM1. (c) CAZY domains of GH18 and GH20 chitinases.

**S. subterranea* numbers are based on transcriptome only.

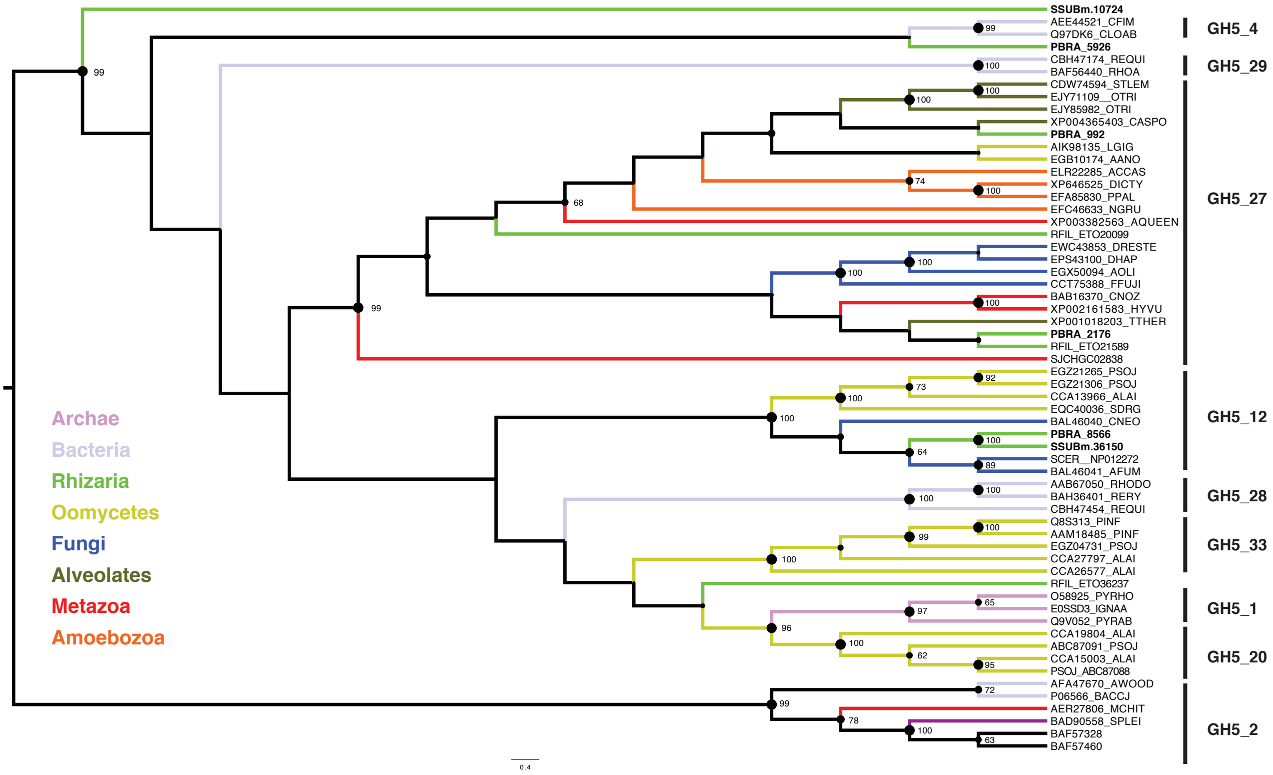

Supplementary Fig. S23. Phylogenetic analysis of GH5 proteins. Phylogenetic tree of GH5 modules was constructed using MEGA6 platform with Maximum Likelihood method based on the JTT matrix-based model (10) from a T-Coffee 8.14[6] alignment, and bootstrapping was with 1,000 replicates (values >60% are shown). The Plasmodiophorid GH5-proteins are indicated in bold. GH5 families are assigned as earlier described[12,13]. Full information about abbreviations of proteins, accession number and species used in the analyses are deposit in Supplementary Dataset 1.

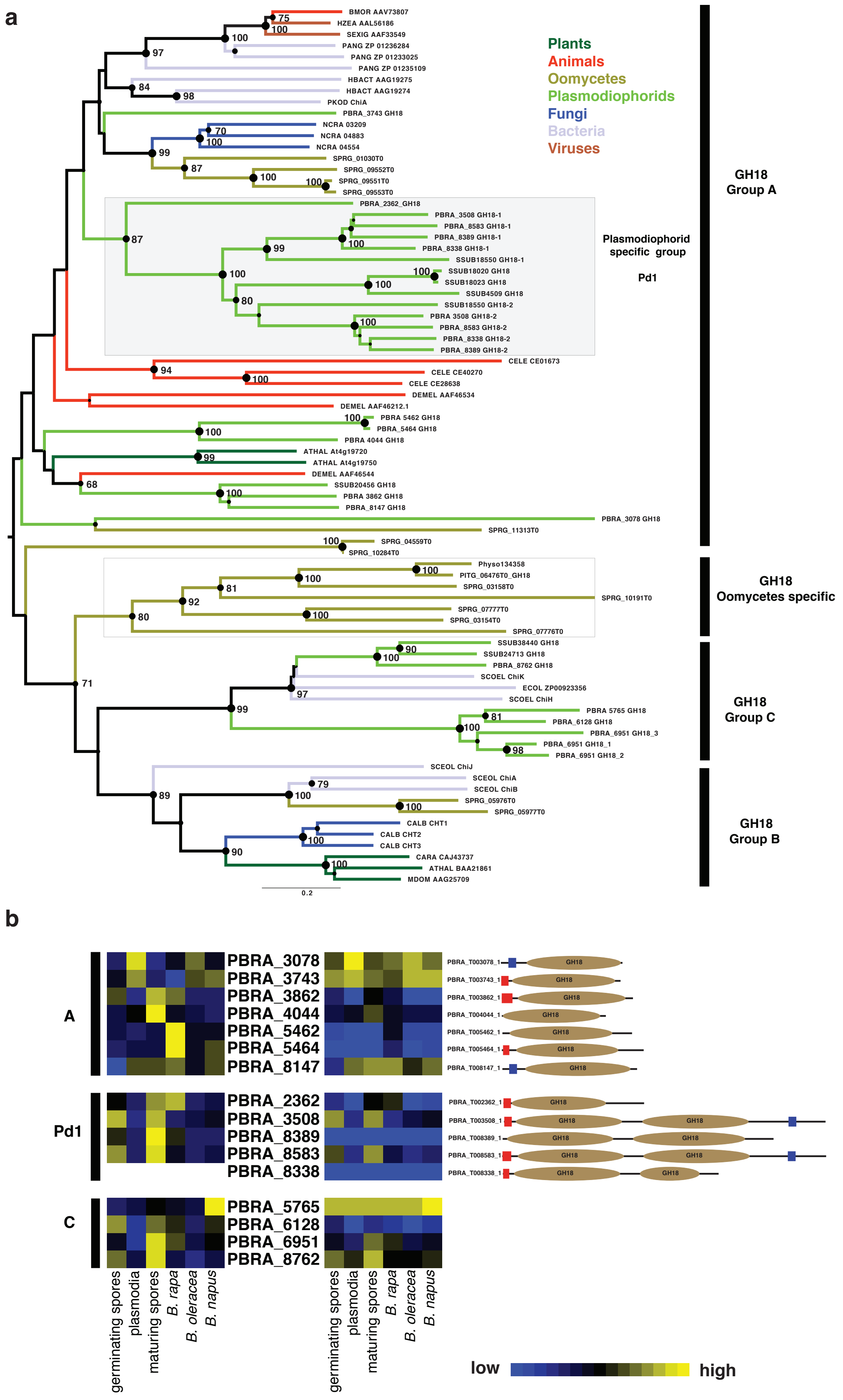
a
Plants
Animals
Oomycetes
Plasmodiophorids
Fungi
Bacteria
Viruses
BMOR AAV73807
HZEA AAL56186
SEXIG AAF33549
PANG ZP 01236284
PANG ZP 01233025
PANG ZP 01235109
HBACT AAG19275
HBACT AAG19274
PKOD ChiA
PBRA_3743 GH18
NCRA 03209
NCRA 04883
NCRA 04554
SPRG_01030T0
SPRG_09552T0
SPRG_09551T0
SPRG_09553T0
PBRA_2362_GH18
PBRA_3508 GH18-1
PBRA_8583 GH18-1
PBRA_8389 GH18-1
PBRA_8338 GH18-1
SSUB18550 GH18-1
SSUB18020 GH18
SSUB18023 GH18
SSUB4509 GH18
SSUB18550 GH18-2
PBRA 3508 GH18-2
PBRA_8583 GH18-2
PBRA_8338 GH18-2
PBRA_8389 GH18-2
CELE CE01673
CELE CE40270
CELE CE28638
DEMEL AAF46534
DEMEL AAF46212.1
PBRA 5462 GH18
PBRA_5464 GH18
PBRA 4044 GH18
ATHAL At4g19720
ATHAL At4g19750
DEMEL AAF46544
SSUB20456 GH18
PBRA 3862 GH18
PBRA_8147 GH18
PBRA_3078 GH18
SPRG_11313T0
SPRG_04559T0
SPRG_10284T0
Physo134358
PITG_06476T0_GH18
SPRG_03158T0
SPRG_10191T0
SPRG_07777T0
SPRG_03154T0
SPRG_07776T0
SSUB38440 GH18
SSUB24713 GH18
PBRA_8762 GH18
SCOEL ChiK
ECOL ZP00923356
SCOEL ChiH
PBRA 5765 GH18
PBRA_6128 GH18
PBRA_6951 GH18_3
PBRA_6951 GH18_1
PBRA_6951 GH18_2
SCEOL ChiJ
SCEOL ChiA
SCEOL ChiB
SPRG_05976T0
SPRG_05977T0
CALB CHT1
CALB CHT2
CALB CHT3
CARA CAJ43737
ATHAL BAA21861
MDOM AAG25709
0.2
Plasmodiophorid specific group
Pd1
GH18 Group A
GH18 Oomycetes specific
GH18 Group C
GH18 Group B
b
A
Pd1
C
PBRA_3078
PBRA_3743
PBRA_3862
PBRA_4044
PBRA_5462
PBRA_5464
PBRA_8147
PBRA_2362
PBRA_3508
PBRA_8389
PBRA_8583
PBRA_8338
PBRA_5765
PBRA_6128
PBRA_6951
PBRA_8762
PBRA_T003078_1
PBRA_T003743_1
PBRA_T003862_1
PBRA_T004044_1
PBRA_T005462_1
PBRA_T005464_1
PBRA_T008147_1
PBRA_T002362_1
PBRA_T003508_1
PBRA_T008389_1
PBRA_T008583_1
PBRA_T008338_1
GH18
germinating spores
plasmodia
maturing spores
B. rapa
B. oleracea
B. napus
low
high

Supplementary Fig. S24. Phylogeny and transcriptome analysis of the glycoside hydrolase 18 (*GH18*) gene family. (a) Phylogenetic tree of GH18 chitinase domains. All *P. brassicae* and *S. subterranea* gene models were blasted to a reference set[14] of GH18 domains. Evolutionary analyses for the GH18 modules were conducted in MEGA6 using the Maximum Likelihood method based on the JTT matrix-based model from a ClustalW[15] alignment of the predicted amino acid sequences of GH18 modules, and bootstrapping was with 1,000 replicates (values >70 are shown). The main GH18 groups A, B and C are assigned as defined by[14]. Full information about abbreviations of proteins, accession number and species used in the analyses are deposit in Supplementary Dataset 1. The Plasmodiophorid specific group Pd1 appeared to be evolved from gene duplications and internal GH18 domain duplications. (b) Gene expression of the *GH18* genes in the *P. brassicae* genome and diagrammatic representation of their corresponding protein domains. Gene expression pattern (left) is visualized showing *Z*-scores for each gene within all transcriptome libraries. Total gene expression (right) is visualized, showing log10 transformed FPKM values. GH18-domain genes are grouped into the phylogenetic GH18 groups A, Pd1 and C as in (a). Brown oval indicate the GH18 domain, transmembrane domains are shown in blue and secretion signals in red. The missing and shorten domains of PBRA_T008338 and PBRA_T008389 and their low expression suggest they might be pseudogenes.

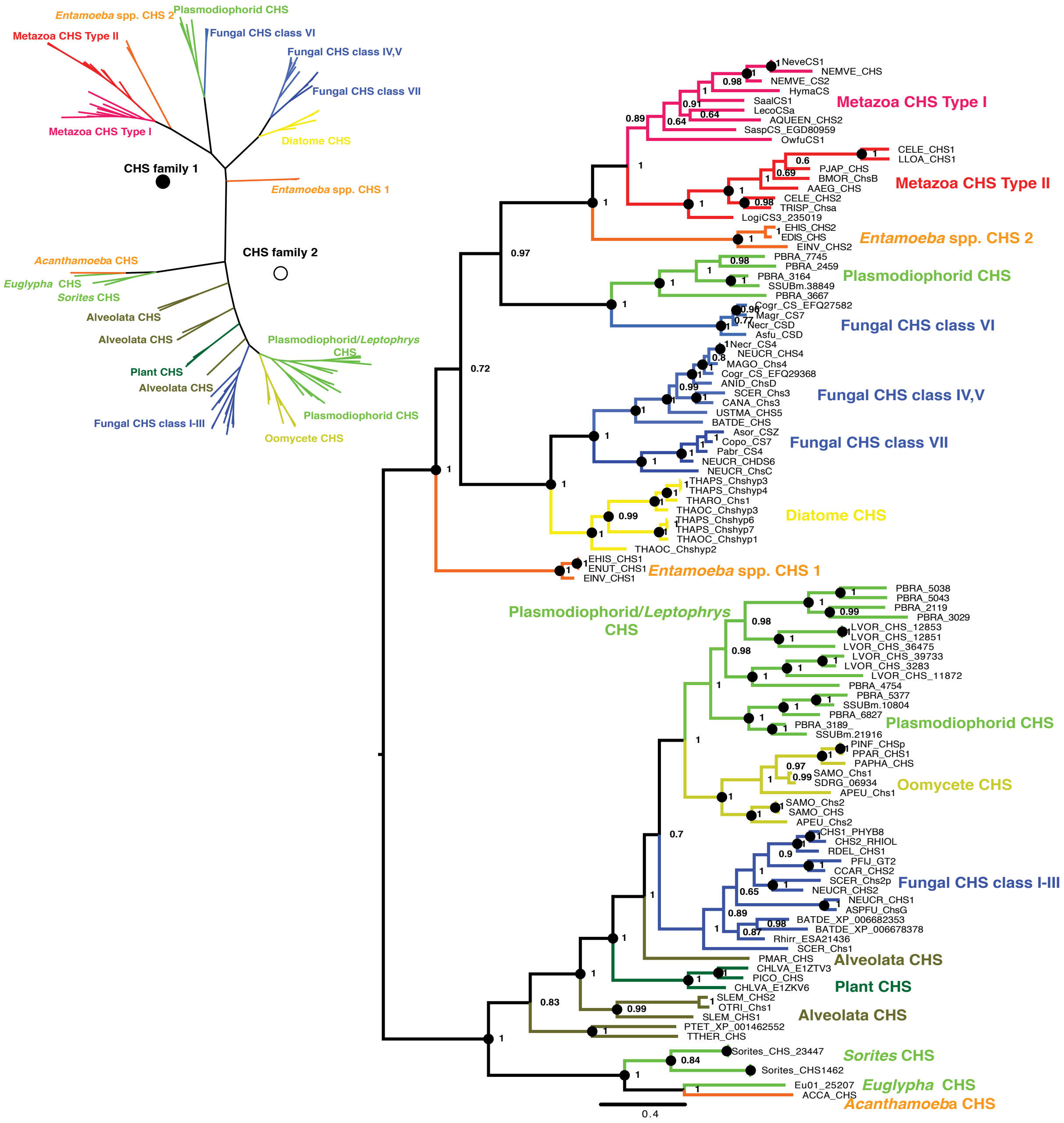

Supplementary Fig. S25. Detailed tree for CHS family domains. Unrooted Bayesian phylogenetic reconstruction of chitin synthase CHS domains (Chitin_synth_2; PF03142). Values on nodes represent node support as posterior probabilities. Black circles indicate nodes supported of parallel ML analysis (RAxML, LG model, 500 replicates) showing a bootstrap >70. The small tree shows the unrooted tree while the larger tree depicted is mid-point rooted for clarity. Proteins used in the analyses are listed in Supplementary Dataset 1.

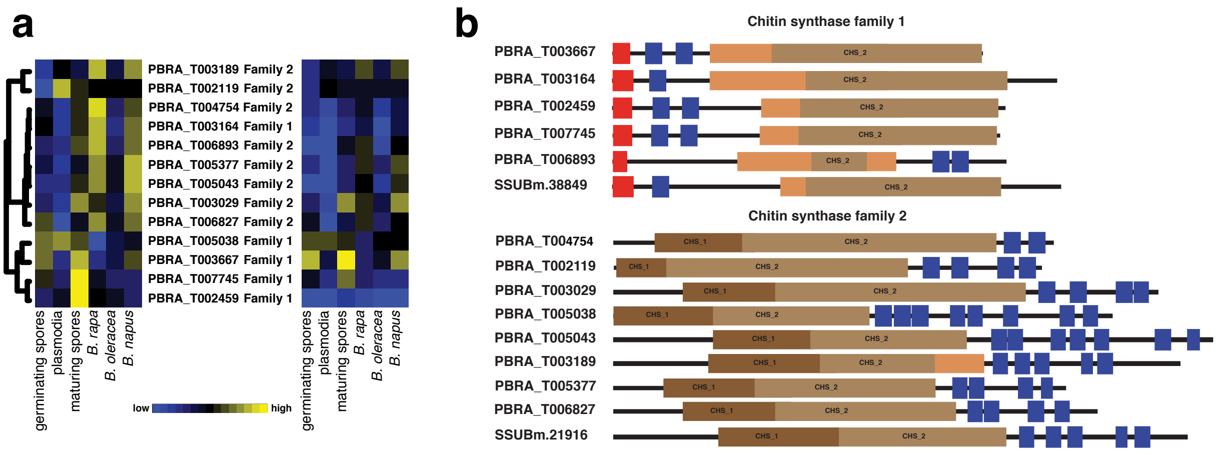

Supplementary Fig. S26. Gene expression and protein domain organization of Plasmodiophorid chitin synthases. (a) Expression of the *P. brassicae* chitin synthase (CHS) genes. Gene expression pattern (left) is visualized showing Z-scores for each gene within all transcriptome libraries. Total gene expression (right) is visualized, showing log10 transformed FPKM values. Blue is the lowest expression and yellow the highest expression. (b) Domain organization for both CHS families in the Plasmodiophorids. Family one includes a secretion peptide (red) and transmembrane domains (blue). The Pfam chitin synthase domain CHS_2 (brown) of family 1 overlaps partly with GT domains (light brown), while in family 2 the CHS_2 domain overlaps with CHS_1 domains. There are no secretion peptides in family 2 but several C-terminal transmembrane domains. The domain organization for family 1 and family 2 in the Plasmodiophorids resemble the domain organization of the fungal CHS classes VI and I-III, respectively.

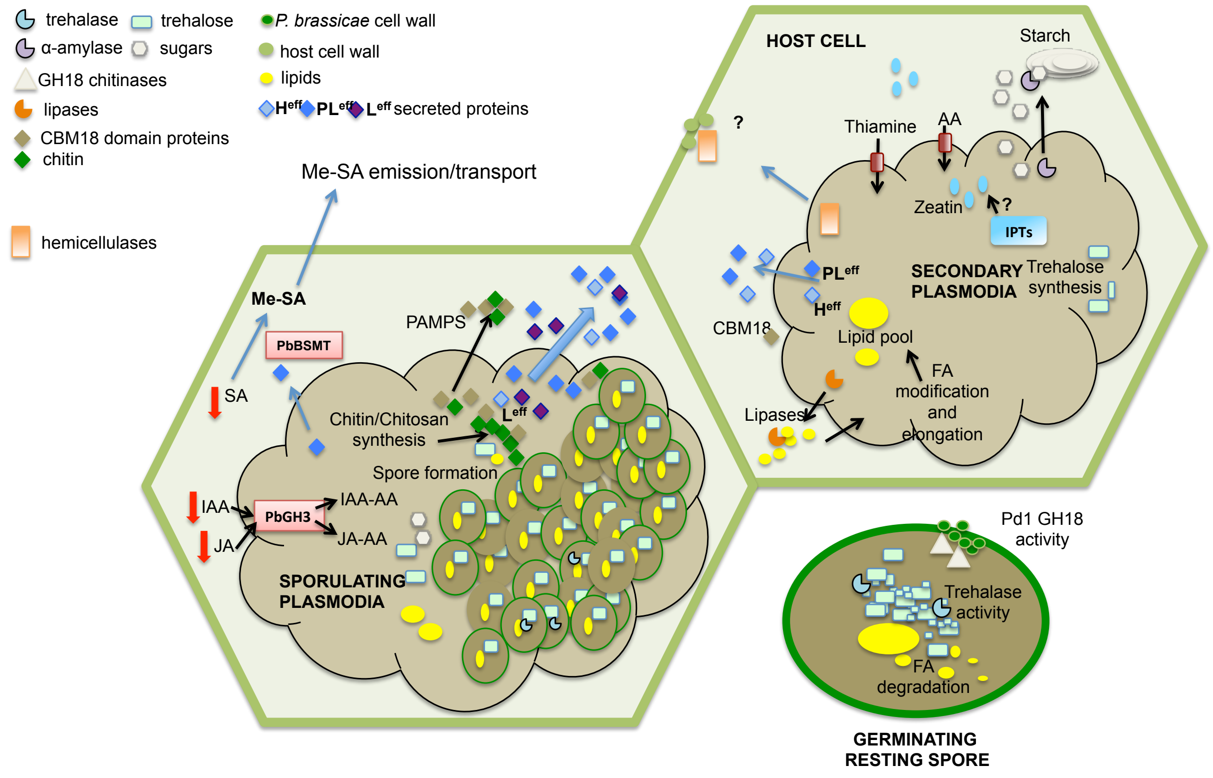

Supplementary Fig. S27. A schematic model of molecular activity of *P. brassicae* at different development stages. *P. brassicae* is reduced in lipid, amino acid, nitrogen, sulfur and thiamine metabolism. The plasmodia need to assimilate metabolites such as AA, lipids or thiamine from their host. Thiamine transporters can supply *P. brassicae* with thiamine from the host. *P. brassicae* secondary plasmodia could acquire sugars by hydrolyzing starch from the host cell via a secreted α-amylase and lipids through secreted lipases. In general the housekeeping metabolism is highest in the plasmodial stage leading to the production of lipids and sugars such as trehalose either for direct use or as for energy storage. *P. brassicae* might be able to modulate the plant's hormone homeostasis by either biosynthesis of cytokinins[16] or reducing free auxin to inactive amino acid conjugates via the PbGH3 conjugase activity. This leads to the club root/gall phenotype. Potential hemicellulases of *P. brassicae* could be involved in expansion of the host cell walls. PbGH3 might also reduce the JA-Ile content in the host by conjugating JA to other amino acids avoiding JA-triggered immune responses of the host. The secreted H^{eff}, PL^{eff} and L^{eff} proteins (Supplementary Table S9) have the potential to regulate the host metabolism and defense response by on protein level by interfering with the host ubiquitination system or digest plant proteins and protect *P. brassicae* from proteases. When secondary plasmodia develop into resting spores chitin is synthesized. The

methyltransferase PbBSMT might reduce the SA levels in the infected host tissue at this developmental stage. The CE4-domain proteins convert chitin into chitosan. Chitosan is a weaker PAMP than chitin[17] and might help to avoid PAMP triggered immunity. Both Plasmodiophorids show enrichments in CBM18 domain in the secretome, and some proteins could have a similar function as the LysM effectors in fungi. Later in the life cycle germinating resting spores appear to obtain some energy from storage molecules such as trehalose or lipids such as arachidonate acid (ARA), which is present in *P. brassicae* resting spores[18]. Trehalose is also an osmoregulator and can provide freezing protection for fungal spores or animals by replacing intracellular water[19]. The trehalose synthesis could be an important contributor to the long-term survival of the resting spores in the soil. In the germinating process the resting spore cell wall needs to be degraded which is indicated by an increased expression of the Pd1-GH18 chitinases at this stage. Trehalose is degraded and the TCA, PPP and glycolysis enzymes are active. The high activities of enzymes of the glyoxylate cycles suggest a high conversion of lipids and sugars at this stage. However, a role of trehalose in other energy and nitrogen metabolism pathways as seen in the rice blast fungus[20] cannot be excluded.

Abbreviations: AA, amino acid; CBM18, carbohydrate/chitin binding module 18; FA, fatty acid; IAA, indole-3-acetic acid; IPT; isopentenyltransferase; JA, jasmonic acid; MeSA, methylated SA; PAMPs, pathogen-associated molecular patterns; PPP, pentose phosphate pathway, SA, salicylic acid; TCA, tricarbonic acid cycle

Supplementary Tables

Supplementary Table S1. DNA sequencing using Illumina HiSeq 2100 and Roche 454 FLX technology. Read number, read lengths, libraries and sequencing platforms used in this study.

Sample	Reads	Read length	Insert length	Sequencing platform
Illumina pair-end	196M	101 bp	200bp	SciLife Stockholm, Sweden
Illumina mate-pair	7M	90 bp	5kb	BGI, Hong Kong
454FLX	713k	776* bp	-	Macrogen, Korea
454FLX mate-pair	57k	414* bp	3kb	Macrogen, Korea

* Average read length

Supplementary able S2. Genome statistics for *Plasmodiophora brassicae* isolate e3.

Statistics of the Pbe3 genome	
Assembly size	24050826 nt
Number of assembled scaffolds	165
Number of scaffolds > 100 kb in length	53 (32%)
Number of scaffolds > 10 kb in length	116 (70.3 %)
Number of scaffolds > 1 kb in length	165 (100%)
Mean scaffold size	145763 nt
Median scaffold size	36592nt
Scaffold N50	472887 nt
Scaffold L50	16
Genomic GC content	58.53%
Scaffold %A	20.03
Scaffold %T	20.02
Scaffold %C	29.26
Scaffold %G	29.27
Scaffold %N	1.43
Percentage of assembly in scaffolded contigs	97.2
Percentage of assembly in unscaffolded contigs	2.8
Average number of contigs per scaffold	6.3
Average length of break (>25 Ns) between contigs in scaffold	377
Average coding sequence length	1405 nt
Average exon length	305.28 nt
Average intron length	68.52 nt
Average introns per gene	3.89
Number of genes without introns	1662

Supplementary Table S3. RNA sequencing using Illumina HiSeq 2100 technology. Read number, read lengths, libraries and platforms used.

Sample	Million reads	Read length	Strand specific	Sequencing platform
B. rapa clubroot (5 weeks post infection)	110.7	90 bp	No	BGI, Hong Kong
B. oleracea clubroot (6 weeks post infection)	71.6	151 bp	Yes	SciLife Stockholm, Sweden
B. napus clubroot (6 weeks post infection)	86.1	151 bp	Yes	SciLife Stockholm, Sweden
P. brassicae enriched sample	293.14	151 bp	No	SciLife Stockholm, Sweden
P. *brassicae* maturing spores	229.0	151 bp	No	SciLife Stockholm, Sweden
P. brassicae germinating spores	263.3	151 bp	No	SciLife Stockholm, Sweden
P. brassicae plasmodia	308.3	151 bp	Yes	SciLife Stockholm, Sweden
S. subterranea potato galls	80.8	151 bp	Yes	SciLife Stockholm, Sweden

Supplementary Table S4. Repetitive elements and transposons in the *P. brassicae* genome detected by RepeatMasker.

Class	Count	bp Masked	%masked
**DNA**			
hAT-Ac	45	10327	0.04%
**LINE**			
LOA	23	8378	0.03%
RTE-BovB	55	26091	0.11%
RTE-X	5	1367	0.01%
**LTR**			
Copia	78	38914	0.16%
ERV1	2	286	0.00%
Gypsy	317	235611	0.98%
Gypsy-Cigr	26	13963	0.06%
Pao	26	17637	0.07%
**SINE**			
5S	34	4114	0.02%
Unknown	2390	724744	3.01%
Total interspersed	3001	1081432	4.50%
Low complexity	107	5090	0.02%
Simple repeat	4940	210014	0.87%
**Total**	**8048**	**1296536**	**5.39%**

Supplementary Table S5. CEGMA-based gene contents of the *P. brassicae*, *R. filosa* and *B. natans* genomes and the *S. subterranea* transcriptome.

	P. brassicae		***S. subteranea***		***R. filosa***		***B. natans***	
	**Proteins No.**	**Complete-ness %**	**Proteins No.**	**Complete-ness %**	**Proteins No.**	**Complete-ness %**	**Proteins No.**	**Complete-ness %**
**Complete**	228	91.94	123	49.60	181	72.98	177	71.23
Group 1	59	89.39	38	57.58	39	59.09	31	46.97
Group 2	50	89.29	23	41.07	40	71.23	38	67.86
Group 3	55	90.16	27	44.26	45	73.77	50	81.97
Group 4	64	98.46	35	53.85	57	87.69	58	89.23
**Partial**	233	93.95	170	68.55	204	82.26	196	79.03
Group 1	61	92.42	49	74.24	43	65.15	34	51.52
Group 2	51	91.07	37	66.07	48	85.71	45	80.36
Group 3	57	93.44	39	63.93	50	81.97	57	93.44
Group 4	65	98.48	45	69.23	63	96.92	60	92.31

Supplementary Table S6. Enriched Pfam domains in *P. brassicae* and *S. subterranea* proteins. Pfam domains are compared with fungi, oomycetes, *B. natans* and *R. filosa* (species as in Supplementary Fig. S7). Pfam domains enriched in both Plasmodiophorid data sets are shown in bold.

Pfam description	Pfam	Enrichment	p-value	#
***P. brassicae* Pbe3**				
Basic membrane protein	PF02608	21	1e-02	3
Variant-surface-glycoprotein phospholipase C	PF03490	21	1e-02	3
CHAP domain	PF05257	21	1e-02	3
Phage Tail Collar Domain	PF07484	21	1e-02	3
Alginate lyase	PF08787	21	1e-02	3
PBP superfamily domain	PF12727	21	1e-02	3
Leucine rich repeats (6 copies)	PF13306	17	5e-48	47
Receptor family ligand binding region	PF01094	16,1	5e-12	13
DYW family of nucleic acid deaminases	PF14432	15,8	2e-22	24
Periplasmic binding protein	PF13458	14	7e-10	12
**RAP domain**	**PF08373**	13,5	6e-15	18
Periplasmic binding protein domain	PF13407	12,6	9e-02	3
Domain of unknown function (DUF4395)	PF14340	12,6	9e-02	3
TM1410 hypothetical-related protein	PF03537	11,9	2e-09	13
PAS domain	PF13188	11,7	4e-03	5
Reduced folate carrier	PF01770	10	3e-06	10
7 transmembrane sweet-taste receptor of 3 GCPR	PF00003	9,9	4e-26	39
**Galactose binding lectin domain**	**PF02140**	9,3	3e-07	12
Late embryogenesis abundant protein	PF03168	8,8	2e-02	5
Protein of unknown function (DUF2723)	PF11028	8,4	8e-02	4
Bacterial extracellular solute-binding protein	PF01547	8	1e-05	11
Protease prsW family	PF13367	7,9	8e-03	6
Succinylglutamate desuccinylase / Aspartoacylase family	PF04952	7	1e-02	6
Protein of unknown function (DUF563)	PF04577	6,7	6e-13	27
**Chitin recognition protein**	**PF00187**	6,6	3e-07	16
Bacterial extracellular solute-binding protein	PF13416	6,5	3e-03	8
PAP2 superfamily C-terminal	PF14360	6,3	1e-03	9
Zinc-finger domain of monoamine-oxidase A repressor R1	PF10497	5,8	3e-04	11

Large-conductance mechanosensitive channel, MscL	PF01741	5,5	6e-02	6
Protein of unknown function (DUF3638)	PF12340	5,5	6e-02	6
PPR repeat	PF01535	5,2	7e-13	34
Regulator of G protein signaling domain	PF00615	5	3e-26	71
PAS fold	PF08448	4,9	6e-04	12
SRF-type transcription factor (DNA-binding and dimerisation domain)	PF00319	4,8	2e-03	11
Cid1 family poly A polymerase	PF03828	4,7	3e-07	22
Mechanosensitive ion channel	PF00924	4,5	8e-04	13
PAS domain	PF13426	4,3	2e-05	19
YheC/D like ATP-grasp	PF14398	4,1	6e-02	8
PPR repeat family	PF13041	4,1	3e-15	52
MORN repeat	PF02493	4,1	3e-04	16
Pentatricopeptide repeat domain	PF13812	4	1e-05	21
GCC2 and GCC3	PF07699	3,9	6e-03	12
PPR repeat	PF12854	3,9	2e-04	18
Rhomboid family	PF01694	3,7	3e-03	14
Chitin synthase	PF01644	3,5	9e-02	9
His Kinase A (phospho-acceptor) domain	PF00512	3,3	1e-02	14
**Polysaccharide deacetylase**	**PF01522**	3,3	5e-04	20
PAS fold	PF00989	3,2	9e-02	10
Leucine rich repeat	PF13855	3,2	1e-19	92
Response regulator receiver domain	PF00072	2,9	5e-03	19
Leucine Rich repeats (2 copies)	PF12799	2,8	3e-15	89
Chitin synthase	PF03142	2,6	9e-02	14
**Tetratricopeptide repeat**	**PF13371**	2,5	4e-02	18
**Ankyrin repeat**	**PF13606**	2,5	2e-17	127
C2H2-type zinc finger	PF13894	2,4	4e-04	33
**Ankyrin repeat**	**PF00023**	2,3	1e-22	187
Anaphase-promoting complex subunit 11 RING-H2 finger	PF12861	2,3	2e-02	24
Lipase (class 3)	PF01764	2,3	3e-02	23
**Ankyrin repeats (many copies)**	**PF13857**	2,2	3e-21	187
Zinc-finger double domain	PF13465	2,1	2e-04	48
**Ankyrin repeats (many copies)**	**PF13637**	2,1	6e-20	199

RING-H2 zinc finger	PF12678	2,1	4e-05	58
Zinc finger, C2H2 type	PF00096	2,1	4e-04	48
Histidine kinase-, DNA gyrase B-, and HSP90-like ATPase	PF02518	2	6e-02	25
Zinc finger, C3HC4 type (RING finger)	PF00097	1,9	3e-06	83
Zinc finger, C3HC4 type (RING finger)	PF13920	1,9	3e-06	85
**Ankyrin repeats (3 copies)**	**PF12796**	1,9	2e-16	214
Zinc finger, C3HC4 type (RING finger)	PF13923	1,9	3e-08	115
Cyclic nucleotide-binding domain	PF00027	1,9	3e-02	36
Ring finger domain	PF13639	1,7	2e-05	104
Tetratricopeptide repeat	PF13432	1,6	8e-02	52
ADP-ribosylation factor family	PF00025	1,5	6e-02	69
S. subterranea				
von Hippel-Lindau disease tumour suppressor protein	PF01847	25,8	1e-02	3
Putative viral replication protein	PF02407	25,8	1e-02	3
Domain of unknown function (DUF3534)	PF12053	20,6	4e-03	4
Gem-associated protein 7 (Gemin7)	PF11095	17,2	1e-04	6
Mcm10 replication factor	PF09332	17,2	9e-03	4
CHDNT (NUC034) domain	PF08073	15,5	8e-02	3
TLC ATP/ADP transporter	PF03219	14,7	2e-02	4
NF-X1 type zinc finger	PF01422	13,8	2e-05	8
**Galactose binding lectin domain**	**PF02140**	10,5	2e-06	11
Reeler domain	PF02014	10,3	4e-03	6
Domain of unknown function (DUF4097)	PF13349	9,9	2e-02	5
Dopey, N-terminal	PF04118	9,2	3e-02	5
**Chitin recognition protein**	**PF00187**	9,1	3e-10	18
Gpi16 subunit, GPI transamidase component	PF04113	7,4	3e-03	8
Biotin-protein ligase, N terminal	PF09825	7,2	8e-02	5
Histone H1-like nucleoprotein HC2	PF07382	7	3e-02	6
HCO3- transporter family	PF00955	6,7	3e-02	6
IPT/TIG domain	PF01833	6,4	2e-06	16
Peptidyl-tRNA hydrolase PTH2	PF01981	6	2e-02	7
Transposase	PF01527	5,9	1e-02	8
Phosphate transporter family	PF01384	5,7	8e-02	6

**RAP domain**	**PF08373**	5,5	9e-02	6
Domain of unknown function (DUF3336)	PF11815	5,5	2e-03	11
OTU-like cysteine protease	PF02338	5	6e-08	24
Amidohydrolase	PF13147	3,6	4e-02	11
Putative serine esterase (DUF676)	PF05057	3,6	2e-02	13
Hsp20/alpha crystallin family	PF00011	3,4	6e-02	11
Amidohydrolase family	PF01979	3,4	4e-03	17
tRNA synthetases class II (D, K and N)	PF00152	3,3	2e-03	19
**Ankyrin repeats (many copies)**	**PF13857**	3,3	1e-49	222
gag-polypeptide of LTR copia-type	PF14244	3,1	4e-04	24
**Polysaccharide deacetylase**	**PF01522**	3	3e-02	15
**Ankyrin repeat**	**PF00023**	2,9	7e-35	190
**Ankyrin repeat**	**PF13606**	2,9	1e-21	121
**Ankyrin repeats (3 copies)**	**PF12796**	2,8	2e-46	258
**Ankyrin repeats (many copies)**	**PF13637**	2,8	2e-38	216
**Tetratricopeptide repeat**	**PF13371**	2,7	6e-02	16
DDE superfamily endonuclease	PF13359	2,5	1e-02	23
DDE superfamily endonuclease	PF03184	2,5	4e-04	36
SET domain	PF00856	2,4	7e-05	44
Chromo (CHRromatin Organisation MOdifier) domain	PF00385	2,1	3e-03	40
HECT-domain (ubiquitin-transferase)	PF00632	2,1	1e-01	24
Mitochondrial carrier protein	PF00153	1,9	4e-03	51

Supplementary Table S7. Enriched Pfam domains in *P. brassicae* and *S. subterranea* secretomes. Pfam domains are compared with the secretomes of fungi, oomycetes, *B. natans* and *R. filosa* (species as in Supplementary Fig. S7). Pfam domains enriched in both Plasmodiophorid data sets are shown in bold.

Pfam description	Pfam ID	Enrichment	p-value	#
***P. brassicae* Pbe3**				
Kazal-type serine protease inhibitor domain	PF00050	19,9	2e-02	3
Thaumatin family	PF00314	19,9	2e-02	3
Kazal-type serine protease inhibitor domain	PF07648	19,9	2e-02	3
Cathepsin propeptide inhibitor domain (I29)	PF08246	16	8e-03	4
Papain family cysteine protease	PF00112	15	1e-04	6
Fasciclin domain	PF02469	15	9e-02	3
**Cysteine-rich secretory protein family**	**PF00188**	12	8e-04	6
**Chitin recognition protein**	**PF00187**	11,2	6e-06	9
**Eukaryotic aspartyl protease**	**PF00026**	11,1	8e-03	5
Leucine rich repeats (6 copies)	PF13306	8,1	1e-10	19
**Polysaccharide deacetylase**	**PF01522**	7	8e-03	7
**Ankyrin repeat**	**PF13606**	5,5	1e-14	35
**Ankyrin repeat**	**PF00023**	5,2	4e-20	49
**Ankyrin repeats (many copies)**	**PF13857**	5,2	4e-20	49
**Ankyrin repeats (many copies)**	**PF13637**	5,1	3e-20	51
**Leucine rich repeat**	**PF13855**	5	3e-08	23
**Ankyrin repeats (3 copies)**	**PF12796**	4,9	3e-20	53
Leucine Rich repeats (2 copies)	PF12799	4,9	1e-07	22
S. subterranea				
Filamin/ABP280 repeat	PF00630	36,1	4e-04	4
PA14 domain	PF07691	36,1	4e-04	4
**Cysteine-rich secretory protein family**	**PF00188**	27,1	3e-02	3
**Chitin recognition protein**	**PF00187**	25,1	2e-09	10
Common central domain of tyrosinase	PF00264	22,6	5e-03	4
**Eukaryotic aspartyl protease**	**PF00026**	16,4	2e-02	4

**Polysaccharide deacetylase**	**PF01522**	12	6e-02	4
**Ankyrin repeats (many copies)**	**PF13857**	4,5	2e-06	22
**Ankyrin repeats (3 copies)**	**PF12796**	4,4	3e-07	25
**Ankyrin repeats (many copies)**	**PF13637**	4,2	2e-05	20
**Ankyrin repeat**	**PF00023**	4	4e-04	17

Supplementary Table S8. Presence of amino acid motifs in the secreted proteins of *P. brassicae* and *S. subterranea*. Known pathogenicity related motifs and *de novo* discovered motifs (bold).

Mature sequence (secreted) or full sequence (non-secreted)								
P. brassicae					***S. subterranea***			
**AA motif**	**Secreted**	**Non-secreted**	**Cum_prob**	**Adjusted**	**Secreted**	**Non-secreted**	**Cum_prob**	**Adjusted**
R.LR	95	2081	1.00	1,00	39	1359	1.00	1,00
R.LQ	72	1416	0.95	1.00	36	1013	0.98	1,00
R.LR.{10,40}EER	1	23	0.75	0.98	0	6	1,00	1,00
R.LQ.{10,40}EER	1	13	0.56	0.81	0	5	1,00	1,00
CH.C	1	107	1.00	1.00	2	104	0.96	1,00
C.HC	9	142	0.49	0.80	1	137	0,71	1,00
LFLAK	1	5	0.30	0.55	0	2	1,00	1,00
**TPLHWAA**[1]	14	18	>0.01	>0.01	0	0	1.00	1.00
**PGIDVNAR**[1]	10	12	>0.01	>0.01	0	0	1.00	1.00
**TPLHLAV**[1]	6	10	>0.01	>0.01	1	20	0.63	1.00
**CCSQYGWC**[2]	2	1	>0.01	0.02	0	0	1.00	1.00
**GIDVNARD**[1]	10	15	>0.01	>0.01	0	0	1.00	1.00
**I[AM]STKRTW**	6	3	>0.01	>0.01	0	0	1.00	1.00

100 AA after SP (secreted) or after ATG (non-secreted)								
P. ***brassicae***					***S. subterranea***			
**AA motif**	**Secreted**	**Non-secreted**	**Cum_prob**	**Adjusted**	**Secreted**	**Non-secreted**	**Cum_prob**	**Adjusted**
R.LR	19	631	1,00	1,00	8	501	1,00	1,00
R.LQ	21	332	0,45	0,97	4	346	1,00	1,00
R.LR.{10,40}EER	0	2	1,00	1,00	0	0	1,00	1,00
R.LQ.{10,40}EER	1	3	0,21	0,68	0	1	1,00	1,00
CH.C	1	35	0,88	1,00	1	38	0,85	1,00
C.HC	4	43	0,28	0,72	1	48	0,90	1,00
LFLAK	0	1	1,00	1,00	0	0	1,00	1,00
**TPLHWAA**[1]	1	0	0.06	0.18	0	0	1.00	1.00
**PGIDVNAR**[1]	2	1	0.01	0.07	0	0	1.00	1.00
**TPLHLAV**[1]	0	0	1.00	1.00	1	3	0.17	1.00
**CCSQYGWC**[2]	0	0	1.00	1.00	0	0	1.00	1.00
**GIDVNARD**[1]	1	0	0.06	0.18	0	0	1.00	1.00
**I[AM]STKRTW**	2	1	0.01	0.07	0	0	1.00	1.00

[1] motif part of ankyrin domain proteins
[2] motif part of CBM18 domain

Supplementary Table S9. List of PL^{eff}, H^{eff} and L^{eff} effector candidates in *P. brassicae*.

Gene ID	Pfam ID	Pfam description	Blast hit accession	Blast_hit_description	e-value	*blast hit species*	*S. subterranea* match	Peptide*
**PL^{eff}**								
**0030**	PF00098	Zinc knuckle	YP_004061645.1	hypothetical protein OlV1_012c	5,14E-12	*Ostreococcus lucimarinus virus OlV1*	m.11712	
0043	-	-	-	-	-	-		
0190	-	-	-	-	-	-		
**0499**	PF13639	Ring finger domain	XP_002441802.1	hypothetical protein SORBIDRAFT_08g002550	4,25E-06	*Sorghum bicolor*		
0573	-	-	-	-	-	-		
0634	PF00561;PF04083	alpha/beta hydrolase fold; alpha/beta−hydrolase lipase region	XP_006984073.1	PREDICTED: lysosomal acid lipase/cholesteryl ester hydrolase	1,24E-90	*Peromyscus maniculatus bairdii*		
1204	-	-	-	-	-	-		
1454	-	-	-	-	-	-		
1477	-	-	-	-	-	-		
1856	-	-	-	-	-	-		P
**2462**	-	-	-	-	-	-		G,P,M
2550	-	-	-	-	-	-		
3268	-	-	XP_002153500.1	hypothetical protein PMAA_013780	7,36E-15	*Talaromyces marneffei ATCC 18224*		
3405	PF12796	Ankyrin repeats (3 copies)	EJT69134.1	hypothetical protein GGTG3243	9,20E-22	*Gaeumannomyces graminis var. tritici R3-111a-1*		
4539	-	-	-	-	-	-		
4950	-	-	-	-	-	-		
5027	PF13855	Leucine rich repeat	XP_005833696.1	hypothetical protein GUITHDRAFT07493	4,94E-26	*Guillardia theta CCMP2712*		
**5439**	-	-	-	-	-	-		
5498	-	-	-	-	-	-		P
5499	-	-	-	-	-	-		P,M
5609	-	-	CBQ73209.1	conserved hypothetical protein	8,08E-16	*Sporisorium reilianum SRZ2*		
5941	-	-	-	-	-	-	m.41093	
6216	PF00156	Phosphoribosyl transferase domain	WP_003374851.1	hypoxanthine phosphoribosyltransferase	2,51E-48	*Clostridium botulinum*		
6649	PF12796	Ankyrin repeats (3 copies)	4HQDA	Chain A, Crystal Structure Of Engineered Protein	8,62E-17	synthetic construct		
6655	PF12796	Ankyrin repeats (3 copies)	WP_007552875.1	ankyrin repeat domain protein, partial	5,28E-16	*Wolbachia* endosymbiont of		

						Drosophila ananassae		
6676	PF00069	Protein kinase domain	EGU12202.1	putative Serine/threonine protein kinase	4,32E-31	*Rhodotorula glutinis ATCC 204091*		
6871	PF12796	Ankyrin repeats (3 copies)	CCD48333.1	putative nacht and ankyrin domain protein	4,41E-24	*Botryotinia fuckeliana T4*		
6928	PF00190	Cupin	CBN75540.1	cupin-like protein	2,95E-29	*Ectocarpus siliculosus*		
7419	-	-	-	-	-	-		
7521	PF13637	Ankyrin repeats (many copies) -	XP_007799856.1	hypothetical protein EPUS_03910	1,24E-08	*Endocarpon pusillum Z07020*		
7566	-	-	-	-	-	-		
7592	-	-	-	-	-	-		
7749	PF12796	Ankyrin repeats (3 copies)	EGN97633.1	hypothetical protein SERLA73DRAFT_30563	1,24E-11	*Serpula lacrymans var. lacrymans S7.3*		
7764	PF02493	MORN repeat	WP_026757912.1	2-isopropylmalate synthase	2,09E-16	*Sediminimonas qiaohouensis*		
7977	PF13637;PF12796;PF00023	Ankyrin repeats (many copies);Ankyrin repeats (3 copies);Ankyrin repeat	WP_015589078.1	Ankyrin repeat domain protein	4,35E-32	*Wolbachia endosymbiont of Drosophila simulans*		G,M
8088	PF13306;PF13855	Leucine rich repeats (6 copies);Leucine rich repeat	4PSJA	Chain A, Crystal Structure Of Engineered Protein	4,43E-24	synthetic construct		
8439	PF12796	Ankyrin repeats (3 copies)	WP_015589078.1	Ankyrin repeat domain protein	2,26E-20	*Wolbachia* endosymbiont of *Drosophila simulans*		
8556	PF00023;PF12796	Ankyrin repeat;Ankyrin repeats (3 copies)	CDG49342.1	Ankyrin repeats-containing protein	3,24E-21	*Cardinium* endosymbiont cBtQ1 of *Bemisia tabaci*		
8608	-	-	-	-	-	-		
8788	-	-	-	-	-	-	m.29468	
8843	PF03490	Variant−surface−glycoprotein phospholipase C	KDO31622.1	hypothetical protein SPRG_03542	1,26E-49	*Saprolegnia parasitica*		
8885	PF12796;PF13637	Ankyrin repeats (3 copies);Ankyrin repeats (many copies)	CCD52555.1	hypothetical protein BofuT4_P000020.1	2,05E-20	*Botryotinia fuckeliana T4*		
9320	PF13855	Leucine rich repeat	XP_004342824.1	predicted protein	1,14E-35	*Capsaspora owczarzaki ATCC 30864*		
9634	PF12796	Ankyrin repeats (3 copies)	CAK40071.1	unnamed protein product	1,13E-06	*Aspergillus niger*		M
**H^{eff}**								
0023	PF14520;PF08423	Helix−hairpin−helix domain;Rad51	XP_002126934.1	PREDICTED: D - repair protein RAD51 homolog 1-like isoform 1	1,36E-164	*Ciona intestinalis*	m.9287	
0207	PF00128;PF07821	Alpha- amylase, catalytic domain;Alpha−amylase C−terminal beta−sheet domain	XP_005839103.1	hypothetical protein GUITHDRAFT02025	2,92E-85	*Guillardia theta CCMP2712*		G,M
0444	PF03492	SAM dependent carboxyl methyltransferase	AFK13134.1	PbBSMT benzoic acid/salicylic acid methyltransferase	0	*Plasmodiophora brassicae*	m.41246	M
0733	-	-	-	-	-	-		

**1932**	-	-	-	-	-	-		
2230	PF01522	Polysaccharide deacetylase	CAM98715.1	hypothetical protein	0	*Plasmodiophora brassicae*		
**2543**	PF00187	Chitin recognition protein	XP_002840637.1	hypothetical protein	4,13E-12	*Tuber melanosporum Mel28*	m.4029	
**2565**	PF02221	ML domain	XP_007861078.1	Phosphatidylglycerol/phosphatidylinositol transfer protein	4,51E-09	*Gloeophyllum trabeum*		
3156	PF00722	Glycosyl hydrolases family 16	XP_003388464.1	PREDICTED: beta-1,3-glucan-binding protein-like	1,28E-115	*Amphimedon queenslandica*	m.16803	G
**3213**	-	-	XP_004338485.1	hypothetical protein ACA1_034100	1,67E-10	*Acanthamoeba castellanii str. Neff*	m.37585	
3270	-	-	-	-	-	-	m.35192	
3303	-	-	-	-	-	-		G
5075	-	-	-	-	-	-	m.14623	
5624	-	-	XP_758973.1	hypothetical protein UM02826.1	1,01E-18	*Ustilago maydis 521*		G,P,M
6104	-	-	-	-	-	-	m.38074	
6244	-	- -	-	-	-	-		
6312	PF00026	Eukaryotic aspartyl protease	AEI58895.1	cathepsin D	7,81E-111	*Pteria penguin*	m.37017	
**6677**	PF00187	Chitin recognition protein	-	-	-	-	m.19486	
7823	-	-	-	-	-	-		
7865	PF00069	Protein kinase domain	XP_004994481.1	CMGC/DYRK/DYRK1 protein kinase	2,13E-36	*Salpingoeca rosetta*		
7941	-	-	-	-	-	-		
8221	-	-	-	-	-	-		
8941	PF11051	Mannosyltransferase putative	EPB90016.1	hypothetical protein HMPREF1544_03128	4,54E-39	*Mucor circinelloides f. circinelloides 1006PhL*		
**8942**	PF00187 (2x); PF01522	Chitin recognition protein;Chitin recognition protein;Polysaccharide deacetylase	CAM98715.1	hypothetical protein	8,04E-47	*Plasmodiophora brassicae*		G,M
9298	-	-	-	-	-	-		
**L^{eff}**								
**1295**	PF01522	Polysaccharide deacetylase	CAM98715.1	hypothetical protein	1,46E-41	*Plasmodiophora brassicae*		
**1430**	PF07648	Kazal−type serine protease inhibitor domain	XP_002953335.1	hypothetical protein VOLCADRAFT05857	5,77E-27	*Volvox carteri f. - gariensis*		
1436	PF02469	Fasciclin domain	WP_013825348.1	beta-Ig-H3/fasciclin	1,92E-09	*Methanobacterium sp. SWAN-1*		G,M
1998	-	-	-	-	-	-		
2061	-	-	-	-	-	-		
2200	-	-	-	-	-	-	m.13032	

3357	-	-	-	-	-	-	m.6606	G,M
3510	-	-	-	-	-	-	m.40594	G,M
3906	PF00112;PF08246	Papain family cysteine protease;Cathepsin propeptide inhibitor domain	ABV22332.1	cysteine protease 1	1,93E-57	*Noctiluca scintillans*		
**4045**	PF02140	Galactose binding lectin domain	XP_002600445.1	hypothetical protein BRAFLDRAFT09205	2,27E-17	*Branchiostoma floridae*	m.11135	
4237	-	-	-	-	-	-		G
5421	-	-	-	-	-	-	m.38972	G
5577	-	-	-	-	-	-		G
6101	PF02469	Fasciclin domain	WP_023001352.1	MULTISPECIES: Nex18 symbiotically induced protein	7,96E-10	*Labrenzia*	m.5199	G
6634	-	-	-	-	-	-		
6681	PF00026	Eukaryotic aspartyl protease	XP_002108510.1	expressed hypothetical protein	1,18E-109	*Trichoplax adhaerens*		G,M
6988	PF05257	CHAP domain	ETV90925.1	hypothetical protein H3104411	1,11E-54	*Aphanomyces invadans*		
7575	-	-	-	-	-	-		
7623	-	-	WP_011021727.1	hypothetical protein	9,65E-09	*Methanosarci acetivorans*		
8176	PF02265	S1/P1 Nuclease	AAD00695.1	bifunctional nuclease	2,85E-39	*Zinnia violacea*		
8630	PF02469	Fasciclin domain	WP_014405979.1	Secreted and surface protein	2,06E-09	*Methanocella conradii*		G
9391	-	-	-	-	-	-		G,M
9670	-	-	-	-	-	-		

*Protein detected in germinating spores (G), plasmodia (P) or maturing spores (M)

Bold numbers indicate cysteine-rich proteins (>3%)

Supplementary Table S10. Chitin response to *P. brassicae* infection. Compilation of fold-change difference in transcript levels of Arabidopsis LysM-domain receptor genes[21] of a microarray assay[22] 10 and 23 days post inoculation (dpi) with *P. brassicae.* Fold-change differences between mock and inoculated materials at values less or higher than 2 are in bold.

**Gene**	**Locus**	**Fold-change**	
		10 dpi	23 dpi
AtCERK1	At3g21630	-1.20	-1.41
AtLYK2	At3g01840	1.62	1.42
AtLYK3	At1g51940	1.77	1.04
AtLYK4	At2g23770	**-4.55**	**-7.14**
AtLYK5	At2g33580	1.40	1.02
AtLYP1	At2g17120	-1.35	-1.12
AtLYP2	At1g21880	1.43	**-2.33**
AtLYP3	At1g77630	1.16	**-2.38**

Supplementary Note

Resting spore purification

The *P. brassicae* single spore isolate e3[23] was used in all analysis in this work in order to establish a reference genome. Clubroots of *Brassica napus* infected with *P. brassicae* collected and frozen since 2001 were washed and surface sterilized[24]. Galls were homogenized in sterile H_2O using a household mixer, the solution filtered through 8 layers of cheesecloth to remove plant cell debris followed by centrifugation for 5 min at 1000g. The supernatant was transferred to new tubes, washed with sterile H_2O followed by a two-step gradient centrifugation using 16 and 32% Ficoll. The floating spores were harvested, washed with sterile H_2O and treated with rifampicin and streptomycin (100 µg/ml) for 1 h at 37°C, washed in sterile H_2O and pelleted. A second step with surface sterilization and antibiotic treatments was performed using 10 % chloraminT (Sigma-Aldrich) and 70% ethanol, followed by a antibiotic multi-treatment (100µg/ml carbenicillin, 100 µg/ml pimaricin, 400 µg/ml timentin, 250 µg/ml cefotaxim, 100 µg/ml vancomycin and 50 µg/ml hygromycin B) overnight at room temperature[25]. The spores were then washed and incubated in a 4 mg/ml lysozyme solution (Sigma-Aldrich) for 2h at 37°C to lyse contaminating bacteria, pelleted and resolved in 3 ml PBS buffer followed by a gradient centrifugation. The Optiprep reagent (Sigma-Aldrich) in DNA extraction buffer[26] was used with following concentrations and volumes: 50%: 2ml, 40%: 2ml, 30%: 2ml, 20 %: 4ml, 10%: 1ml. Spores from the 30-40% phase were collected, washed in sterile H_2O, pelleted, resolved in 2ml TE buffer with 10U DNase I (Fermentas), and incubated at 37°C for 2h. The DNase I treatment was terminated by adding proteinase K for 4h at 37°C. The spores were finally washed 3 times in sterile H_2O, centrifuged and snap frozen in liquid nitrogen, and stored at -20°C until DNA or RNA extraction.

DNA purity tests

After DNA extraction the presence of contaminating DNA was tested by PCR using *Brassica* specific actin primers, fw: 5'–GGTAGGCCAAGACATCACGGTGTCATGG–3' and rev: (5'–TGTACTTCCTCTC GGGAGGTGCAACC–3') and following conditions: 95°C for 3 min followed by 30 cycles of 95°C for 30 sec, 61°C for 30 sec, 72°C for 45 sec and a final step of 72°C for 5 min. Fungal DNA contamination was analyzed using the fungal primers TR1 and TR2[27] with an annealing temperature of 52 °C. Under the conditions tested no products were obtained with either primer pairs suggesting that the gDNA used for the genome sequencing was almost entirely of *P. brassicae* origin.

Transcriptome samples and RNA extractions

Total RNA was obtained from surface sterilized clubroots of *B. rapa* cv. Granaat (ECD5), *B. napus* Dc119 Giant rape (ECD7), and *B. oleracea* var. *capitata* Jersey Queen (ECD13) 5 weeks post infection (ECD numbers according to[28]). RNA from germinating spores was obtained from purified resting spores stored at -20°C. The spores were thawed at 4°C for 48h followed by 24h at room temperature in

sterile H_2O with 400ng/ml timentin to inhibit bacterial contamination. Samples were pelleted, snap frozen and stored at -80°C prior RNA extraction. RNA from maturing resting spores were obtained from resting spores directly after purification from 5 week-old *B. rapa* cv Granaat clubs. Plasmodia were isolated from *B. napus* cv. Westar clubroots, 5 weeks after infection, following a physical disruption method[29]. Plasmodia were carefully removed from the centrifuge tube wall, microscopically checked for plant and spore contamination. Plasmodia were pelleted 5 min at 200g and snap frozen in liquid nitrogen before RNA extraction or lypholization for proteome analyzes. The data from the proteome analysis were also used to check plant contamination in the plasmodial RNA sample. In addition, potato root galls incited by *Spongospora subterranea* from potatoes grown for 2 months in natural infested soil were used as RNA source. All RNA extractions were performed using the Spectrum™ Plant Total RNA Kit (Sigma-Aldrich) according to the manufacturers instructions.

Sample preparation for proteomic analyses

Aliquots of the plasmodia sample and resting spore solutions (cell density $2x10^9$ /ml) were used for the proteome analyses. To obtain germinating spores, the resting spores were incubated on a shaker at room temperature for 3 days to obtain germinated spores[30]. 50 µl aliquots of the spore samples were homogenized by three liquid nitrogen freeze/ thaw cycles followed by sonication lypholized. All samples were resolved in 50 µl of 65 mM Tris HCl buffer (pH 6.8) containing 2% SDS and sonicated for 45 min before loading electrophoresis[31] using 1 mm 12% polyacrylamide gels (BioRad, Munich, Germany). Gels were stained with Coomassie Brilliant Blue and destained in 50% aqueous methanol in 5% acetic acid. Each lane cut into 5-9 slices was digested with trypsin[32]. The recovered peptides were dissolved in 15 µl of 5% aqueous formic acid and 5 µl were analyzed by LC–MS/MS on an Ultimate3000 nanoLC system (Dionex, Amsterdam, The Netherlands) equipped with a 75 µm i.d. × 20 mm trap column and 75 µm × 15 cm analytical column (Acclaim PepMap100 C18 3 µm/100A, Dionex) interfaced on-line to a LTQ Orbitrap Velos hybrid tandem mass spectrometer (Thermo Fisher Scientific, Bremen, Germany). Linear gradient of 0 to 30% of acetonitrile in 0.5 % aqueous formic acid / acetonitrile (Merk, Darmstadt, Germany) was delivered in 120 min followed by successive washing with 100% acetonitrile and 100% 0.5% formic acid. The data-dependent acquisition (DDA) cycle consisted of FT MS survey scan with the targeted mass resolution R m/z 400 = 60000 followed by 6 MS/MS spectra acquired at the linear ion trap. FT MS survey scans were acquired within m/z range of 350 to 1600; CID MS/ MS spectra at nCE of 35 eV%.

Protein identification

MS/MS spectra were converted to mgf format using extract_msn converter (ver.5, Thermo Fisher Scientific, Bremen, Germany) and mgf files produced by the LC-MS/MS analyses of individual gel slabs were merged. Proteins were identified by MASCOT v.2.2.04 software (Matrix Sciences) by searching against a protein sequence database under the following settings: 5 ppm and 0.5 Da mass

accuracy for precursor and fragment ions, respectively; enzyme specificity: trypsin; number of allowed miscleavage sites: two; variable modifications: methionine oxidized, cysteine propionamide, asparagine and glutamine deamidated. The database consisted of: protein sequences from *P. brassicae*, human keratins, trypsin and *Brassica* ssp sequences (total of 184351 protein sequences). Host plant proteins contaminants ranged from 69 proteins in plasmodia to 790 proteins in resting spores. Scaffold software (Proteome Software, Inc.) was used for statistical evaluation of protein hits and proteins with > 99.0% protein probability (FDR <0.1), 95.0% peptide probability (FDR 0.9%) and the minimum of 2 matched peptides were accepted as *P. brassicae* proteins.

Genome sequencing

The genome of the *P. brassicae* was determined by combining 454 Roche and Illumina sequencing. Two libraries were sequenced using 11µg gDNA and Roche 454 technology (Macrogen Korea). A standard library consisting of 713,039 reads of an average read length of 776 bp and approximately 20-fold genome sequence coverage was obtained. The second library was based on 57,172 mate pair reads with a 3kb insert size with an average read length of 414 bp, and an approximately 0.8 fold genome sequence coverage. Additionally, two paired-end libraries were constructed and Illumina (HiSeq 2100) sequenced. The library (prepared from 5 µg gDNA) sequenced at by SciLife Lab (Stockholm Sweden) consisted of 196 million paired-end reads (read length of 101bp and an insert size of 255 bp) had an approximately 600-fold genome sequence coverage. A 5kb insert Illumina library, prepared from 20 µg gDNA amplified with the repliG-DNA kit (Qiagen) resulted in 7.0 million mate-paired reads (read length 90bp, approximately 20-fold coverage) and was sequenced at BGI Hong Kong, China. Illumina adaptor sequences were removed from the reads using Cutadapt v2.2[33], and quality filtered using Quake v0.3.4[34] prior to merging the sequence data.

Merging Illumina and Roche assemblies

A first draft genome assembly of *P. brassicae* from the 454 reads using Newbler v2.9[35] were done with default settings. Homopolymer errors were detected by the Nesoni pipeline v0.109 (Victorian Bioinformatics Consortium). Illumina insert sizes were estimated by mapping the reads to the assembly using BWA v0.7.5[36] and the Picard tool CollectInsertSizeMetrics v1.96 (http://picard.sourceforge.net). The assembly was further scaffolded using all Illumina genomic reads using SSPACE v2.2[37]. The paired-end Illumina data was used to fill assembly gaps with GapFiller v1.11[38]. The average read coverage was assessed by mapping the reads to the assembly using BWA v0.7.5 for Illumina reads and Newbler v2.9 for the 454 reads and the depth was recorded using Samtools v 0.19[39], using default setting in all cases. The final assembly was subjected to BLASTN searches (e-value $<10^{-9}$) against the genome sequences of *Arabidopsis thaliana* (TAIR 9, www.arabidopsis.org) and *Brassica rapa*[40]. Scaffolds with a Illumina coverage lower than 110-fold were regarded as contamination and were removed from the assembly as scaffolds with blast hits to predominantly to plant sequences never

reached a higher coverage. The removed scaffolds were subjected to a BLASTN search against NCBI nt database (e-value<10^{-9}), all scaffolds had the best hit to either *Brassica* spp. or *Arabidopsis* spp. Additionally, two scaffolds with a higher coverage were removed as they were almost identical to the *B. rapa* chloroplast and mitochondria.

Transcriptome sequencing

All RNA samples were sequenced with Illumina sequencing technology (HiSeq 2100) to characterize the transcriptomes of *P. brassicae* (Supplementary Table S3). Libraries were prepared by the sequencing platforms under their in-house conditions. Illumina adaptor sequences were removed using Cutadapt v2.2[33] Low-quality reads were filtered using Condetri v.2.2[41]. The transcriptome libraries of *P. brassicae* and *S. subterranea* were *de novo* assembled using Trinity v20130814[42] with default settings except for the jaccad_clip. For *P. brassicae*, the strand-specific libraries were mapped to the genome assembly using Tophat v2.0.9[43] and analyzed using Cufflinks v 2.1.1[44], with default settings and an expected intron length of 5 to 5kb. For *S. subterranea* potato host transcripts were removed from the subsequent analyses, if the assembled transcripts had a BLASTN match (e-value<10^{9}) to the *Solanum tuberosum* genome[45] or *A. thaliana* (TAIR 9, www.arabidopsis.org), and no BLASTN match to the *P. brassicae* assembly at an e-value <10^{-9}. Transcripts with BLASTN matches to the potential contaminant Potato virus A/Y (11 transcripts), and transcripts masked as retroelement (1288 transcripts) or transposons (251 transcripts) using RepeatMasker v4.0.3 (http://www.repeatmasker.org) and Repbase[46] were removed. Only transcripts coding for peptides of minimal length of 100 amino acids were subsequently analyzed.

Gene prediction

Ab initio predictions were received from AUGUSTUS v2.5.5[47], SNAP[46] and GeneMark-ES v2.3[49]. tBLASTX hits (e-value <10^{-10}) to the UniProt/Swissprot database, Rhizarian ESTs and proteins from NCBI and transcripts inferred from Cufflinks (using the strand-specific RNA-sequence libraries generated from *P. brassicae*) were used as evidence. All received data above were combined using MAKER v2.31[50] to predict *P. brassicae* genes. Maker was configured to use both spliced EST alignments and single ESTs longer than 250 bp as evidence to predict hint-based genes. To minimize fusion of nearby genes the pred_flank parameter was lowered to 100 bp and split_hit to 100 bp (expected maximum intron size). All *ab initio* predictors in the Maker pipeline, except GeneMark-ES, were trained with CEGMA set. The obtained gene models with an AED score < 0.5 and a subset of manually annotated gene models were used to retrain the *ab initio* predictors before rerunning the MAKER pipeline.

All predicted gene models were manually checked and if necessary adjusted using Apollo[51]. The correction included the addition of missing exons, shortening of predicted coding sequences by identifying later start codons, and splitting predicted gene models into two or more individual models

based on transcript evidence. Gene models with no transcriptional evidence and no BLAST evidence were removed. All predicted gene models were scanned for protein signatures using Interproscan[52] and matched to the NCBI database nr using BLASTP[53], e-value<10^{-5}. Gene IDs were assigned on the basis of their location on the scaffolds.

Functional gene annotation

Proteins family classifications for the *P. brassicae* and *S. subterranea* gene models were inferred using Interproscan[50]. InterproScan conducted searches against Phobius v1.01[54] to detect signal peptides and transmembrane domains, and against multiple protein sequence and protein domain databases (Pfam v27.0[55], SMART v6.2[5], Gene3D v3.5.0[56], PANTHER v8.1[57], SUPERFAMILY v1.75[58], PRINTS v42.0[59], ProSiteProfiles v20.89 and ProSitePatterns[60]. GO-terms were assigned using Blast2GO v2.5[61] by combining BLAST-based predictions (top ten hits of BLASTP (e-value 10^{-5}) against the NCBI database) and the Interproscan derived protein signatures.

Overrepresentation of Pfam domains and amino acid motifs

Protein models of fungi, omycetes and Rhizaria were scanned for Pfam domains using hmmscan v 3.1 (www.hmmer.org). Enrichment of Pfam domains in the set of predicted secretome was calculated as outlined by[62]. Pfam domains for each organism/tree node (Supplementary Fig. S7) were compared to the Pfam domains identified in the total set of organisms. The probability of observing these domains was calculated using the cumulative hyperbolic distribution. P-values were adjusted for multiple testing using Bejamini and Hochberg correction[63]. Over-represented peptide motifs in the predicted secretome were identified using MEME v4.9.0[64] searching for motifs with a length of 4-10 amino acids. Enrichment of known pathogenicity related motifs and the *de novo* motifs in the predicted secretome compared to the non-secreted proteins were calculated, p-values were estimated using cumulative hypergeometric distribution. P-values were adjusted for multiple testing[63].

Cloning and recombinant expression of the PbGH3 gene in *E. coli*

The *PbGH3* gene was cloned in the expression vector pGEX 4T3 (Amersham Bioscience) to give a glutathione S-transferase (GST) fusion protein[65]. The expression of the GST::PbGH3 fusion protein in *E. coli* BL21 (DE) codon plus cells was induced with 1 mM isopropyl β-d-thiogalactoside (IPTG) for 3 hr at 22 °C. Subsequently the cells were collected and washed in TB-buffer (9.1 mM HEPES, 55 mM MgCl2, 15 mM CaCl2, 250 mM KCl, pH 6.7). To lyse the cells, they were resuspended in lysis buffer (1xPBS buffer with 1 mg/ml lysozyme, 10 mM MgCl2, 10 U/ml DNase1) and subjected to three freeze/thaw cycles (30 s liquid nitrogen/10 min 37 °C). After centrifugation (10 min; 13000 x g) the purification of GST::PbGH3 proteins was done using Glutathione Sepharose 4B (Amersham) according to the manufacturer´s protocol. Quality and quantity of the eluted fusion protein were verified using 12 % SDS-PAGE minigels. As controls *E. coli* cells transformed with empty vector were used.

Conjugate synthetase test

The auxins indole-3-acetic acid (IAA), indole-3-butyric acid (IBA) and indole-3-propionic acid (IPA) and jasmonic acid (1 mM each) were used as substrates for the activity tests. The enzyme assays[66] were run for 12 hr at room temperature. Each assay contained 10 µg purified GST::PbGH3 fusion protein in 20 µl of 50 mM Tris-HCl, pH 8.6; 3 mM MgCl2; 3 mM ATP; 1 mM DTT and 1 mM of each amino acid. The whole reaction were spotted on silica gel 60 F254 plates (Merck KGaA) and developed in chloroform:ethylacetate:formic acid (35:55:10; v/v) with the exception of the IAA-His, -Ser, -Thr, -Arg, -Lys and -Cys conjugates which were developed in 2-propanol:ammonium hydroxide:H2O (8:1:1, v/v). Indoles were stained with van urk-Salkowski Reagent[67], whereas jasmonates were stained with Vanillin reagent[68]. Salicylic acid (SA) and putatively formed conjugates[69] were detected using dichloromethane:ethyl acetate:formic acid (3:6:1), and for putative amino acid conjugates of SA formed with His, Ser, Thr, Arg, Lys, and Cys the same solvent as form indoles and jasmonates was used. Staining was performed with 1% Fe(III)chloride in 50% methanol.

Alignment and phylogenetic analyses of the CHS domains

A de novo alignment guided with structural information was built for a set of representative sequences with T-Coffee Expresso[6]. This alignment was converted into a profile and all chitin synthase sequences were aligned to it using HMMER[70]. The final alignment was trimmed and manually edited with SeaView[71]. The Bayesian phylogeny was reconstructed using MrBayes v3.2.1[4]. The analysis was run for 150,000 generations, when the Potential Scale Reduction Factor reached 1.017 and 99% of the credible set of trees contained 1015 out of the 1060 trees sampled. The Maximum Likelihood (ML) phylogeny was reconstructed with RaxML v8.0.20[72]. The best-known tree was selected among 100 independently run trees (-f d -m PROTGAMMALG -# 100). To assess node support, 500 replicates of the original alignment were produced, and their corresponding trees were mapped into node support values on to the best-known tree. The protein substitution matrix used was Le-Gascuel[73], as calculated by ModelGenerator[74]. The phylogenies recovered by both methods were consistently robust regarding the sublineage/family bifurcations, and minor differences were only found between the order of tripartite clades towards the tips where the variability is insufficient to distinguish between orthologous sequences from closely related strains.

Supplementary References

1. Parra, G., Gradnam, K. & Korf, I. CEGMA: a pipeline to accurately annotate core genes in eukaryotic genomes. *Bioinformatics* **23**, 1061-1067 (2007).
2. Edgar, R.C. MUSCLE: multiple sequence alignment with high accuracy and high throughput. *Nucleic Acids Res.* **32**,792–1797 (2004).
3. Castresana, J. Selection of conserved blocks from multiple alignments for their use in phylogenetic analysis. *Mol. Biol. Evol.* **17**, 540–552 (2000).
4. Ronquist, F., Huelsenbeck, J. & Teslenko, M. Draft MrBayes version 3.2 Manual: Tutorials and Model Summaries. http://mrbayes.sourceforge.net/ mb3.2_manual.pdf (2000).
5. Letunic, I., Doerks, T. & Bork, P. (SMART: recent updates, new developments and status in 2015. *Nucleic Acids Res.* gku949 (2014).
6. Notredame, C., Higgins, D.G. & Heringa, J. T-Coffee: a novel method for fast and accurate multiple sequence alignment. *J. Mol. Biol.* **302**, 205-217 (2000).
7. Arnold, K., Bordoli, L., Kopp, J. & Schwede, T. The SWISS-MODEL workspace: a web-based environment for protein structure homology modelling. *Bioinformatics* **22**, 195–201 (2006).
8. Pettersen, E.F. *et al.* UCSF chimera—a visualization system for exploratory research and analysis. *J Comput. Chem.* **25**, 1605–1612 (2004).
9. Lindner, A.C. *et al.* Isopentenyltransferase-1 (IPT1) knockout in *Physcomitrella* together with phylogenetic analyses of IPTs provide insights into evolution of plant cytokinin biosynthesis. *J. Exp. Bot.* **65**, 2533-2543 (2014).
10. Jones, D.T., Taylor, W.R. & Thornton, J.M. The rapid generation of mutation data matrices from protein sequences. *Computer Appl. Biosc.* **8**, 275-282 (1992).
11. Li, L., Stoeckert, C.J.Jr. & Roos, D.S. OrthoMCL: identification of ortholog groups for eukaryotic genomes. *Genome Res.* **13**, 2178–2189 (2003).
12. Lombard, V., Golaconda Ramulu, H., Drula, E., Coutinho, P.M. & Henrissat, B. The carbohydrate-active enzymes database (CAZy) in 2013. *Nucleic Acids Res.* **42**, D490–495 (2014).
13. Aspeborg, H., Coutinho, P.M., Wang, Y., Brumer, H. & Henrissat, B. Evolution, substrate specificity and subfamily classification of glycoside hydrolase family 6 (GH5). *BMC Evol. Biol.* **12**,186 (2012).
14. Karlsson, M. & Stenlid, J. Evolution of family 18 glycoside hydrolases: diversity, domain structures and phylogenetic relationships. *J. Mol. Microbiol. Biotechno.l* **16**, 208–223 (2009).
15. Larkin, M.A. *et al.* Clustal W and Clustal X version 2.0. *Bioinformatics* **23**, 2947-2948 (2007).
16. Müller, P. & Hilgenberg, W. Isomers of zeatin and zeatin riboside in clubroot tissue: evidence for trans-zeatin biosynthesis by *Plasmodiophora brassicae*. *Physiol. Plant*. **66**, 245-250 (1986).
17. Iriti, M. & Faoro, F. Chitosan as a MAMP, searching for a PRR. *Plant Signal Behav.* **4**, 66-68 (2009).
18. Sundelin, T. *et al.* In planta quantification of *Plasmodiophora brassicae* using signature fatty acids and real-time PCR. *Plant Dis.* **94**, 432-438 (2010).
19. Elbein, A.D., Pan, Y.T., Pastuszak, I. & Carroll, D. New insights on trehalose: a multifunctional molecule. *Glycobiology* **13**, 17R-27R (2003).
20. Wilson, R.A., Gibson, R.P., Quispe, C.F., Littlechild, J.A. & Talbot, N.J. An NADPH-dependent genetic switch regulates plant infection by the rice blast fungus. *Proc. Natl. Acad. Sci. USA* **107**, 21902–21907 (2010).
21. Tanaka, K., Nguyen, C.T., Liang, Y., Cao, Y. & Stacey, G. Role of LysM receptors in chitin-triggered plant innate immunity. *Plant Signal Behav.*, **8**, e22598 (2013)..
22. Siemens, J. *et al.* Transcriptome analysis of *Arabidopsis* clubroots indicates a key role for cytokinins in disease development. *Mol. Plant-Microbe Interact.* **19,** 480–494 (2006).
23. Fähling, M., Graf, H. & Siemens, J. Characterization of a single-spore isolate population of *Plasmodiophora brassicae* resulting from a single club. *J. Phytopathol.* **152**,438–444 (2004).
24. Bulman, S. *et al.* Genomics of biotrophic, plant-infecting plasmodiophorids using in vitro dual cultures. *Protist* **162**,449–461 (2011).
25. Asano, T., Kageyama, K. & Hyakumachi, M. Germination of surface-disinfected resting spores of *Plasmodiophora brassicae* and their root hair infection in turnip hairy roots. *Mycoscience* **41**,49-54 (2000).
26. Russel, J. & Bulman, S. The liverwort *Marchantia foliacea* forms a specialized symbiosis with arbuscular mycorrhizal fungi in the genus *Glomus*. *New Phytol.* **165**,567–579 (2005).
27. Bock, M., Maiwald, M., Kappe, R., Nickel, P. & Näher, H. Polymerase chain reaction-based detection of dermatophyte DNA with a fungus-specific primer system. *Mycoses* **37**,79-84 (1994).
28. Buczacki, S.T. *et al.* Study of physiologic specialization in *Plasmodiophora brassicae*: Proposals for attempted rationalization through an international approach. *Trans. British Myc. Soc.* **65**, 295-303 (1975).
29. Dekhuijzen, H.M. The enzymatic isolation of secondary vegetative plasmodia of *Plasmodiophora brassicae* from callus tissue of *Brassica campestris. Physiol. Plant Pathol.* **6,** 187–192 (1975).

30. Niwa, R., Nomura, Y., Osaki, M. & Ezawa, T. Suppression of clubroot disease under neutral pH caused by inhibition of spore germination of *Plasmodiophora brassicae* in the rhizosphere. *Plant Pathol.* **57**, 445–452 (2008).
31. Knaust, A., Shevchenko. A. & Shevchenko, A. Horizontal carryover of proteins on one-dimensional polyacrylamide gels may jeopardize gel-enhanced liquid chromatography mass spectrometry proteomic interpretations. *Anal. Biochem.* **421**, 779–781 (2012).
32. Shevchenko, A., Tomas, H., Havlis, J., Olsen, J.V. & Mann, M. In-gel digestion for mass spectrometric characterization of proteins and proteomes. *Nat. Protoc.* **1**, 2856–2860 (2006).
33. Martin, M. Cutadapt removes adapter sequences from high-throughput sequencing reads. *EmboNet J* **17**, 10-12 (2011).
34. Kelly, D.R., Schatz, M.C. & Salzberg, S.L. Quake: quality-aware detection and correction of sequencing errors. *Genome Biol.* **11**, R116 (2010).
35. Wijaya, E., Frith, M.C., Suzuki, Y. & Horton, P. Recount: expectation maximization based error correction tool for next generation sequencing data. *Genome Inform.* **23**,189–201 (2009).
36. Li, H. & Durbin, R. Fast and accurate short read alignment with Burrows-Wheeler transform. *Bioinformatics* **25**, 1754-1760 (2009).
37. Boetzer, M., Henkel, C.V., Jansen, H.J., Butler D. & Pirovano, W. Scaffolding pre-assembled contigs using SSPACE. *Bioinformatics* **27**, 578–579 (2011).
38. Boetzer, M. & Pirovano, W. Toward almost closed genomes with GapFiller. *Genome Biol.* **13**, R56 (2012).
39. Li, H. *et al.* The sequence alignment/map (SAM) format and SAMtools. *Bioinformatics* **25**, 2078-2109 (2009).
40. Wang, X. *et al.* The genome of the mesopolyploid crop species *Brassica rapa*. *Nature Genet* **43**, 1035–1039 (2011).
41. Smeds, L. & Künstner, A. ConDeTri– a content dependent read trimmer for Illumina data. *PLoS One* **6**, e26314 (2011).
42. Grabherr, M. *et al.* Full-length transcriptome assembly from RNA-Seq data without a reference genome. *Nat. Biotechnol*. **29**, 644-652 (2011).
43. Trapnell, C., Pachter, L. & Salzberg, S.L. Tophat: discovering splice junctions with RNA-seq. *Bioinformatics* **25**, 1105–1111 (2009).
44. Trapnell, C. *et al*. Transcript assembly and quantification by RNA-Seq reveals unannotated transcripts and isoform switching during cell differentiation. *Nat. Biotechnol.* **28**, 511–515 (2010).
45. The Potato Genome Sequencing Consortium (TPGSC) Genome sequence and analysis of the tuber crop potato. *Nature* **475**, 189-197 (2011).
46. Jurka, J. *et al*. (2005) Repbase Update, a database of eukaryotic repetitive elements. *Cytogenet. Genome Re.s* **110**, 462–467 (2005).
47. Stanke, M., Steinkamp, R., Waack, S. & Morgenstern, B. AUGUSTUS: a web server for gene finding in eukaryotes. *Nucleic Acids Res.* **32**,W309–312 (2004).
48. Korf, I. Gene finding in novel genomes. *BMC Bioinf.* **5**,59 (2004).
49. Ter-Hovhannisyan, V., Lomsadze, A., Chernoff, Y.O. & Borodovsky, M. Gene prediction in novel fungal genomes using an ab initio algorithm with unsupervised training. *Genome Res.* **18**, 1979–1990 (2008).
50. Cantarel, B.L. *et al*. MAKER: An easy- to-use annotation pipeline designed for emerging model organism genomes. *Genome Res.* **18**, 188–196 (2008).
51. Lee, E., Harris, N., Gibson, M., Chetty, M. & Lewis, S. Apollo: a community resource for genome annotation editing. *Bioinformatics* **25**,1836-1837 (2009).
52. Quevillon, E. *et al*. InterProScan: protein domains identifier. *Nucleic Acids Res.* **33**,W116–120 (2005).
53. Altschul, S.F. *et al*. Protein database searches using compositionally adjusted substitution matrices. *FEBS J.* **272**, 5101-5109 (2005).
54. Käll, L., Krogh, A. & Sonnhammer, E.L. A combined transmembrane topology and signal peptide prediction method. *J. Mol. Biol.* **338**, 1027-1036 (2004).
55. Punta, M. *et al*. The PFAM protein families database. *Nucleic Acids Res.* **40**, D290–301 (2012).
56. Lees, J. *et al.* Gene3D: a domain-based resource for comparative genomics, functional annotation and protein network analysis. *Nucleic Acids Res.* **40**, D465–D471 (2012)
57. Mi, H., Muruganujan, A., Casagrande, J.T. & Thomas, P.D. Large-scale gene function analysis with the PANTHER classification system. *Nat. Prot.* **8**,1551-1566 (2013).
58. Gough, J., Karplus, K., Hughey, R. & Chothia, C. Assignment of homology to genome sequences using a library of hidden Markov models that represent all proteins of known structure. *J. Mol. Biol.* **313**, 903–919 (2001).
59. Attwood, T.K. *et al.* The PRINTS database: a fine-grained protein sequence annotation and analysis resource–its status in 2012. Database (Oxford), bas019 (2012).

60. Sigrist, C.J.A. *et al.* PROSITE, a protein domain database for functional characterization and annotation. *Nucleic Acids Res.* **38**, D161–166 (2010).
61. Conesa, A. *et al.* Blast2GO: a universal tool for annotation, visualization and analysis in functional genomics research. *Bioinformatics* **21**, 3674–3676 (2005).
62. Chandran, D. *et al*. Temporal global expression data reveal known and novel salicylate-impacted processes and regulators mediating powdery mildew growth and reproduction on Arabidopsis. *Plant Physiol.* **149**, 1435–1451 (2009).
63. Benjamini, Y. & Hochberg, Y. Controlling the false discovery rate: a practical and powerful approach to multiple testing. *J. Royal Statist. Soc. B* **57**, 289-300 (1995).
64. Timothy, L. *et al.* MEME SUITE: tools for motif discovery and searching. *Nucleic Acids Res.* **37**,W202–208 (2009).
65. Ludwig-Müller, J., Jülke, S., Bierfreund, N.M., Decker, E.L. & Reski, R. Moss (*Physcomitrella patens*) GH3 proteins act in auxin homeostasis. *New Phytol.* **181**, 323-338 (2009).
66. Staswick, P.E. *et al*. Characterization of an Arabidopsis enzyme family that conjugates amino acids to indole-3-acetic acid. *Plant Cell* **17**, 616–627 (2005).
67. Ehmann, A. The van urk-Salkowski reagent—A sensitive and specific chromogenic reagent for silica gel thin-layer chromatographic detection and identification of indole derivatives. *J. Chromatogr.* **132**, 267–276 (1977).
68. Staswick, P.E. & Tiryaki, I. The oxylipin signal jasmonic acid is activated by an enzyme that conjugates it to isoleucine in Arabidopsis. *Plant Cell* **16**, 2117–2127 (2004).
69. Okrent, R.A., Brooks, M.D. & Wildermuth, M.C. *Arabidopsis* GH3.12 (PBS3) conjugates amino acids to 4-substituted benzoates and is inhibited by salicylate. *J. Biol. Chem.* **284**, 9742–9754 (2009).
70. Finn, R.D., Clements, J. & Eddy, S.R. HMMER web server: interactive sequence similarity searching. *Nucleic Acids Res.* **39**,W29-37 (2011).
71. Gouy, M., Guindon, S. & Gascuel, O. SeaView version 4: a multiplatform graphical user interface for sequence alignment and phylogenetic tree building. *Mol. Biol. Evol*. **27**, 221-224 (2010).
72. Stamatakis, A. RAxML-VI-HPC: Maximum likelihood-based phylogenetic analyses with thousands of taxa and mixed models. *Bioinformatics* **22**, 2688-2690 (2006).
73. Le, S.Q. & Gascuel, O. An improved general amino acid replacement matrix. *Mol. Biol. Evol.* **25**,1307-1320 (2008).
74. Keane, T.M., Creevey, C.J., Pentony, M.M., Naughton, T.J. & McInerney, J.O. Assessment of methods for amino acid matrix selection and their use on empirical data shows that *ad hoc* assumptions for choice of matrix are not justified. *BMC Evol. Biol.* **6**, 29 (2006).
